# Supplementary material for: Ancient European dog genomes reveal continuity since the Early Neolithic
Source: Nat Commun. 2017 Jul 18;8:16082. doi: 10.1038/ncomms16082 (PMC5520058; doi:10.1038/ncomms16082)
Supplement: Supplementary Information [file ncomms16082-s1.pdf]

SI GUIDE

File Name: Supplementary Information
File Description: Supplementary Figures, Supplementary Tables, Supplementary Notes and Supplementary References.

File Name: Peer Review File
File Description:

Supplementary Figure 1

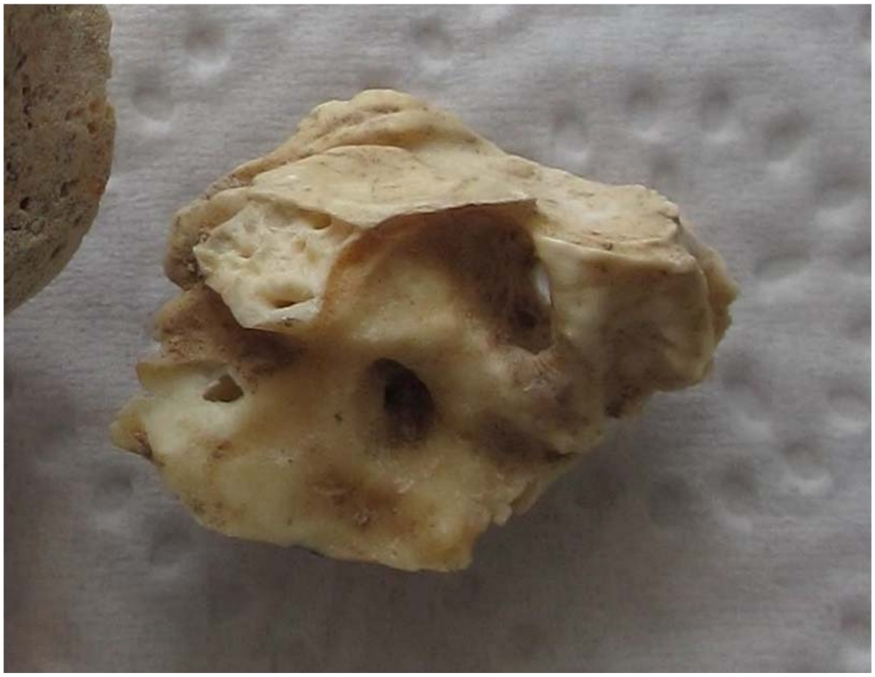

Supplementary Figure 1: HXH petrous bone

Supplementary Figure 2

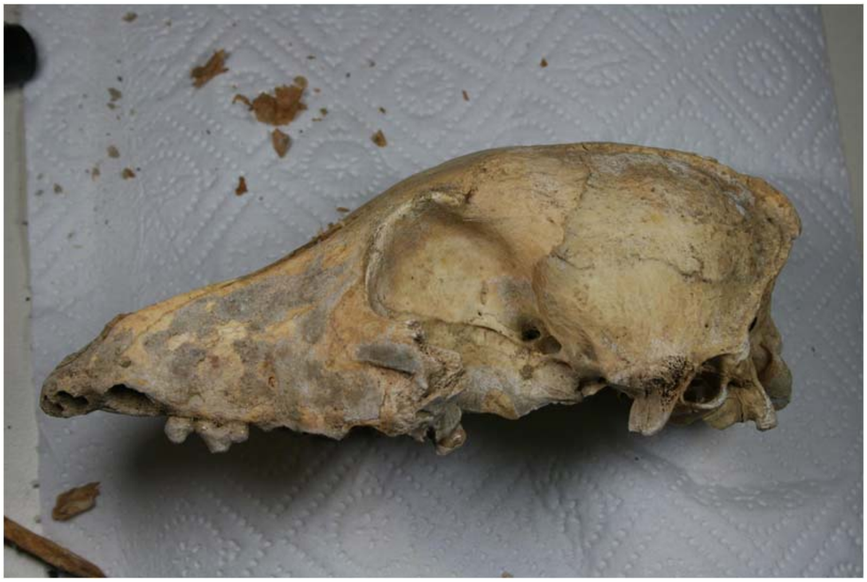

Supplementary Figure 2: CTC cranium.

Supplementary Figure 3

A

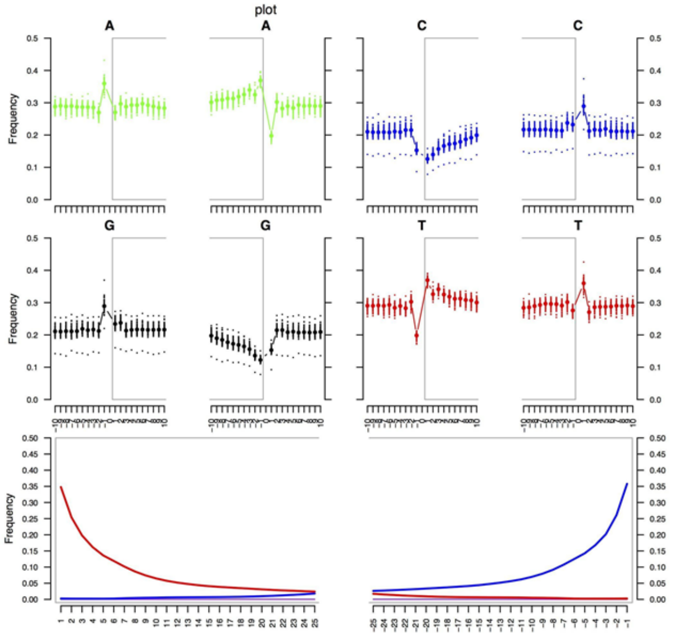

B

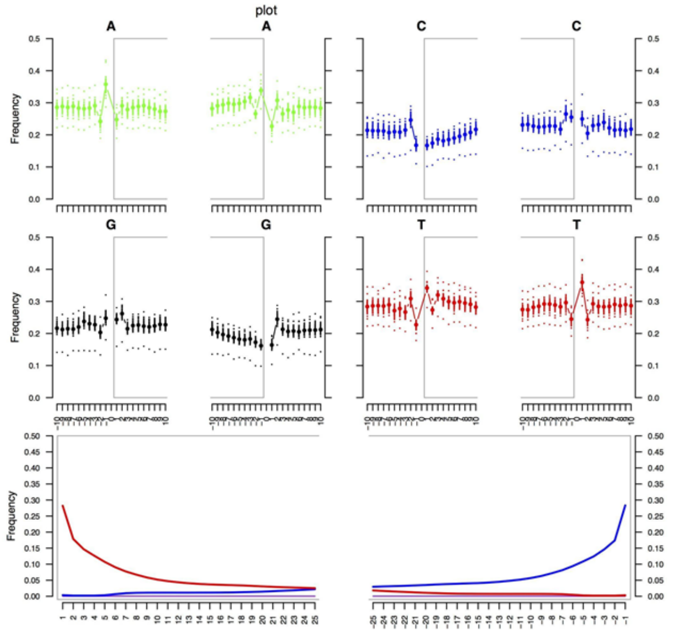

Supplementary Figure 3: MapDamage analysis. Deamination changes typical of ancient DNA are shown for **A)** HXH **B)** CTC.

Supplementary Figure 4

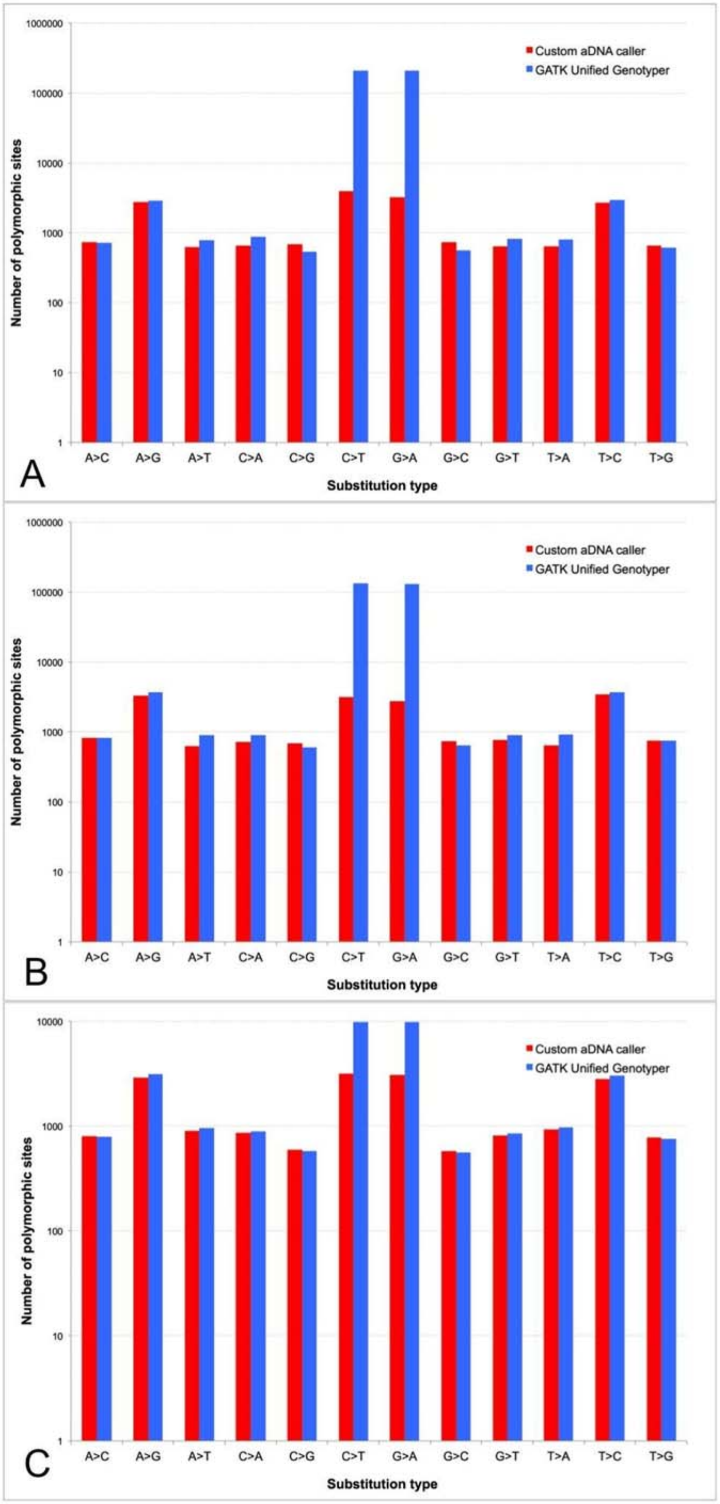

Supplementary Figure 4: Substitution Counts. Comparison of base substitution count with Weibull-based caller (red) and the `GATKUnified Genotyper caller` (blue). A) HXH, B) CTC, C) NGD. (Note Y axis is log-scaled).

Supplementary Figure 5

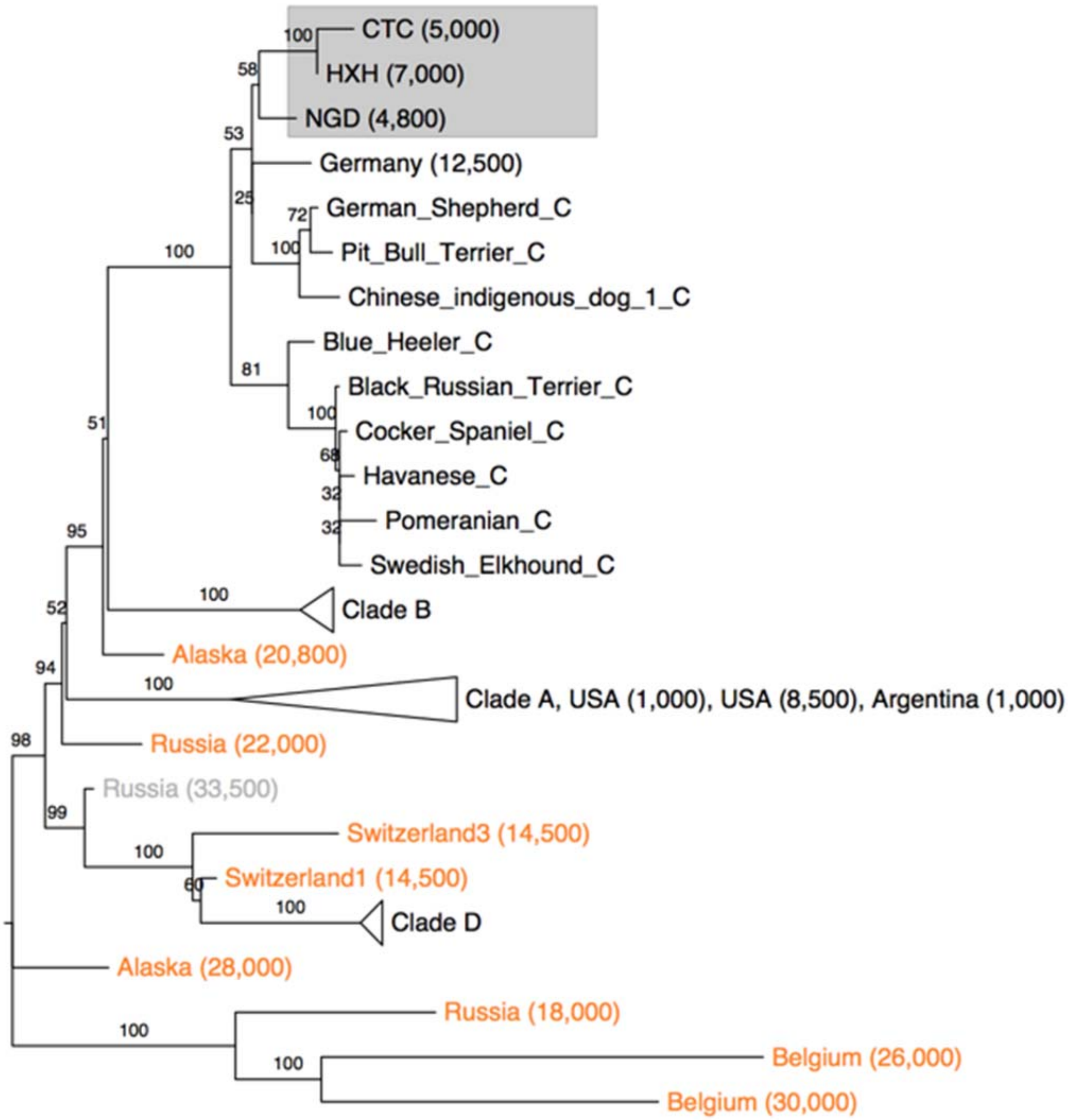

Supplementary Figure 5. NJ tree depicts phylogenetic relationship of CTC and HXH mitochondrial haplotypes with modern dogs and ancient canids. Bootstrap values are drawn at the branches. Clade membership of modern dogs (A-D) are written into the sample name after the underscore and year indicating the approximate reported age is written after the ancient sample names. HXH, CTC, and NGD are boxed in light grey. Dogs, wolves, and ambiguous taxonomic classifications are labeled black, orange, and grey, respectively.

Supplementary Figure 6

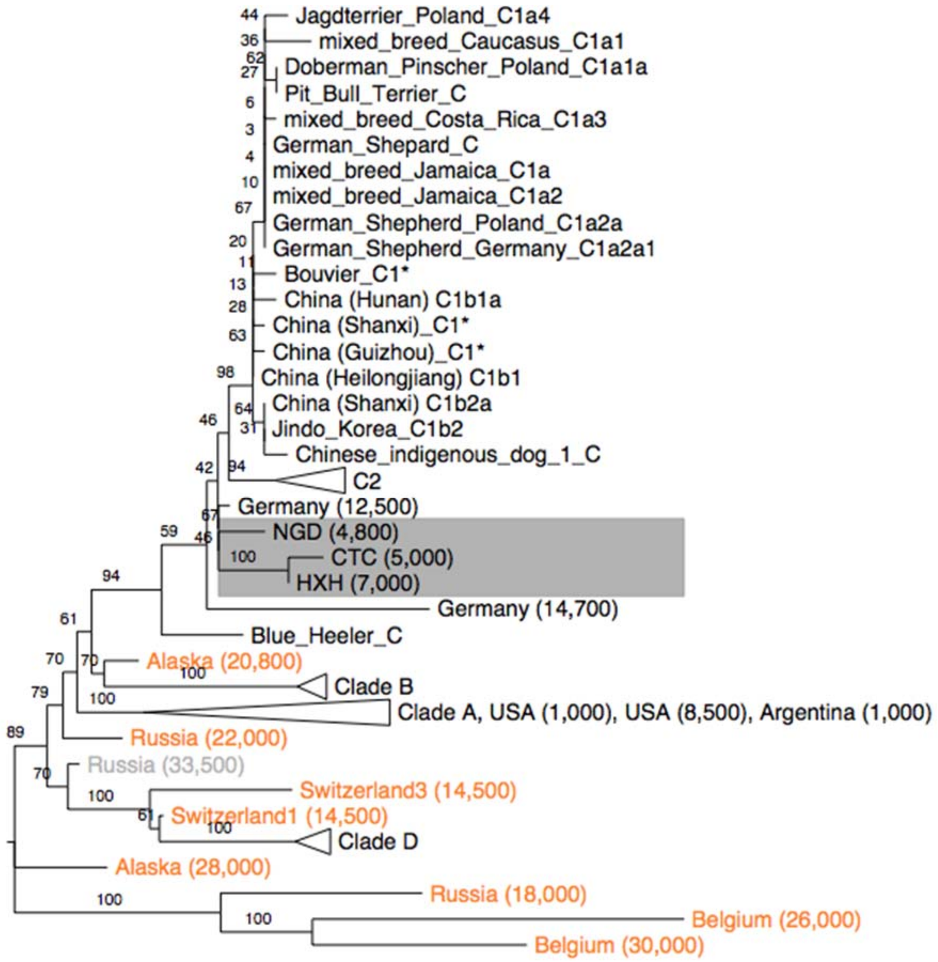

Supplementary Figure 6. NJ tree depicts phylogenetic relationship of CTC and HXH mitochondrial haplotypes with low coverage Bonn Oberkassel ancient dog. Bootstrap values are drawn at the branches. Clade membership of modern dogs (A-D) are written into the sample name after the underscore and year indicating the approximate reported age is written after the ancient sample names. Dogs, wolves, and ambiguous taxonomic classifications are labeled black, orange, and grey, respectively. Mitochondrial haplogroups annotated by Duleba *et al.* are written after the final underscore following the sample name. The asterisk (*) following the haplogroup of three C1 dogs indicates their haplogroups are not representative of C1a and C1b. Dogs, wolves, and ambiguous taxonomic classifications are labeled black, orange, and grey, respectively. The Bonn Oberkassel dog is labeled as Germany (14,700).

Supplementary Figure 7

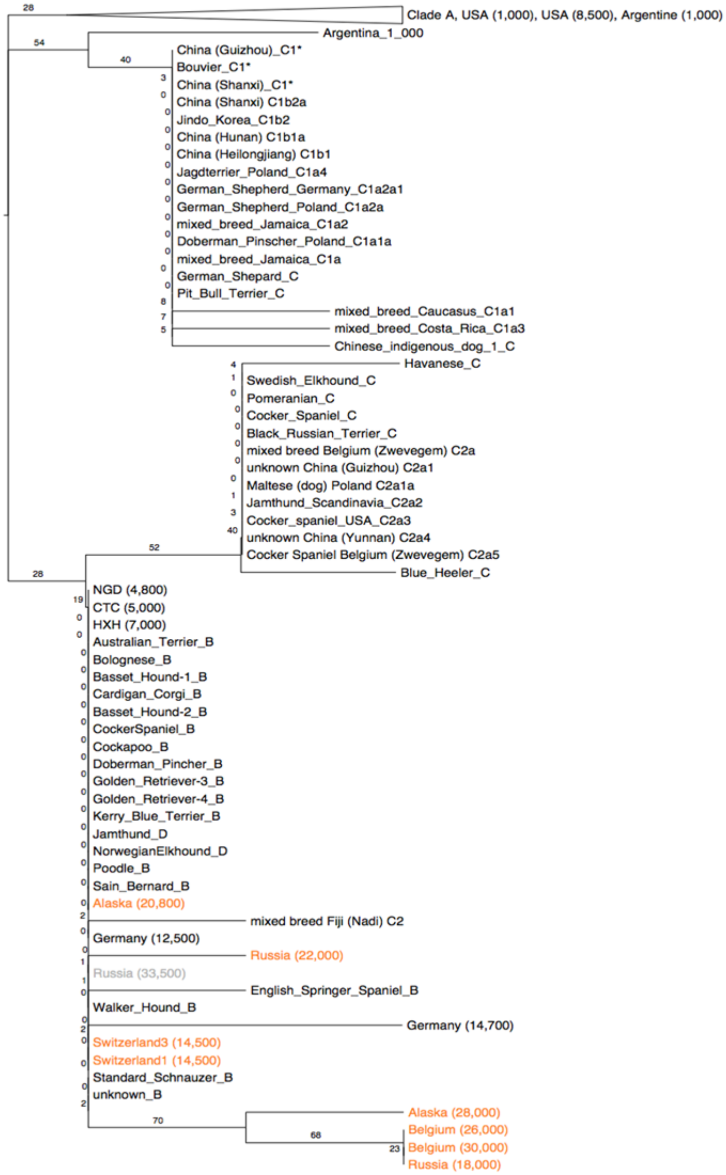

Supplementary Figure 7. NJ tree depicts phylogenetic relationship of CTC and HXH mitochondrial haplotypes with low coverage Bonn Oberkassel ancient dog (transversions only). Bootstrap values are drawn at the branches. Clade membership of modern dogs (A-D) are written into the sample name after the underscore and year indicating the approximate reported age is written after the ancient sample name. Dogs, wolves, and ambiguous taxonomic classifications are labeled black, orange, and grey, respectively. Mitochondrial haplogroups annotated by Duleba *et al.* are written after the final underscore following the sample name. The asterisk (*) following the haplogroup of three C1 dogs indicates their haplogroups are not representative of C1a and C1b. Dogs, wolves, and ambiguous taxonomic classifications are labeled black, orange, and grey, respectively. The Bonn Oberkassel dog is labeled as Germany (14,700).

Supplementary Figure 8

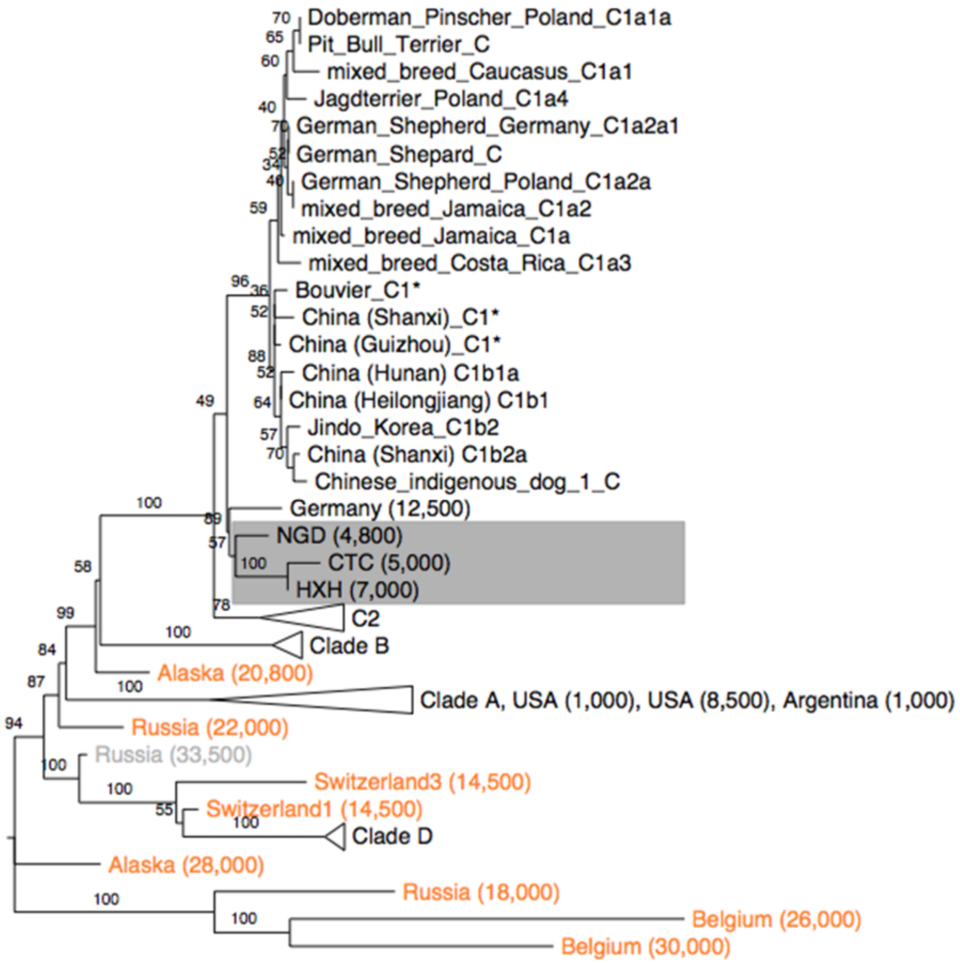

Supplementary Figure 8: NJ tree depicts phylogenetic relationship of CTC and HXH mitochondrial haplotypes with 24 additional Clade C mitochondria samples for granular haplogroup identification. Bootstrap values are drawn at the branches. Clade membership of modern dogs (A-D) are written into the sample name after the underscore and year indicating the approximate reported age is written after the ancient sample names. HXH, CTC and NGD are boxed in light grey. Mitochondrial haplogroups annotated by Duleba *et al.* are written after the final underscore following the sample name. The asterisk (*) following the haplogroup of three C1 dogs indicates their haplogroups are not representative of C1a and C1b. Dogs, wolves, and ambiguous taxonomic classifications are labeled black, orange, and grey, respectively.

Supplementary Figure 9

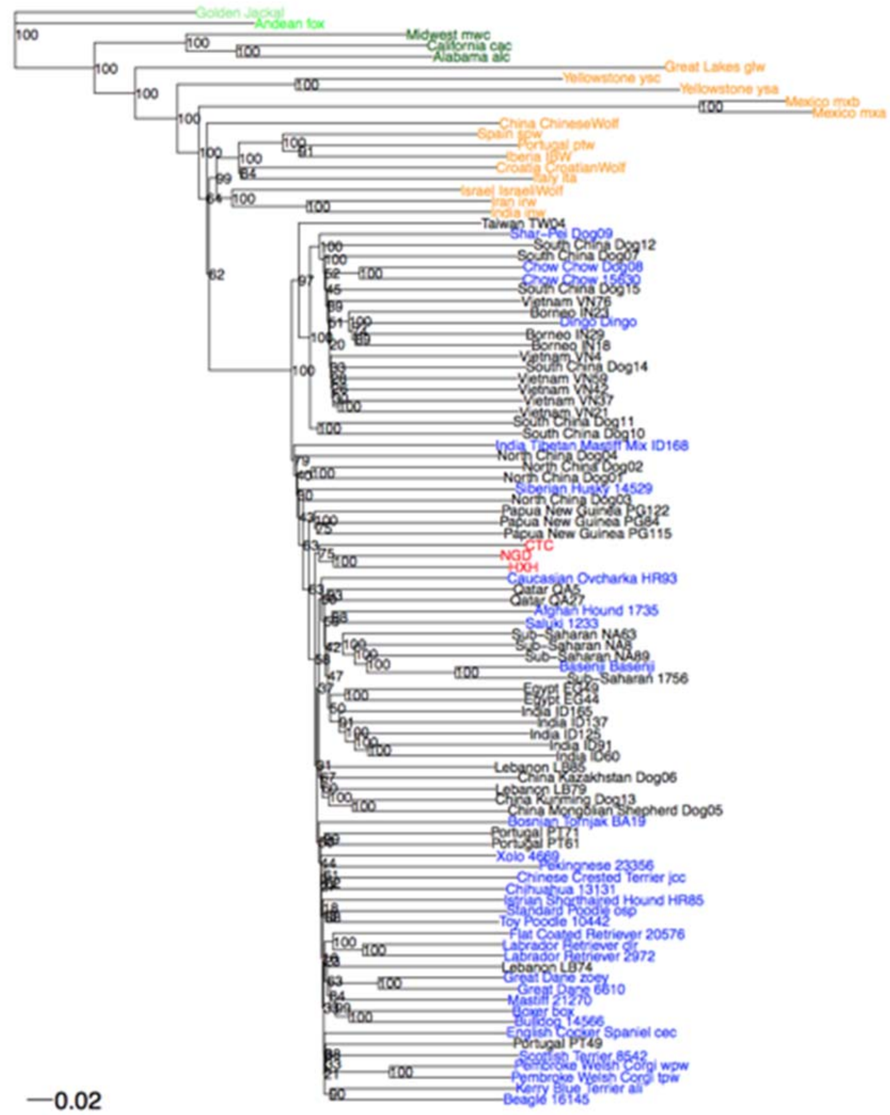

Supplementary Figure 9: NJ tree. NJ tree based on pairwise genetic distances using whole genome snp set of 99 samples, including Andean fox (green), golden jackal (light green), coyotes (dark green), wolves (orange), ancient samples (red), village dogs (purple), and breed dogs (blue).

Supplementary Figure 10

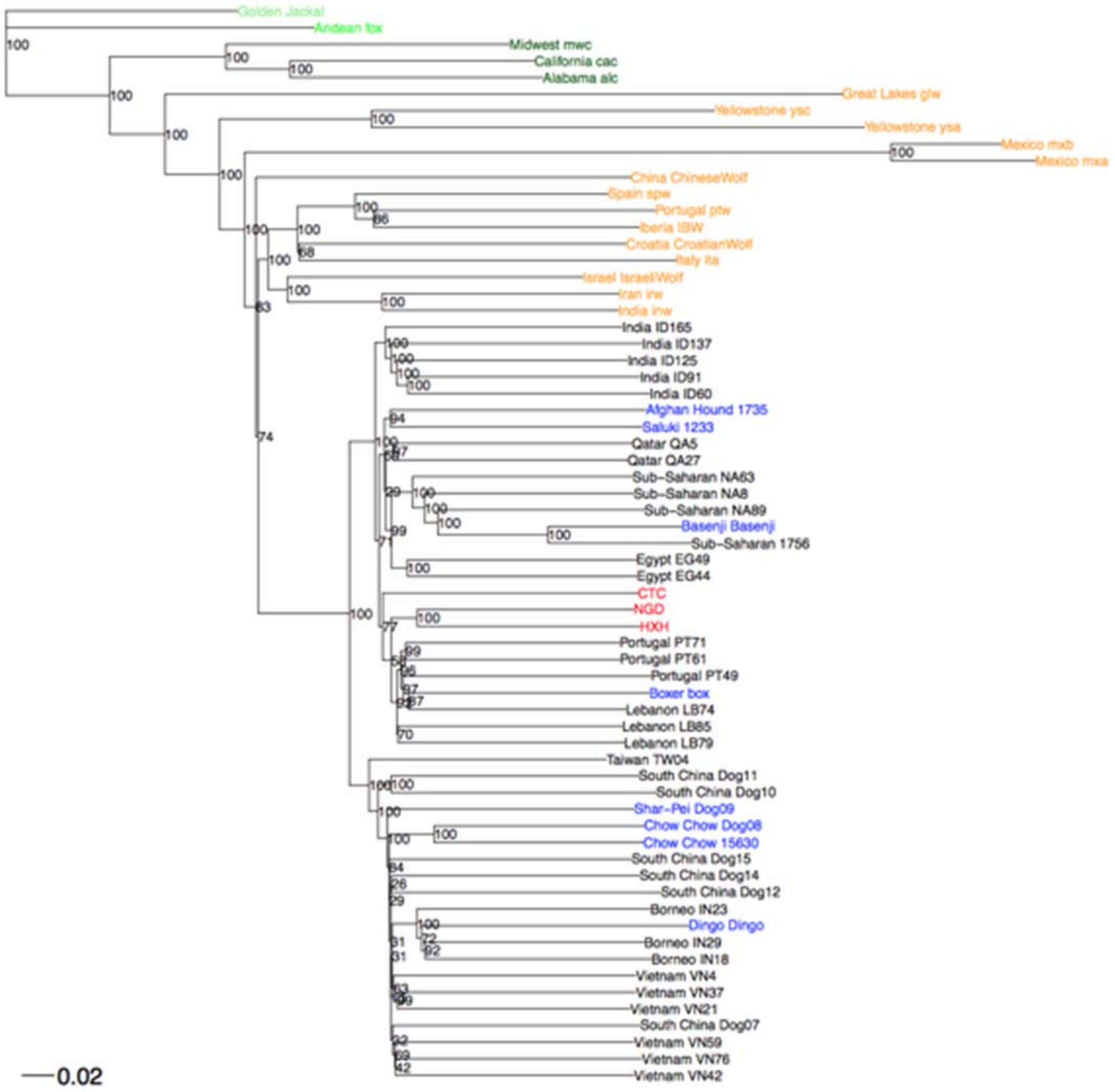

Supplementary Figure 10: NJ tree. NJ tree based on pairwise genetic distances using whole genome snp set of 65 samples, including andean fox (green), golden jackal (light green), coyotes (dark green), wolves (orange), ancient samples (red), village dogs (purple), and breed dogs (blue). Village dogs from north China and Papua New Guinea and most European breeds are removed from this set.

Supplementary Figure 11

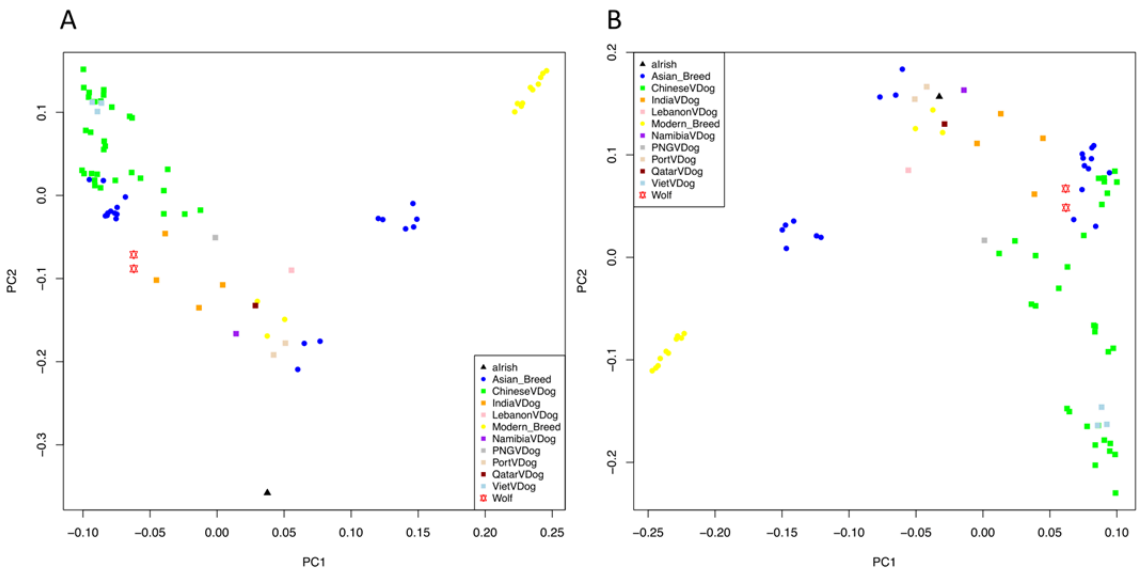

Supplementary Figure 11: PCA with and without doubling Newgrange dog from Frantz *et al.* . A) Principal component analysis based on 269,512 transversions ascertained solely in the genome-wide data-set from Frantz *et al.* where NGD dog (aIrish) is doubled (with and without quality score re-calibration), equivalent to Figure S9 in Frantz *et al.*. B) Principal component analysis based on 269,512 transversion ascertained solely in the genome-wide data-set from Frantz *et al.* where only the NGD dog is retained, without quality score recalibration.

Supplementary Figure 12

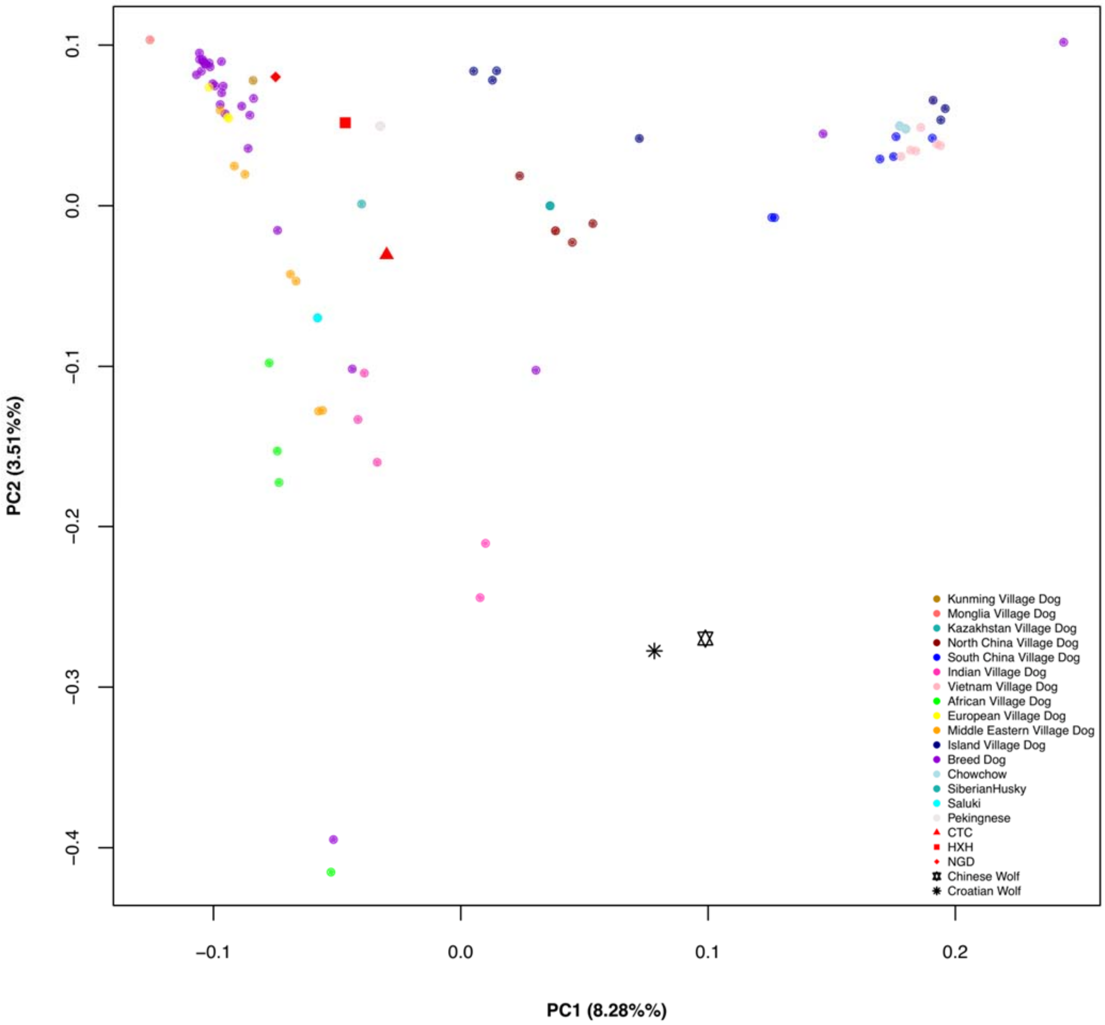

Supplementary Figure 12: PCA using SNPs from Frantz *et al.* on our dataset. A. Principal component analysis on our samples based on 269,512 transversions ascertained solely in the genome-wide data-set from Frantz *et al.* . We included all the dogs from our sample set and chose two wolves, a Chinese wolf and Croatian wolf.

Supplementary Figure 13

A

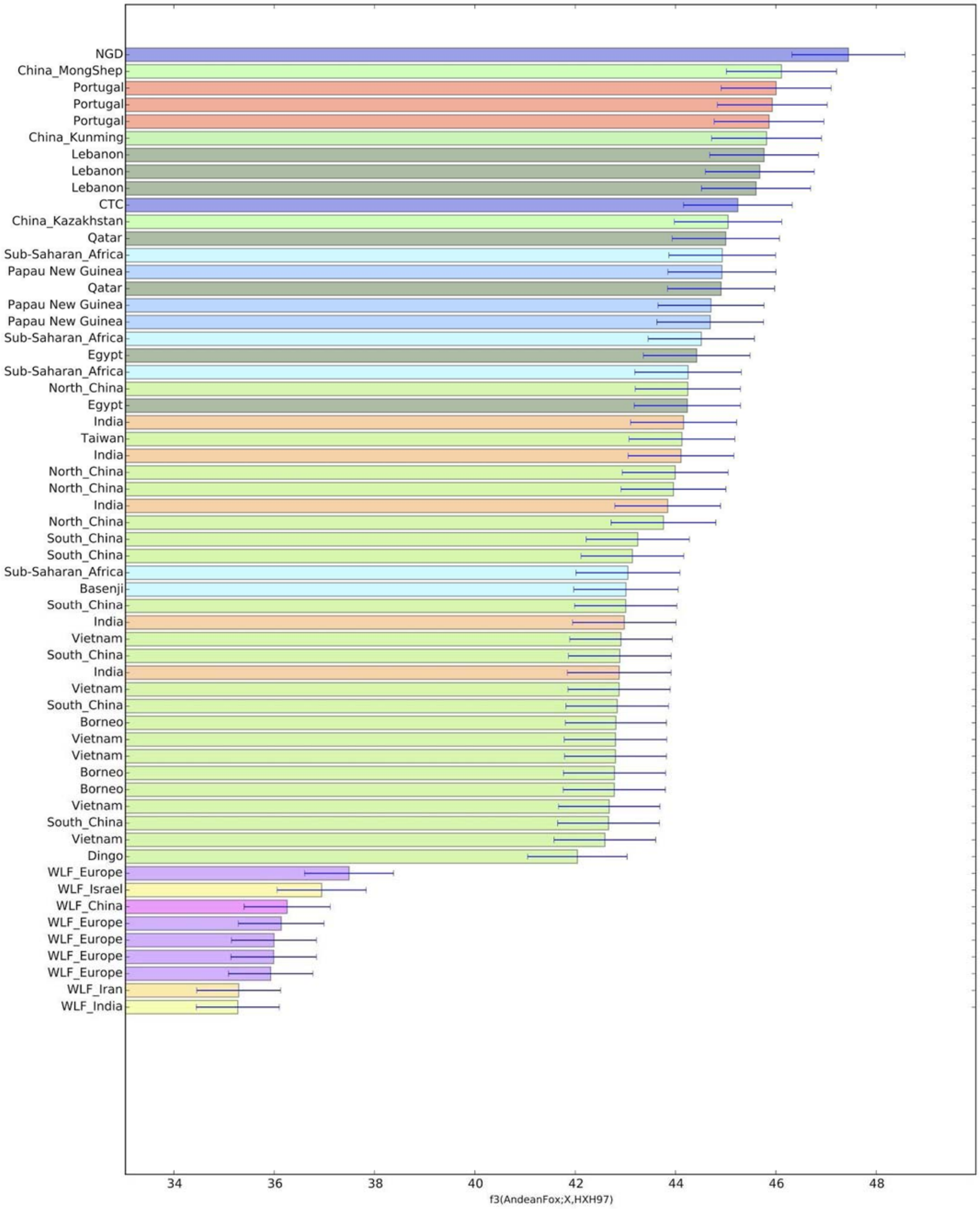

B

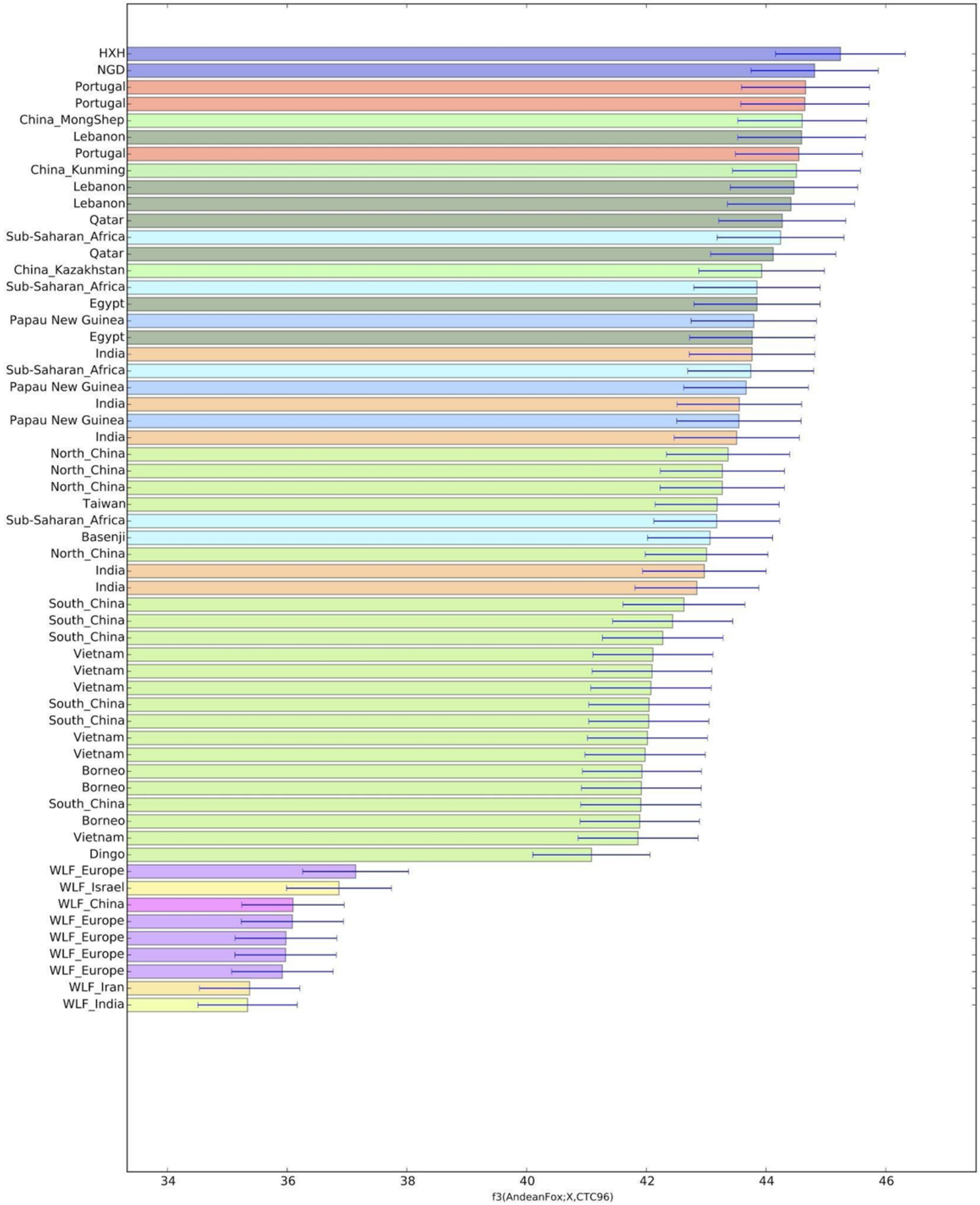
HXH
NGD
Portugal
Portugal
China_MongShep
Lebanon
Portugal
China_Kunming
Lebanon
Lebanon
Qatar
Sub-Saharan_Africa
Qatar
China_Kazakhstan
Sub-Saharan_Africa
Egypt
Papau New Guinea
Egypt
India
Sub-Saharan_Africa
Papau New Guinea
India
Papau New Guinea
India
North_China
North_China
North_China
Taiwan
Sub-Saharan_Africa
Basenji
North_China
India
India
South_China
South_China
South_China
Vietnam
Vietnam
Vietnam
South_China
South_China
Vietnam
Vietnam
Borneo
Borneo
South_China
Borneo
Vietnam
Dingo
WLF_Europe
WLF_Israel
WLF_China
WLF_Europe
WLF_Europe
WLF_Europe
WLF_Europe
WLF_Iran
WLF_India
34
36
38
40
42
44
46
f3(AndeanFox;X,CTC96)

C

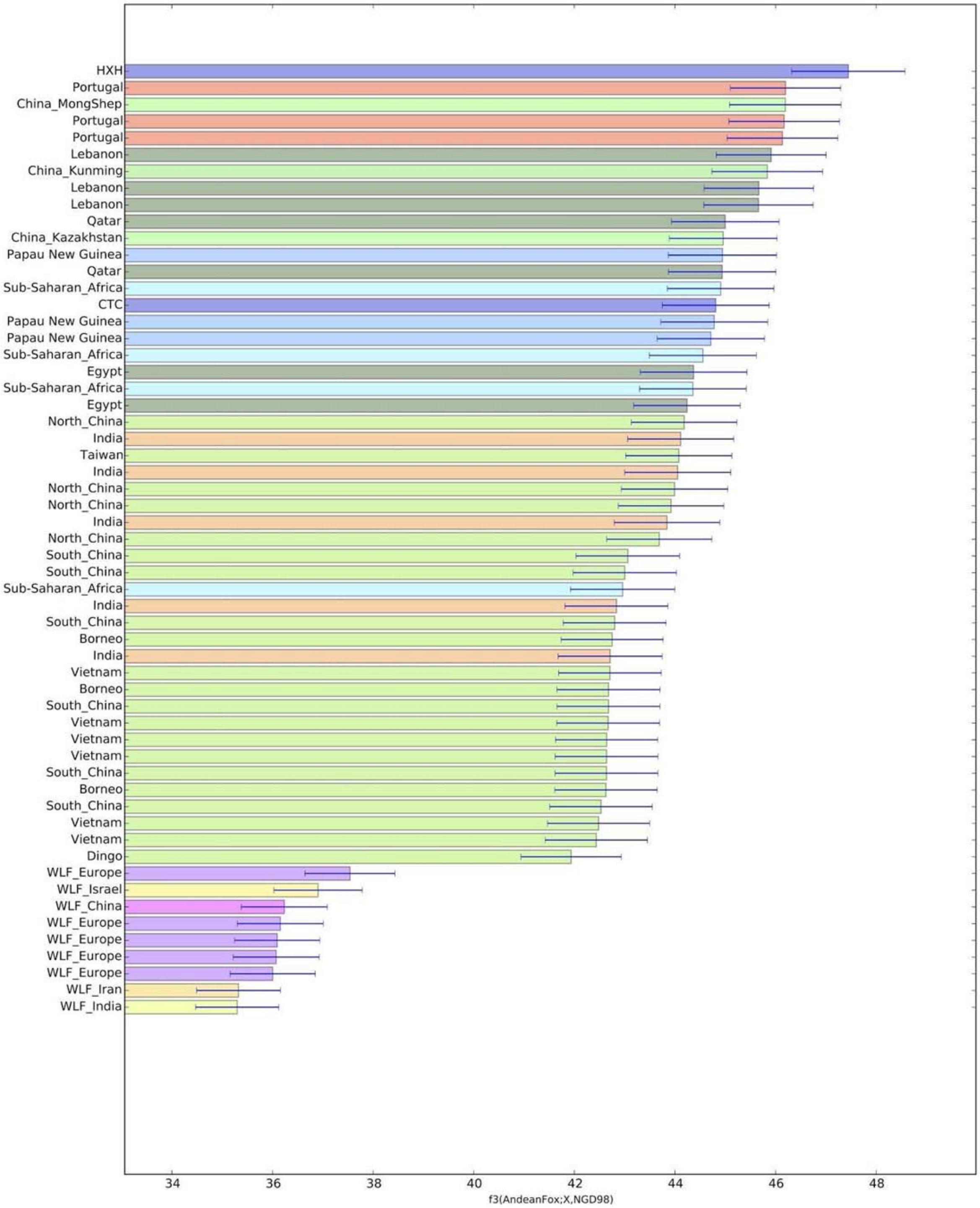

Supplementary Figure 13: Outgroup *f3*-analysis using the whole genome SNP dataset. A) HXH **B)** CTC **C)** NGD

Supplementary Figure 14

A

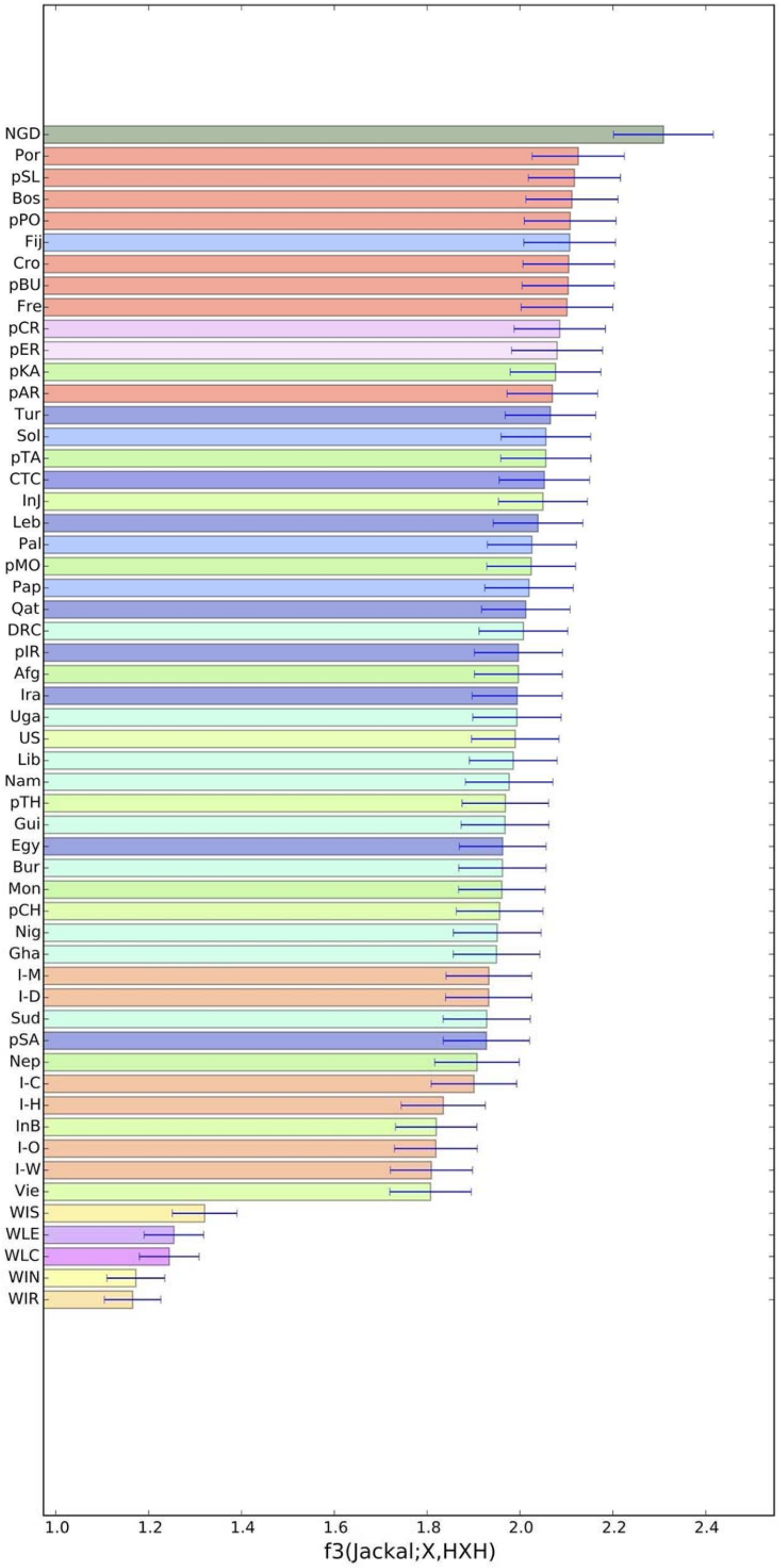

B

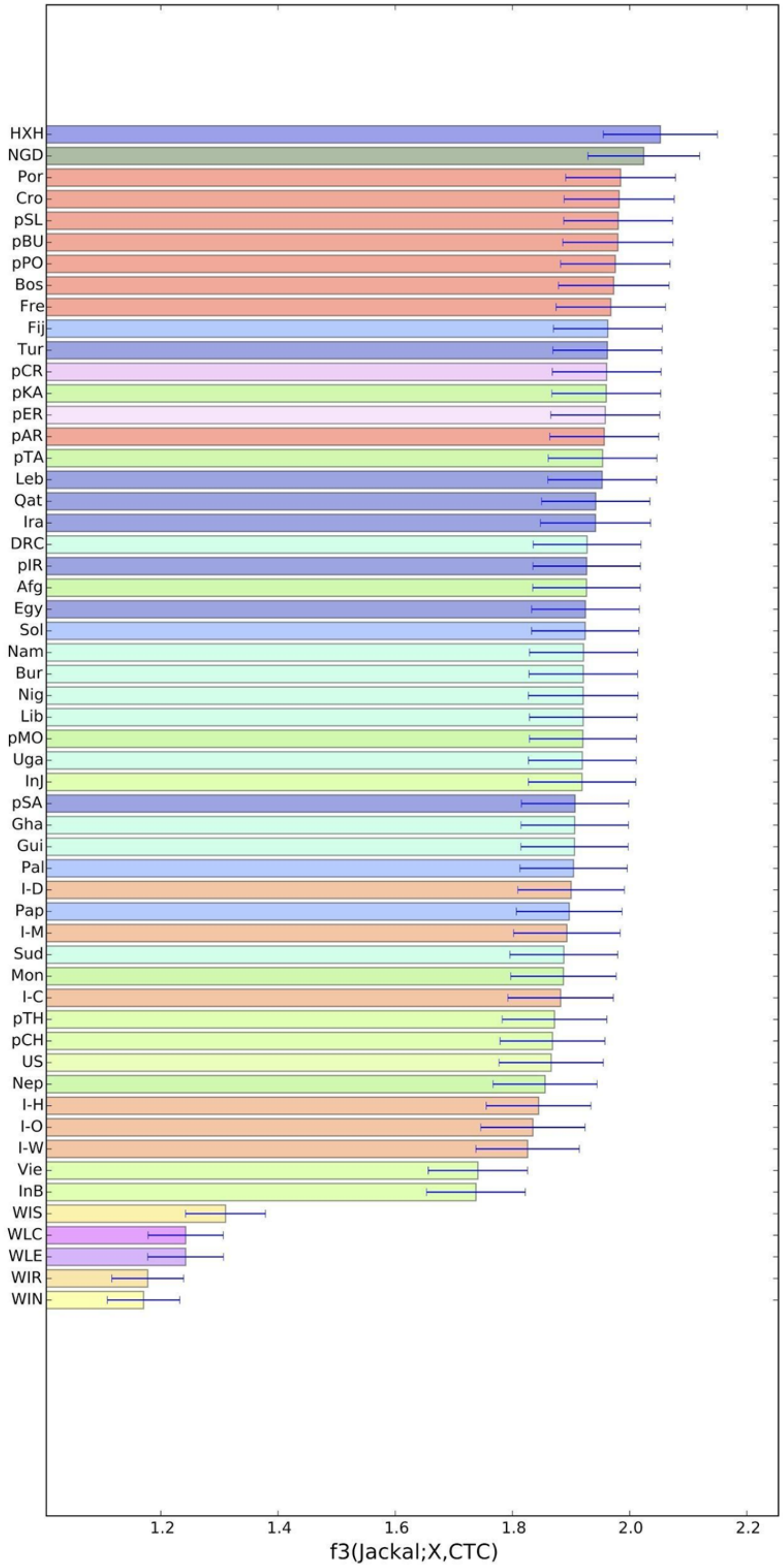
HXH
NGD
Por
Cro
pSL
pBU
pPO
Bos
Fre
Fij
Tur
pCR
pKA
pER
pAR
pTA
Leb
Qat
Ira
DRC
pIR
Afg
Egy
Sol
Nam
Bur
Nig
Lib
pMO
Uga
InJ
pSA
Gha
Gui
Pal
I-D
Pap
I-M
Sud
Mon
I-C
pTH
pCH
US
Nep
I-H
I-O
I-W
Vie
InB
WIS
WLC
WLE
WIR
WIN
1.2
1.4
1.6
1.8
2.0
2.2
f3(Jackal;X,CTC)

C

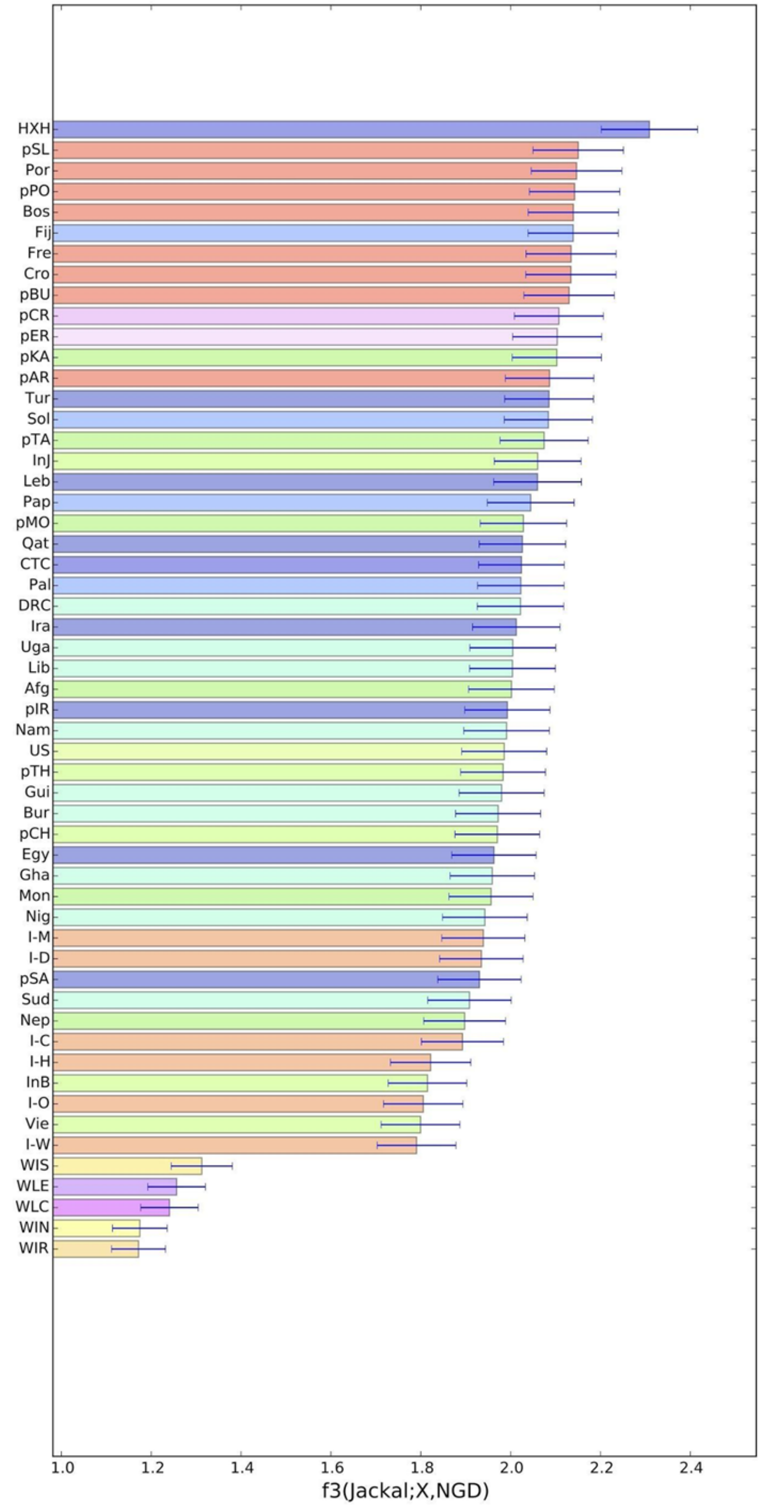

Supplementary Figure S14: Outgroup *f3*-analysis using genotype SNP array dataset. A) HXH **B)** CTC **C)** NGD

Supplementary Figure 15

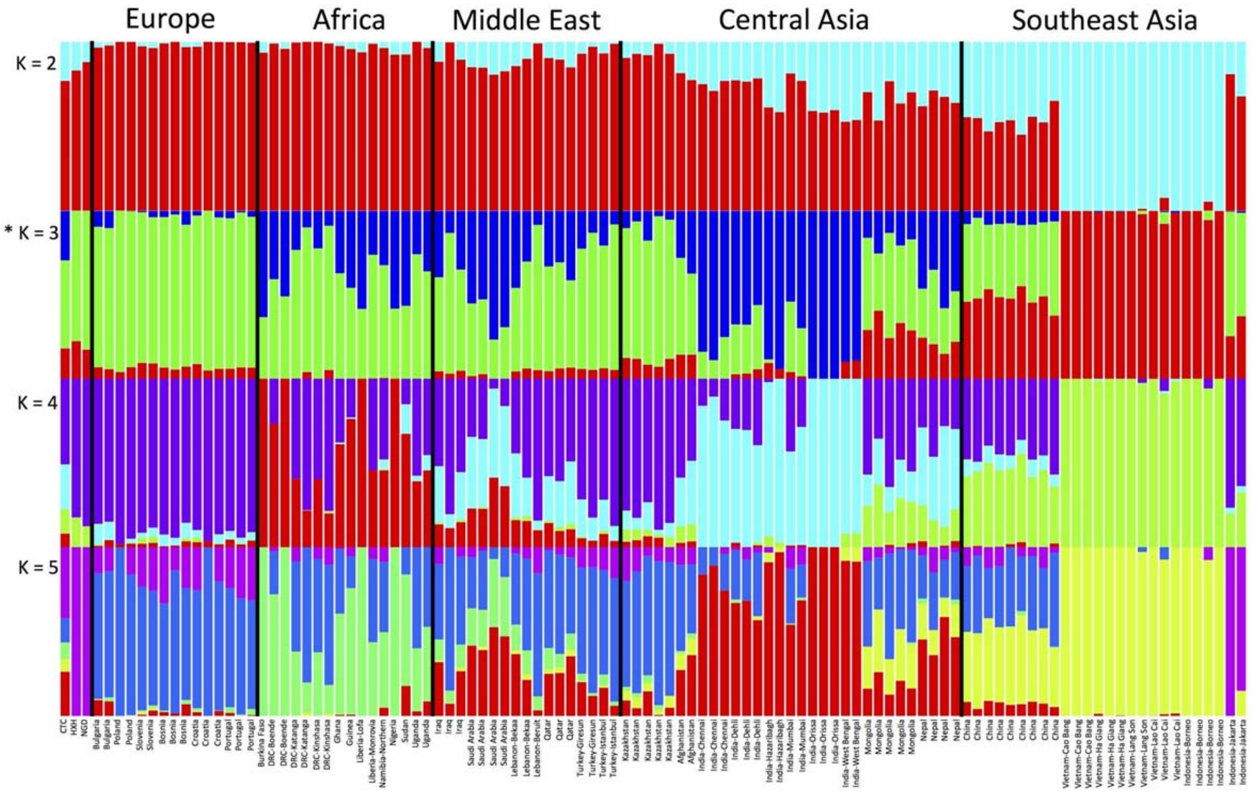

Supplementary Figure 15: `ADMIXTURE` analysis. Results shown for K = 2 through 5 for a global representation of village dogs, CTC, HXH and NGD. Vertical lines represent individual dogs.

Supplementary Figure 16

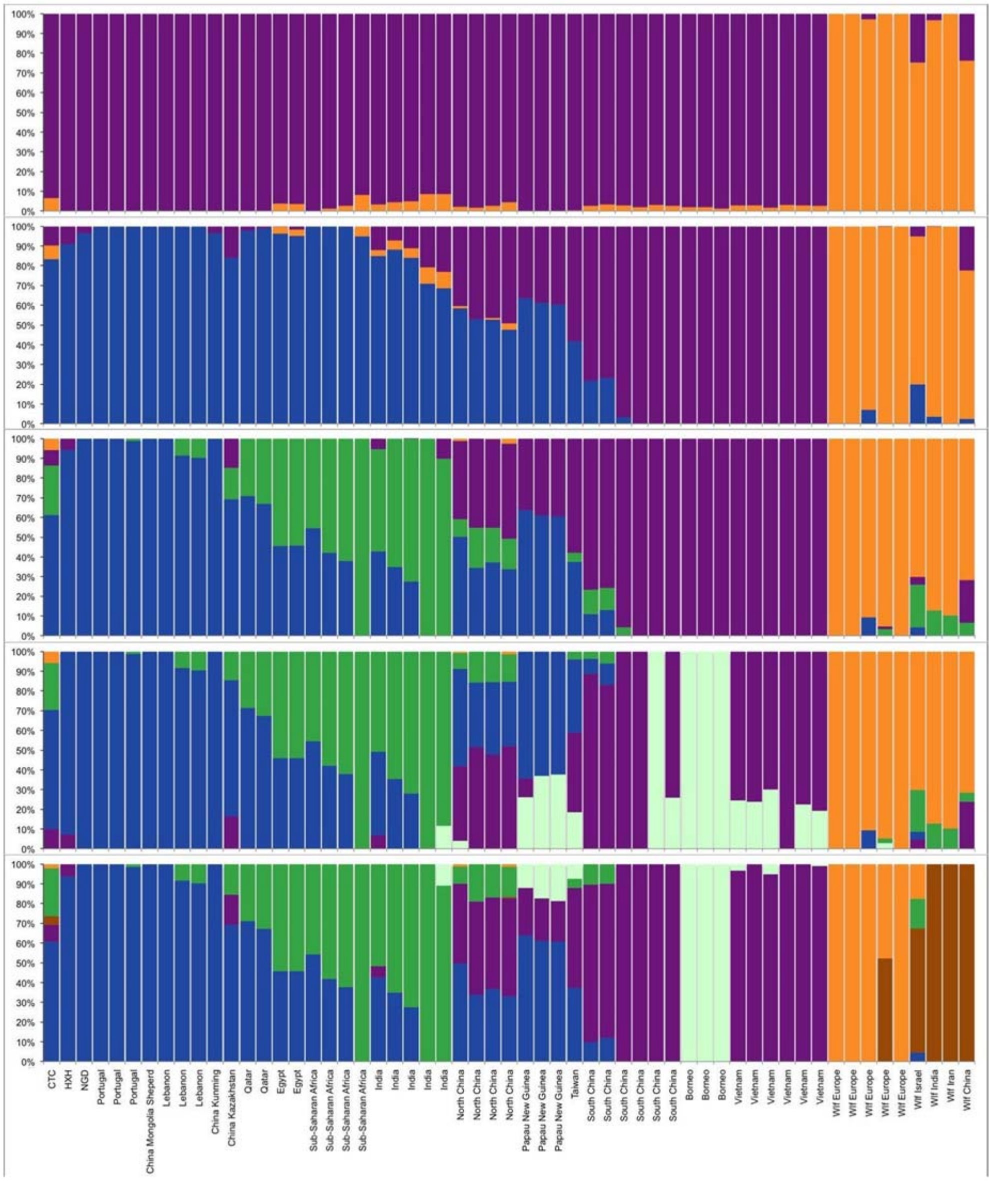

Supplementary Figure 16. `NGSadmix` analysis. Shown results for clustering for *K*=2 to 6 ancestry components.

Supplementary Figure 17

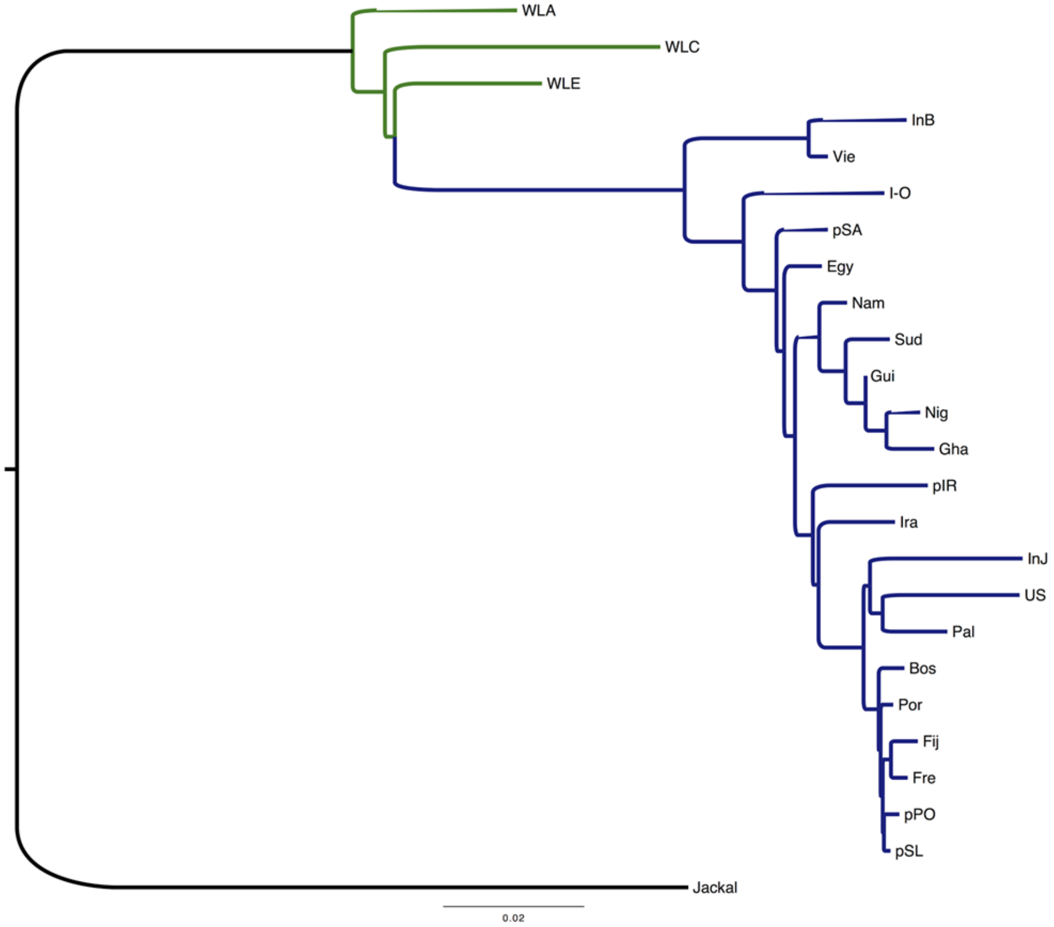

Supplementary Figure 17 NJ tree for the genotype SNP dataset were admixed populations detected using an *f3*-statistic have been excluded.

Supplementary Figure 18

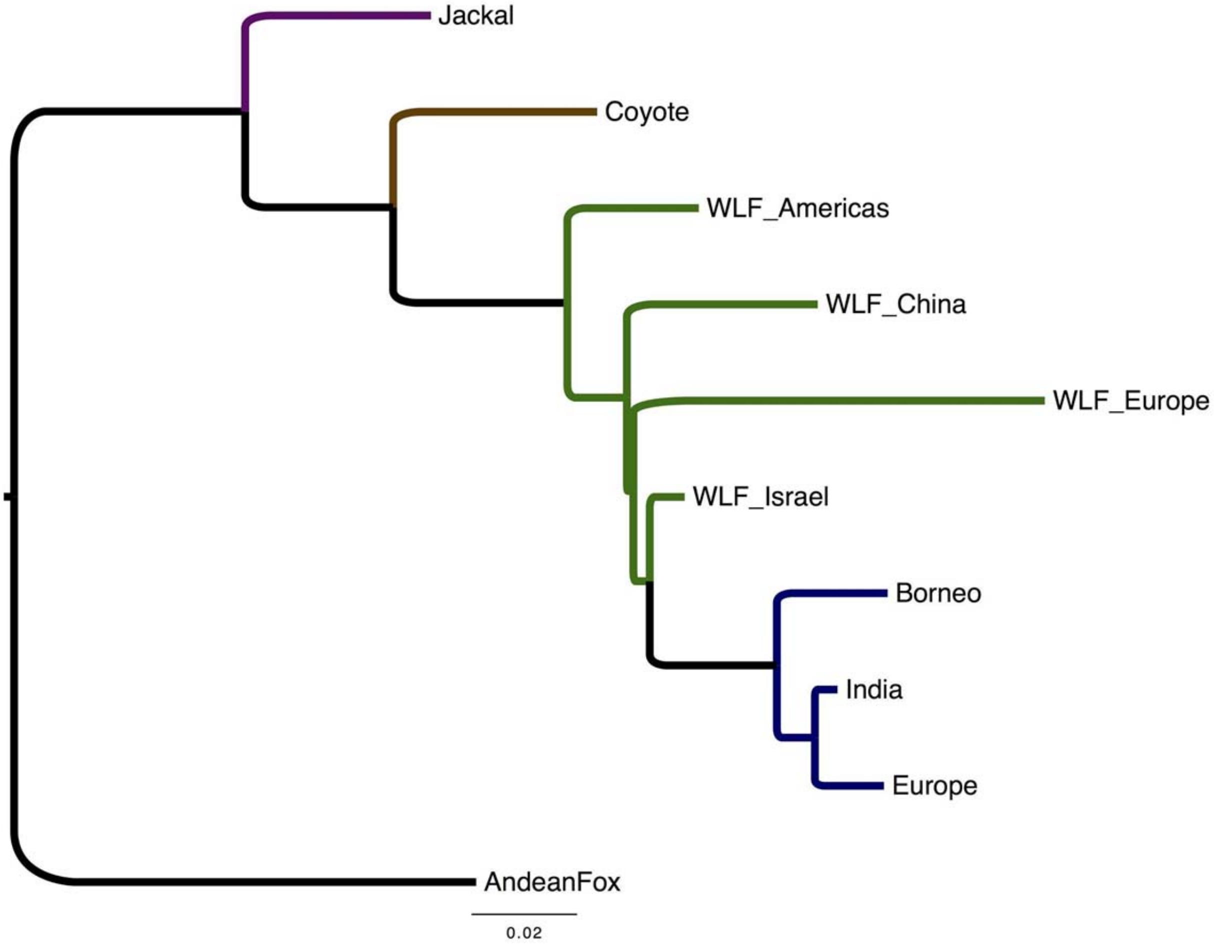

Supplementary Figure 18: NJ tree for the whole genome SNP dataset were admixed populations detected using an *f3*-statistic have been excluded.

Supplementary Figure 19

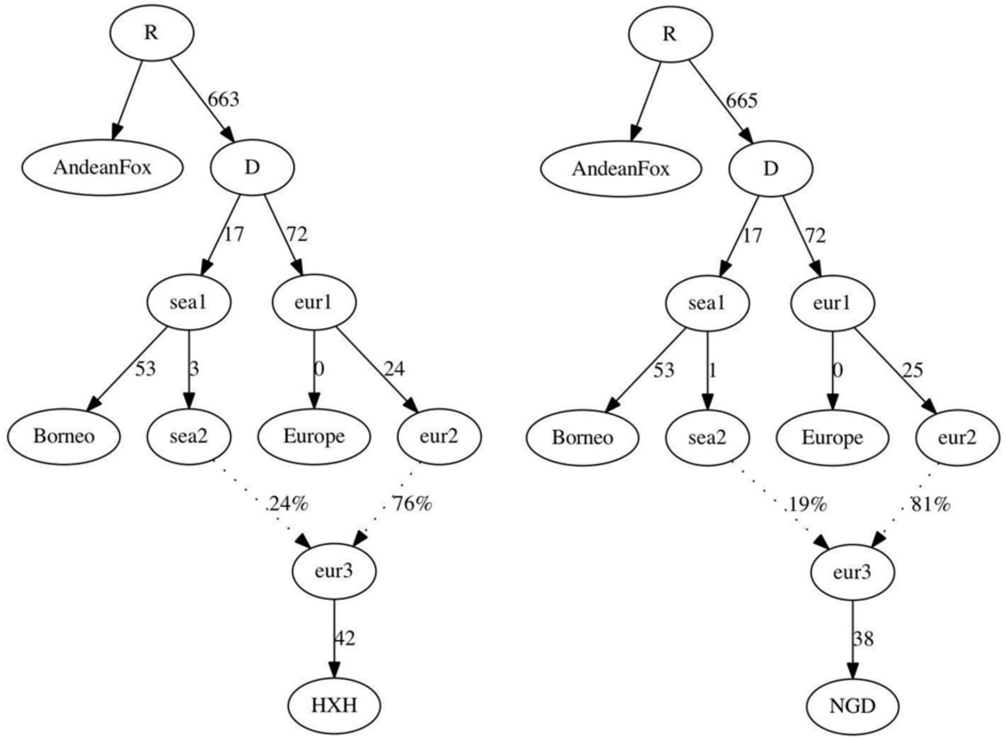

Supplementary Figure 19: Best fit admixture graph for HXH (left) and NGD (right) when including modern European and Southeast Asian village dogs.

Supplementary Figure 20

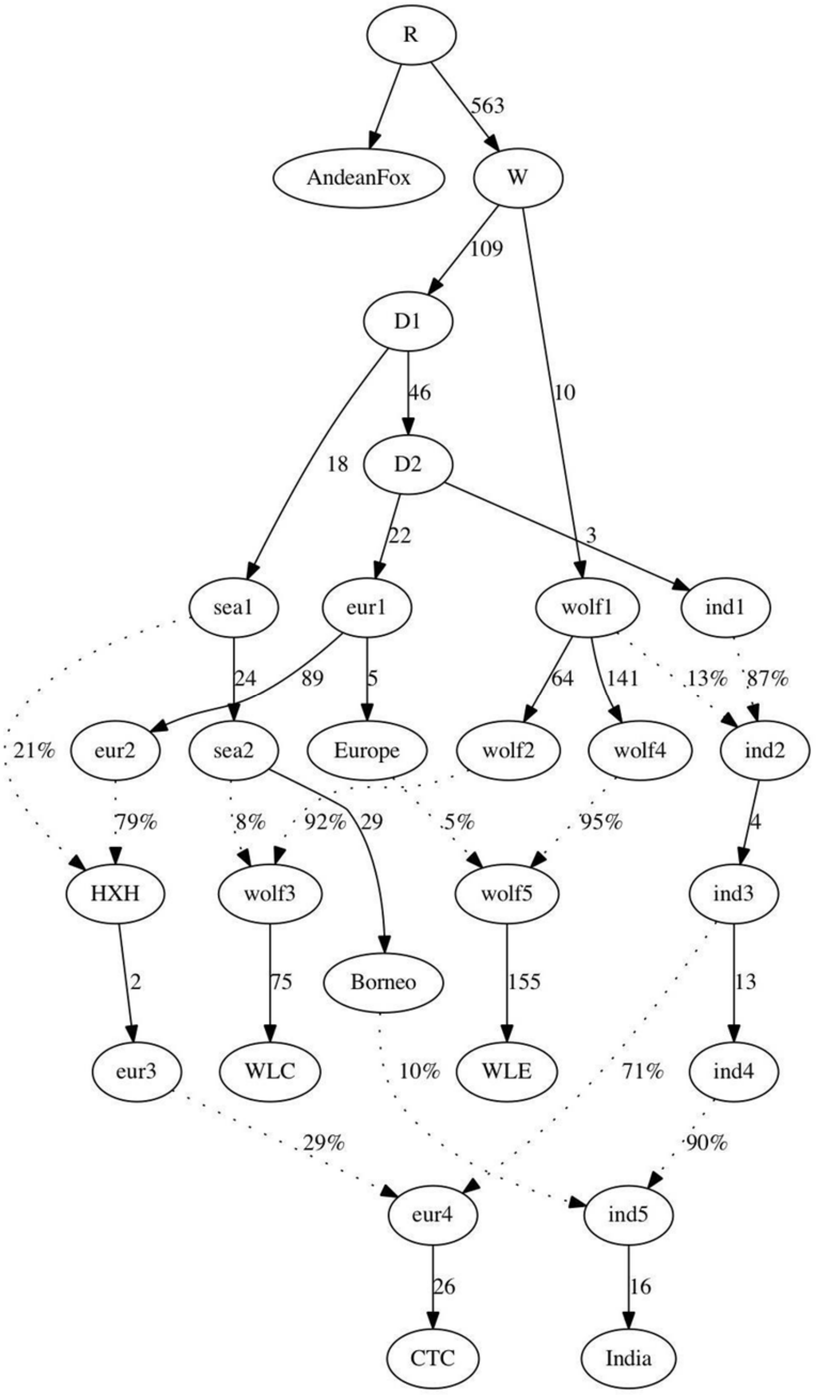

Supplementary Figure 20: Admixture graph incorporating HXH, CTC, modern village dogs and wolves

Supplementary Figure 21

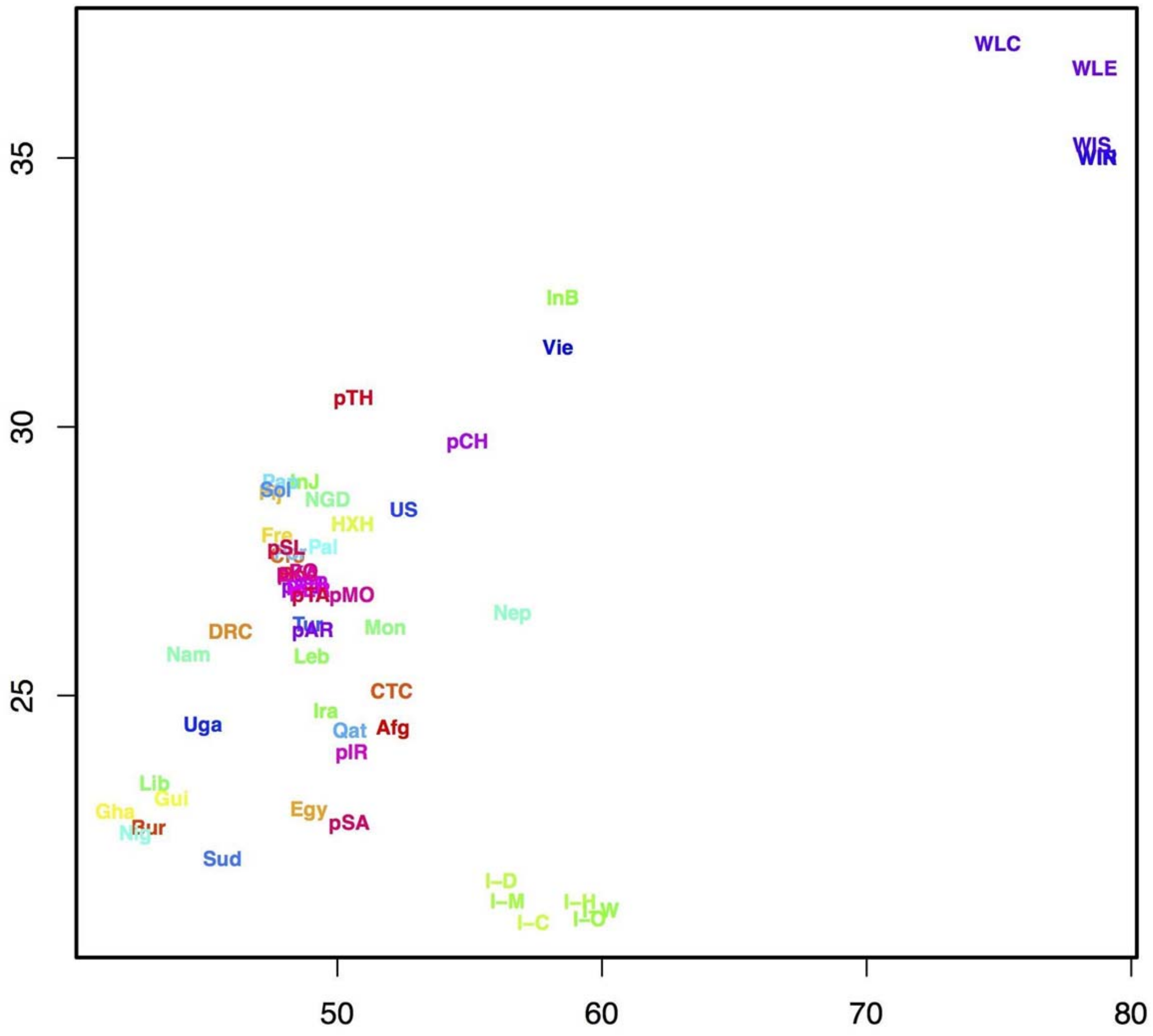

Supplementary Figure 21: Spacemix results. `Spacemix` geogenetic space of wolves, modern dogs and CTC (orange), HXH (light green) and NGD (green).

Supplementary Figure 22

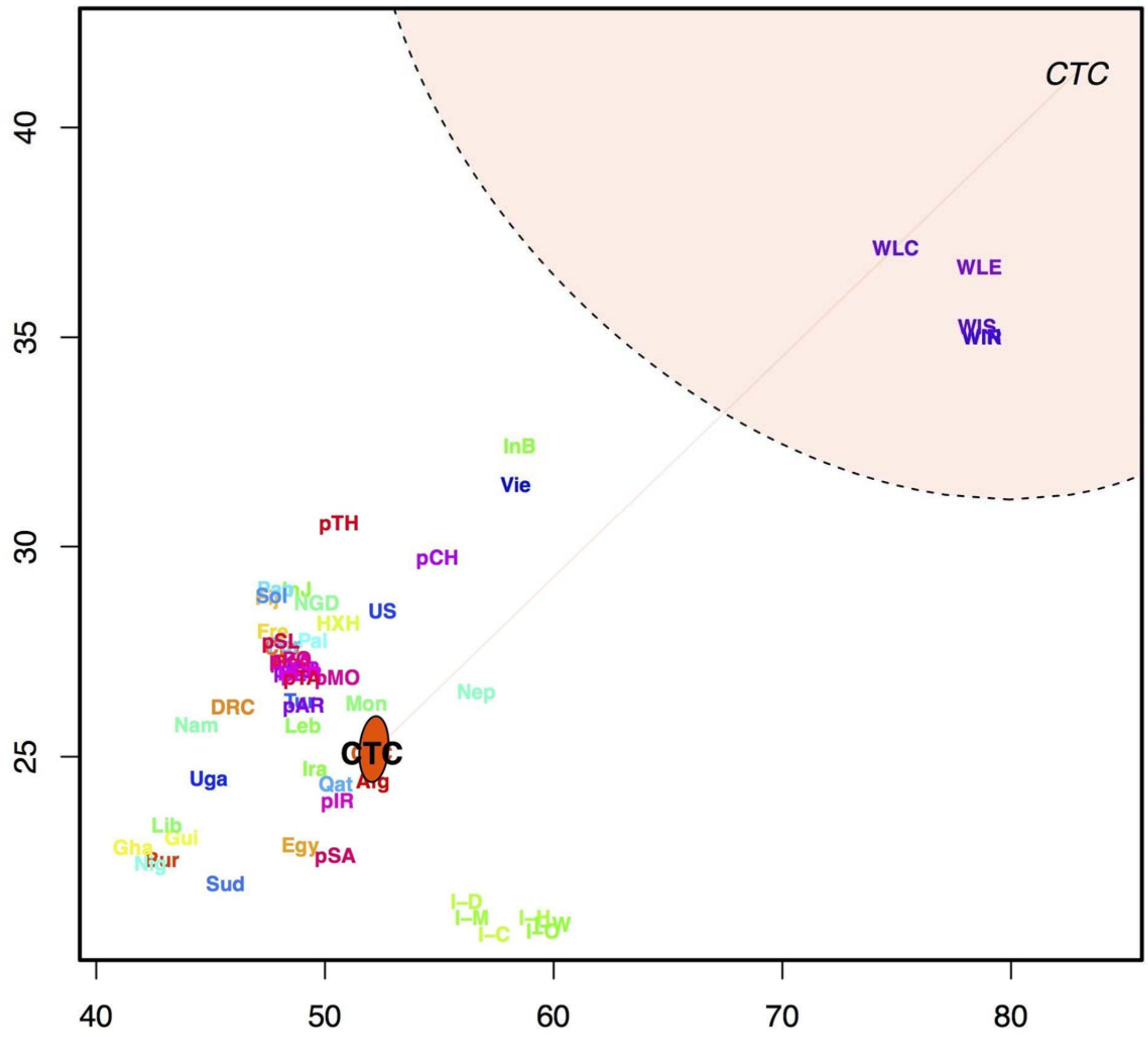

Supplementary Figure 22: CTC - Wolf admixture as inferred by `SpaceMix`. CTC in bold type reflects the samples geogenetic position (95% CI solid orange ellipse), *CTC* in italics reflects the geogenetic position of the proposed source of admixture into CTC, with an estimated value of 9% (95% CI transparent orange ellipse).

Supplementary Figure 23

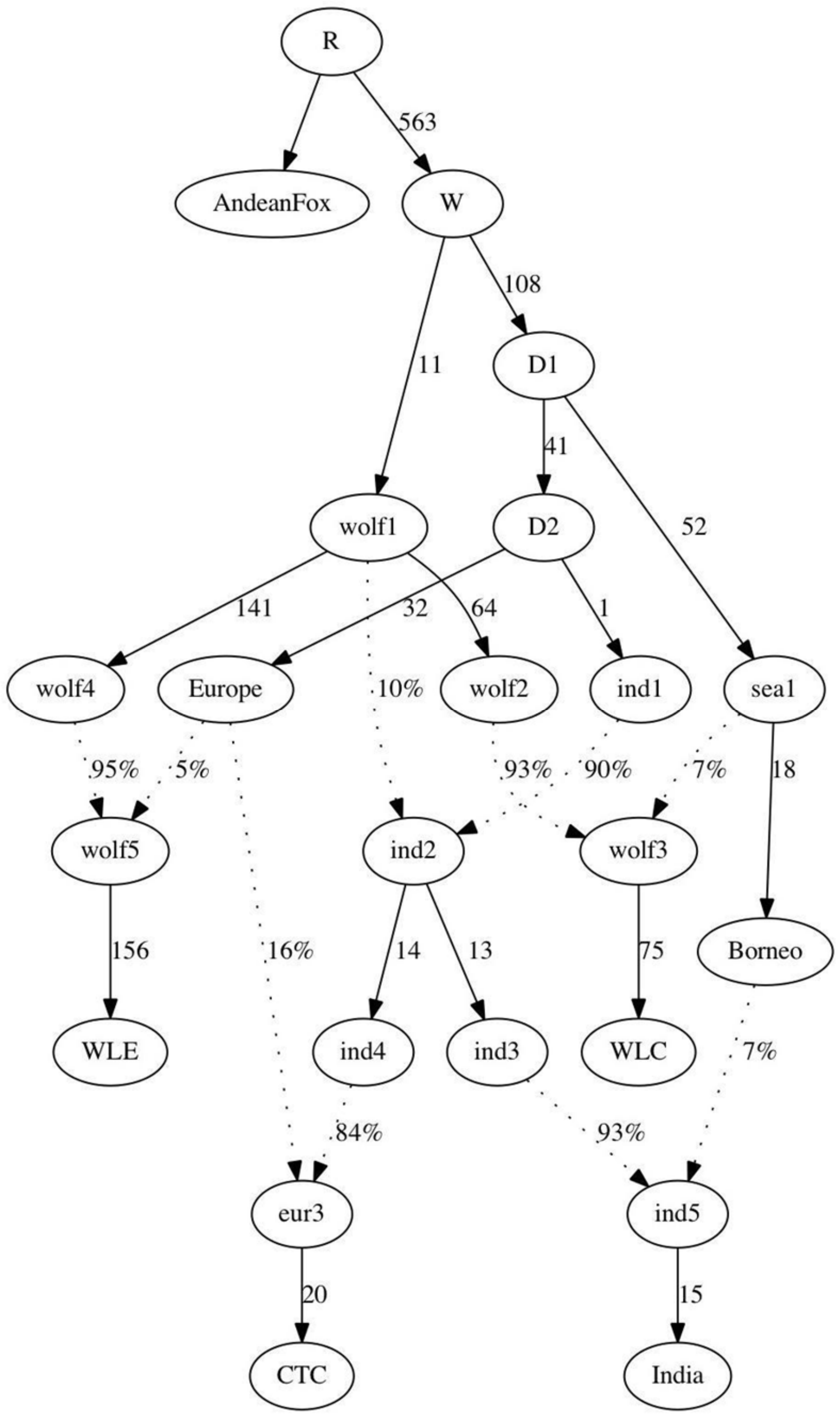

Supplementary Figure 23: Admixture graph with CTC. Model incorporates CTC, modern village dogs and wolves

Supplementary Figure 24

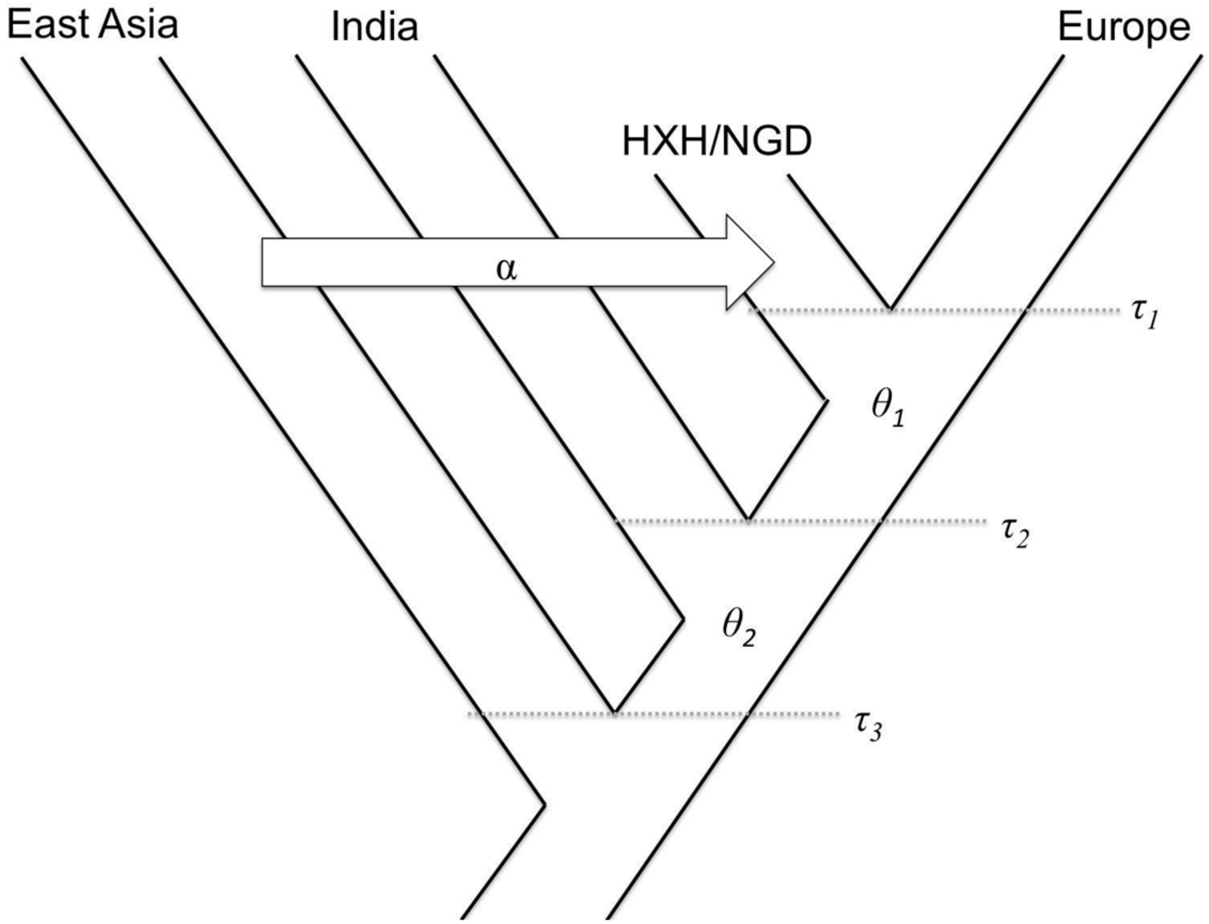

Supplementary Figure 24: Illustration of the tree structure used for estimating HXH/Europe divergence time.

Supplementary Figure 25

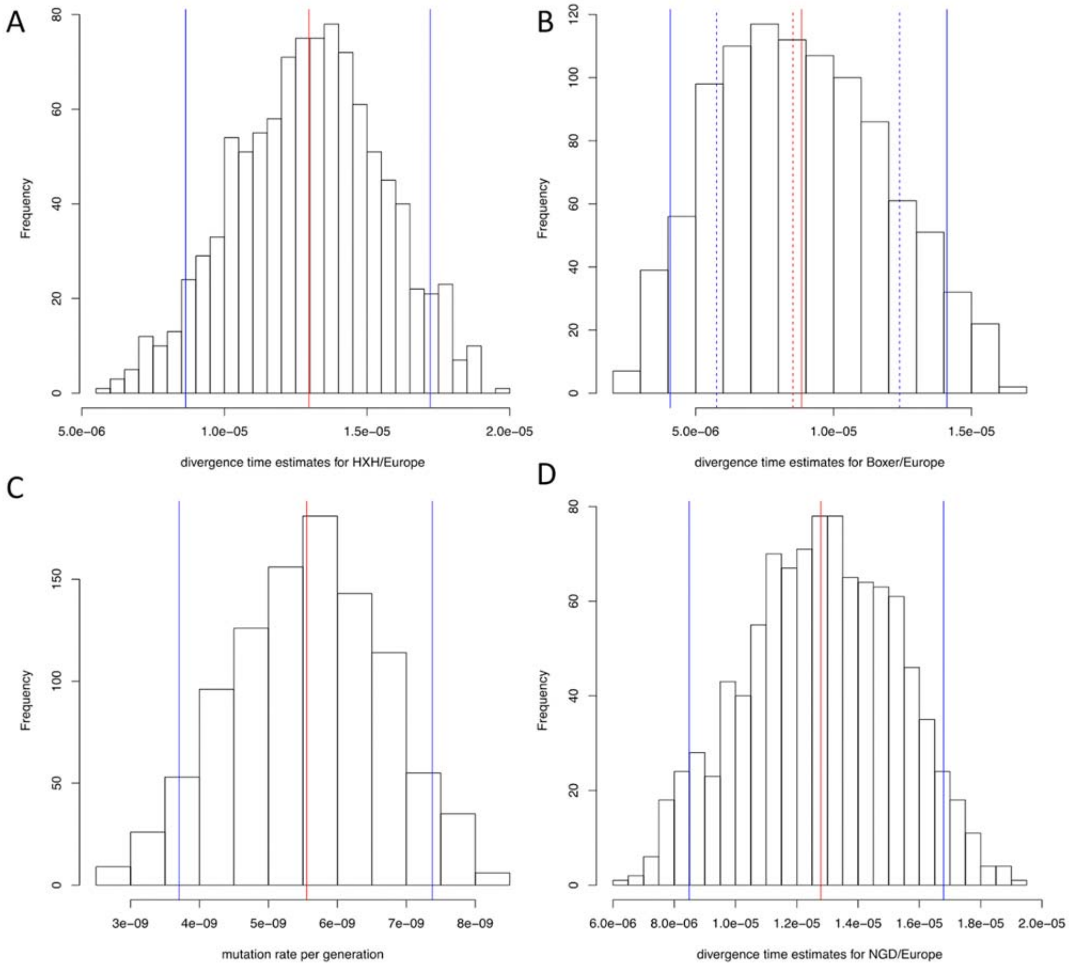

Supplementary Figure 25: Results for numerical analysis. Red lines indicate mean value and blue as confidence intervals. **A)** Inferred divergence time for HXH/Europe. **B)** Inferred divergence time for boxer/Europe using this method compared to G-PhoCS estimates. dashed lines are the G-PhoCS estimates. **C)** Inferred upper boundaries for mutation rate when assuming the divergence time between HXH/Europe must be later than 7,000 years ago. **D)** Inferred divergence time for NGD/Europe.

Supplementary Figure 26

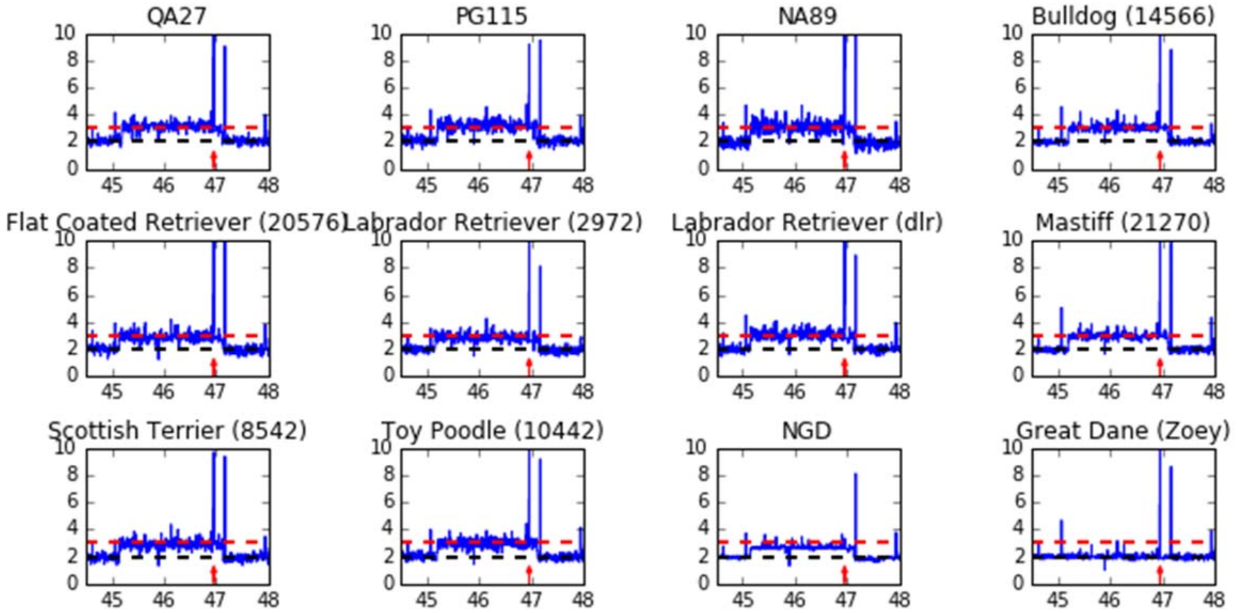

Supplementary Figure 26: Evidence of a large segmental duplication encompassing the *AMY2B* locus. For each sample, estimated copy number (y-axis) is plotted in 3kb windows (blue line) along chromosome 6 (x-axis, in Mb). The dashed black line corresponds to a diploid copy number of 2 while the dashed red line corresponds to a diploid copy number of 3. The red arrow indicates the position of the the *AMY2B* locus. Eleven of the samples contain the extended duplication at this locus, whereas the Great Dane (Zoey) contains greatly increased *AMY2B* copy-number but lacks the segmental duplication. A single window distal to the duplication shows a high level of copy-number variation among all samples.

Supplementary Figure 27

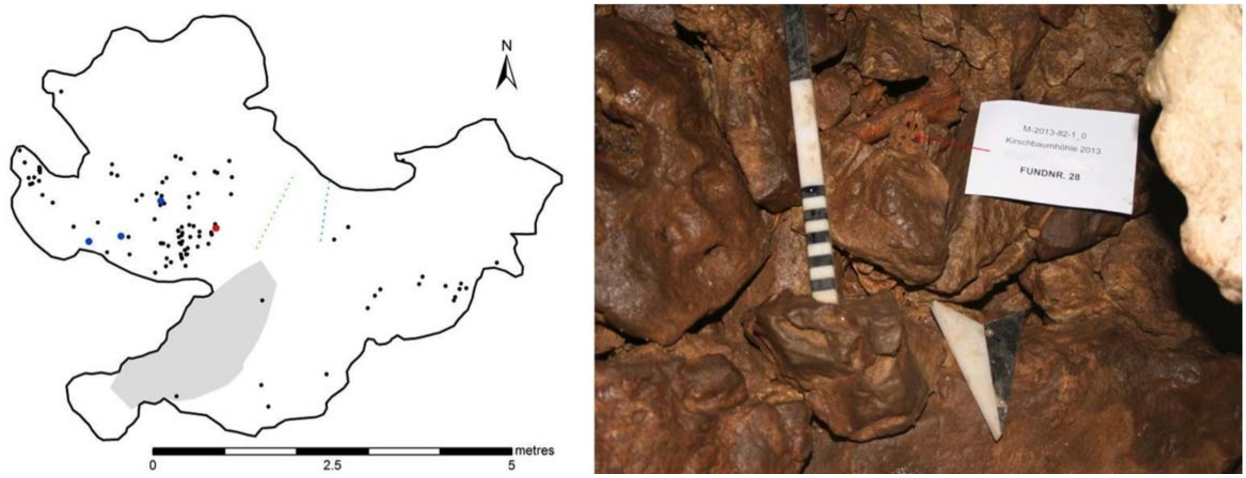

Supplementary Figure 27: *Left*: Ground plan of the "Kirschbaumhöhle" with the Bone Chamber in the western part of the cave. Small black dots are bone findings, red large dot is the finding position of CTC dog cranium, blue large dots are human bones (two skulls and one femur) of the same period (End Neolithic between 2,900 and 2,630 BCE cal.). Dashed lines are the present entrances into the Bone Chamber (green) and into the Sinter Chamber (blue). *Right*: *In situ* picture of CTC dog cranium in the cave (red arrow) overlaid by a limestone.

Supplementary Figure 28

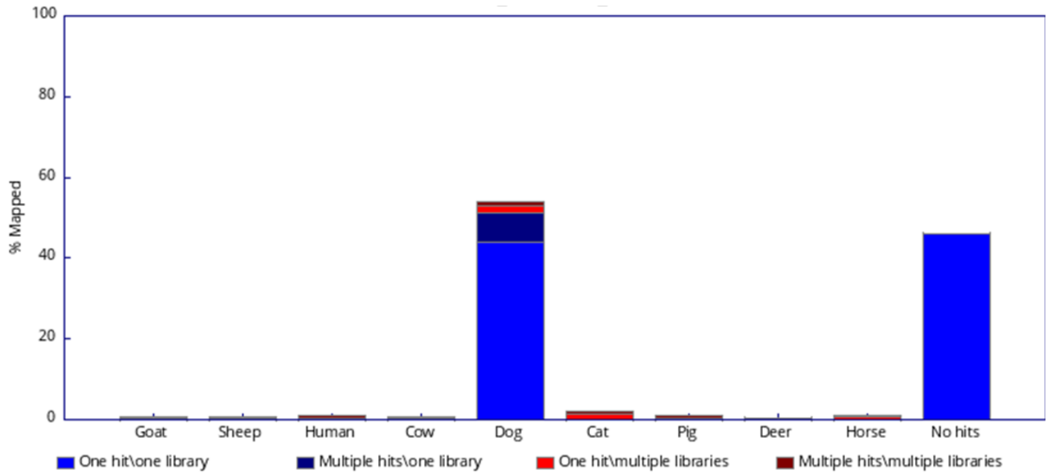

Supplementary Figure 28: `Fastq` screen output of HXH

Supplementary Figure 29

A

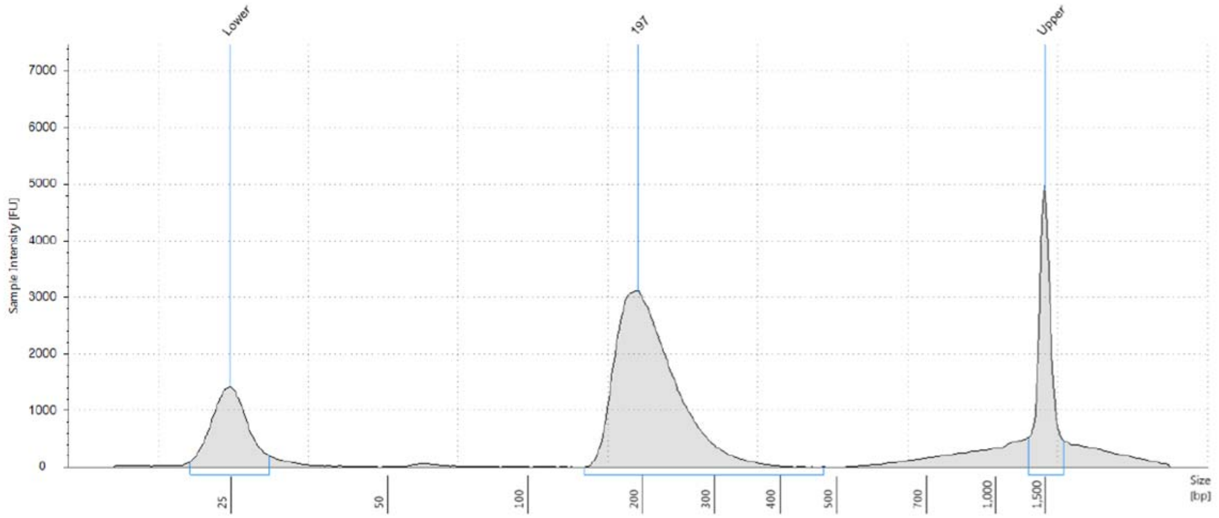

B

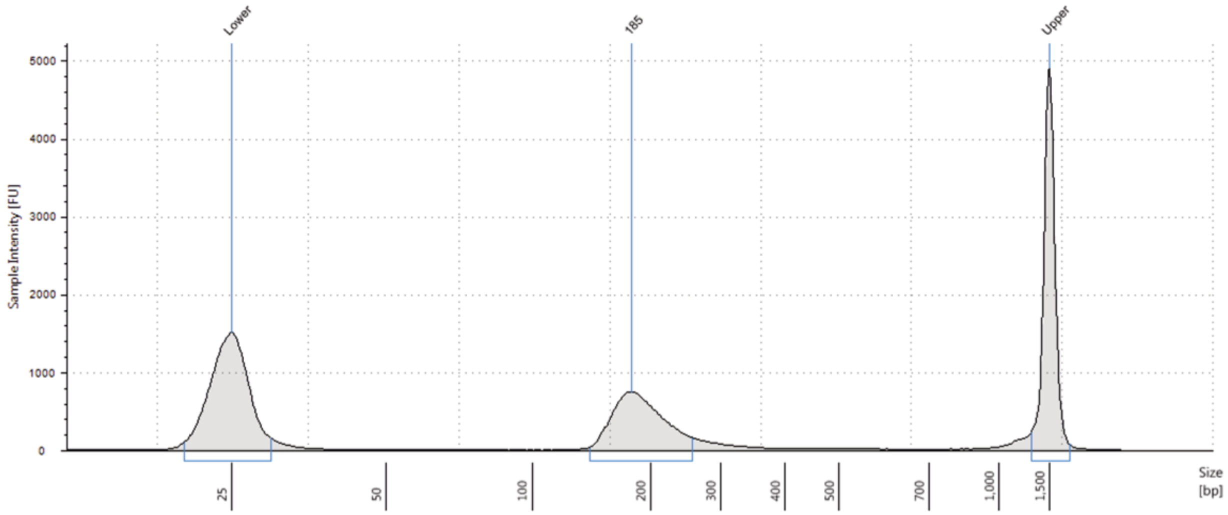

Supplementary Figure 29: Tapestation traces of sample Hxh10a (HXH). **A)** PCR used for MiSeq screening, **B)** Pool of 19 PCRs used for HiSeq sequencing.

Supplementary Figure 30

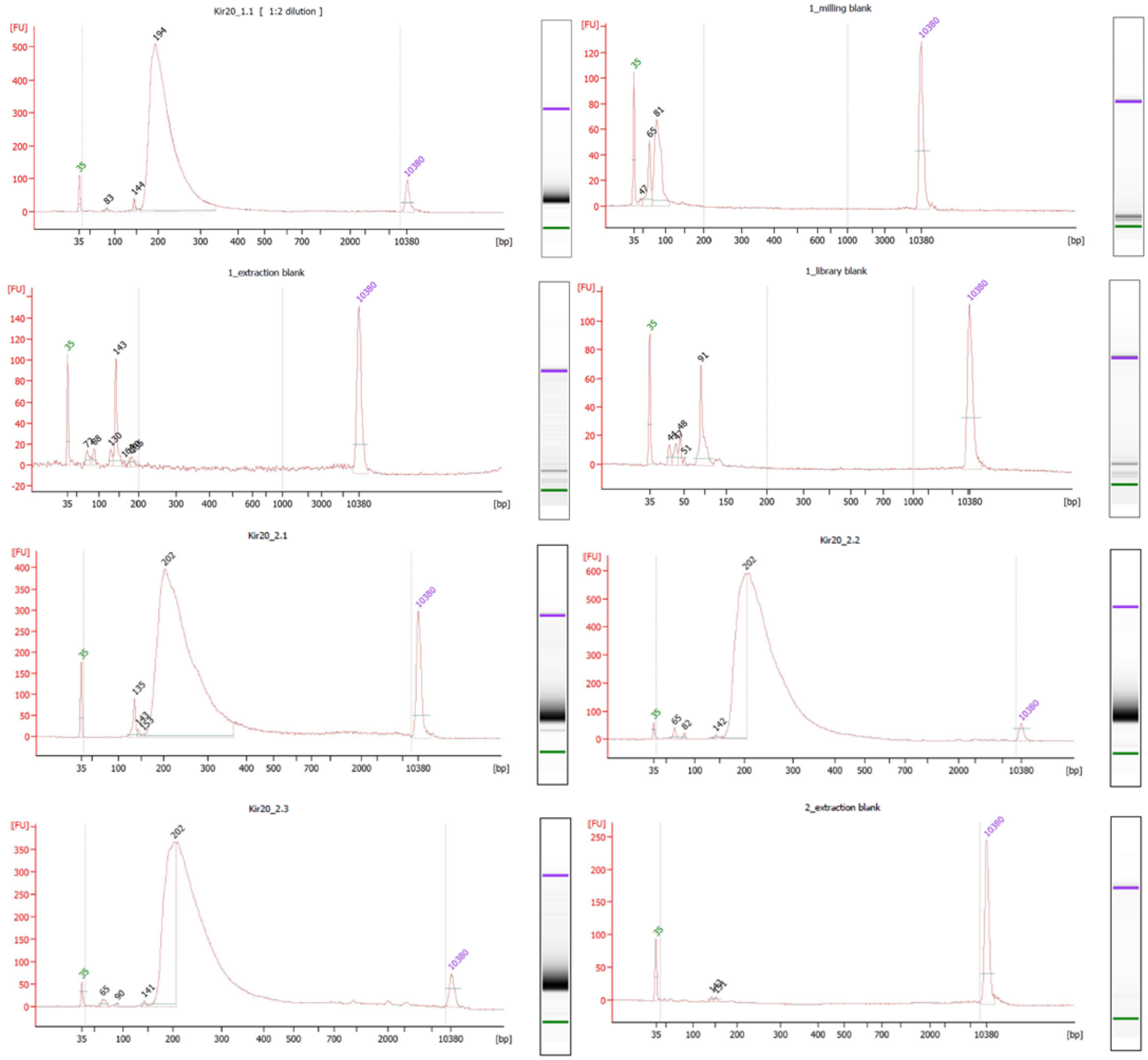

Supplementary Figure 30: Bioanalyzer measurements. Measurements for all sequenced libraries and corresponding blank controls of sample Kir20 (CTC). Labelling according to Supplementary Table 22.

Supplementary Figure 31

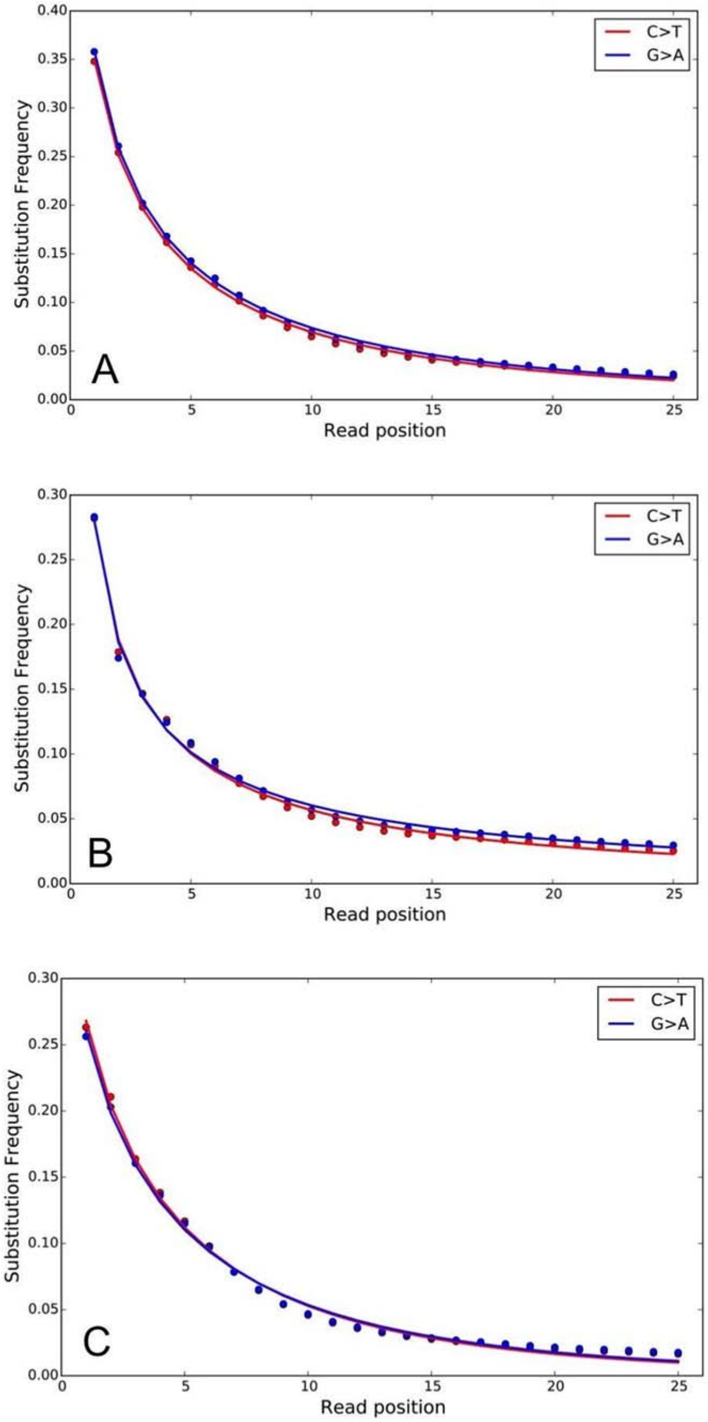

Supplementary Figure 31: Weibull function fit of the frequency of the base substitution along the read. A) HXH **B)** CTC and **C)** NGD

Supplementary Figure 32

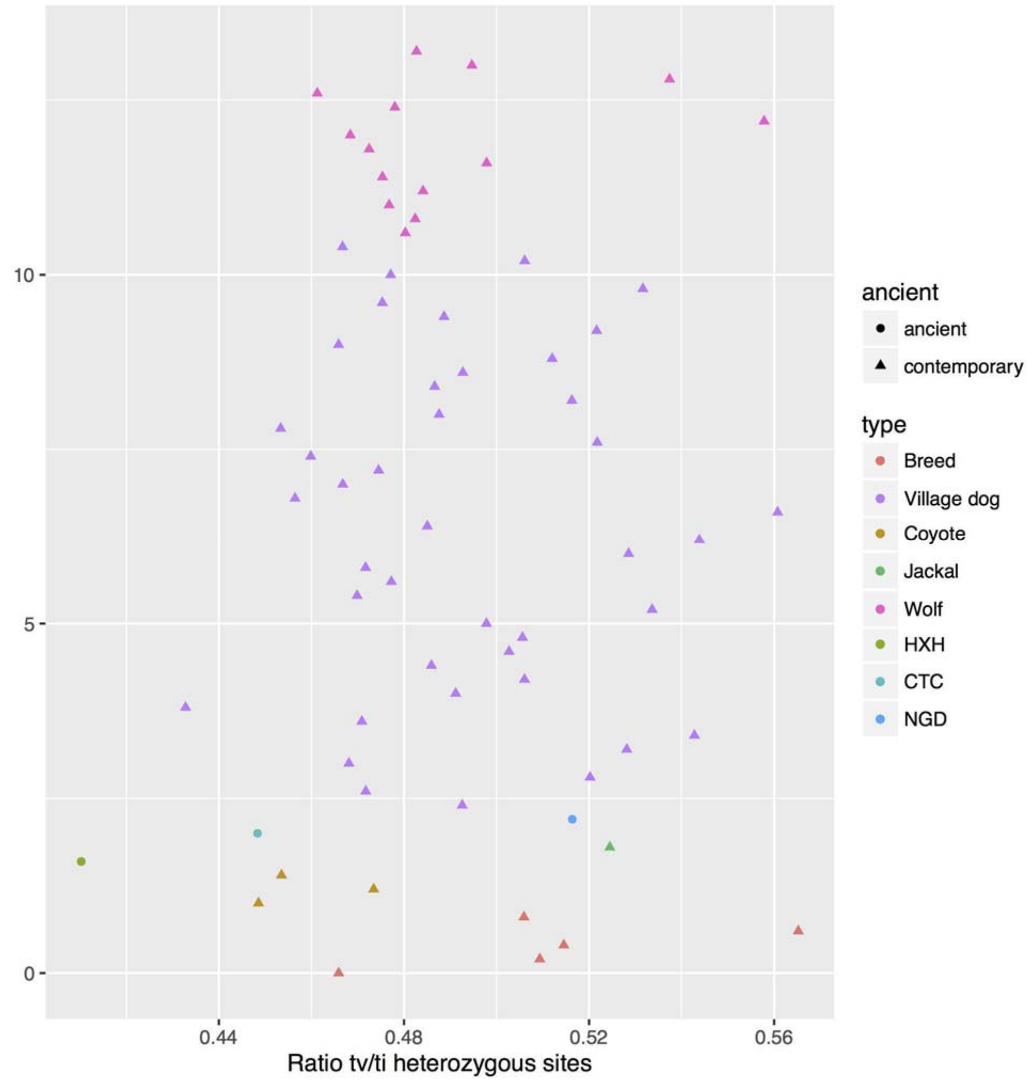

Supplementary Figure 32: Transversion/Transition ratio. Heterozygous sites for modern and ancient canid genomes. Samples are sorted and arranged along the y-axis.

Supplementary Figure 33

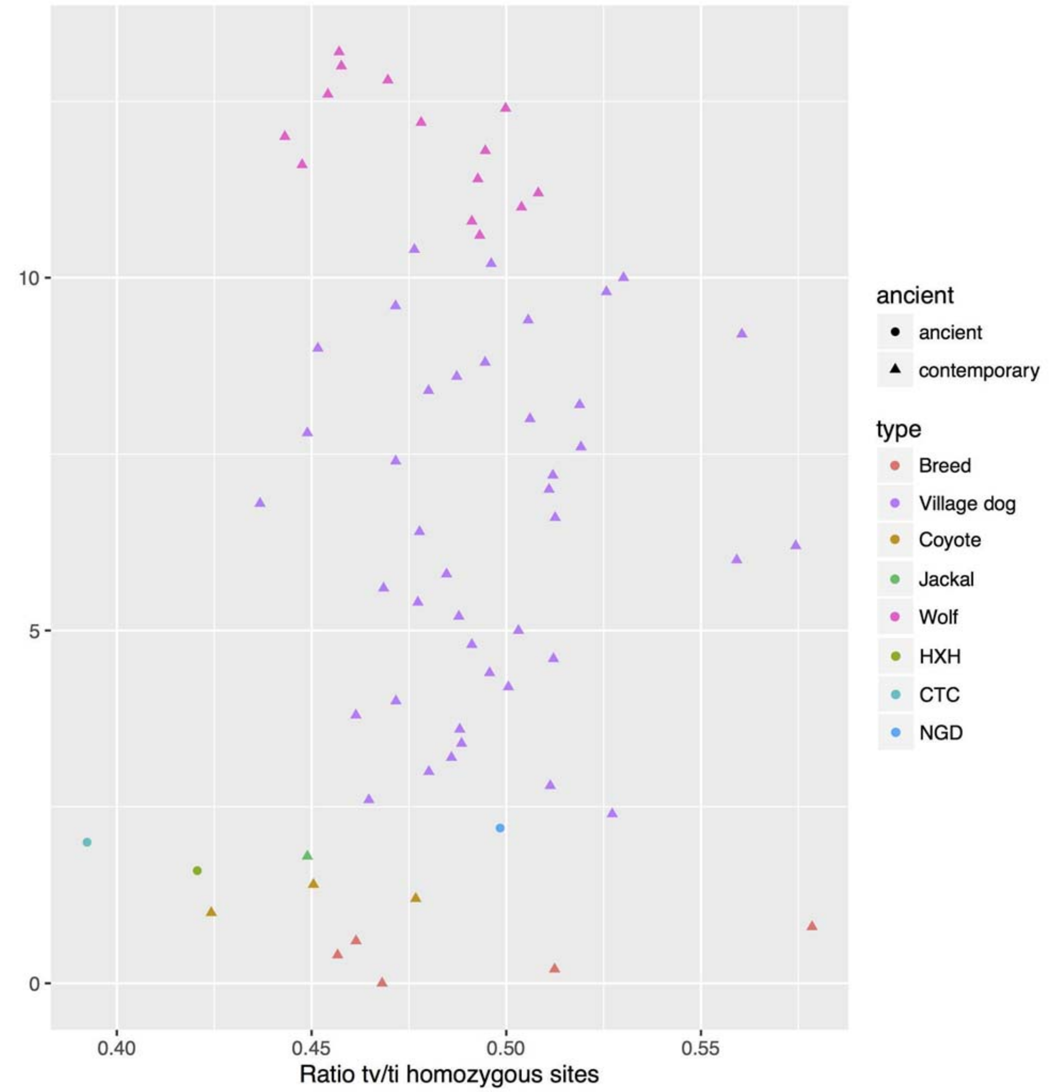

Supplementary Figure 33: Transversion/Transition ratio. Homozygous sites for modern and ancient canid genomes. Samples are sorted and arranged along the y-axis.

Supplementary Figure 34

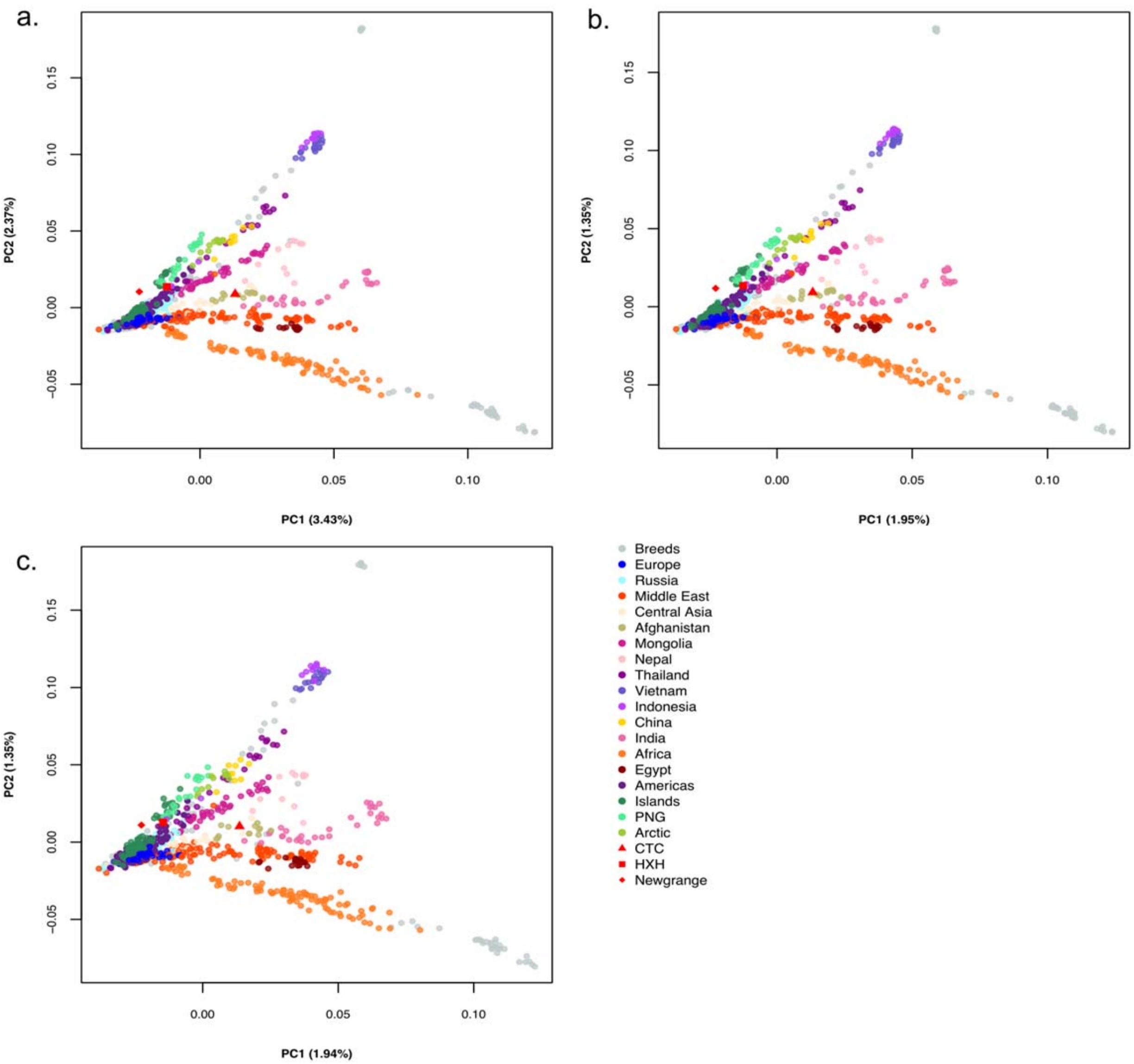

Supplementary Figure 34: PCA Analysis. Comparison of SNP array data-based PCAs based on a) diploid, b) pseudo-haploid, and c) pseudo-haploid data with C<>T and A<>G sites removed. PC space defined by village dogs, with breed dogs, CTC, HXH, and NGD projected onto the PC space. Village dogs are colored according to geography; breed dogs are light gray; ancient samples are red.

Supplementary Figure 35

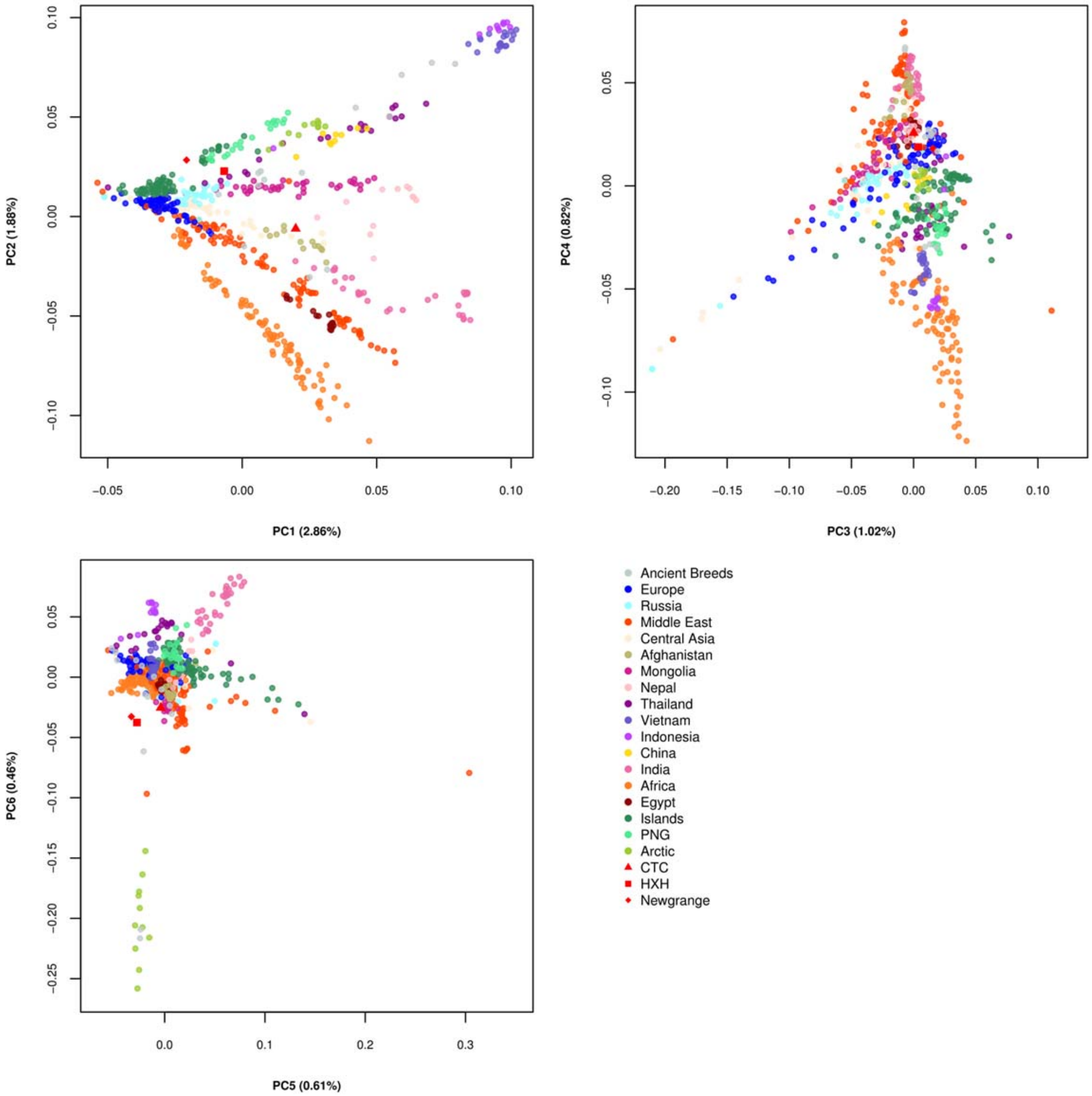

Supplementary Figure 35. PCA Analysis. PCA of village dogs, with breed dogs, CTC, HXH, and Newgrange dog (NGD) projected onto the PC space using SNP array data. Village dogs are colored according to geography; breed dogs are light gray; ancient samples are red.

Supplementary Figure 36

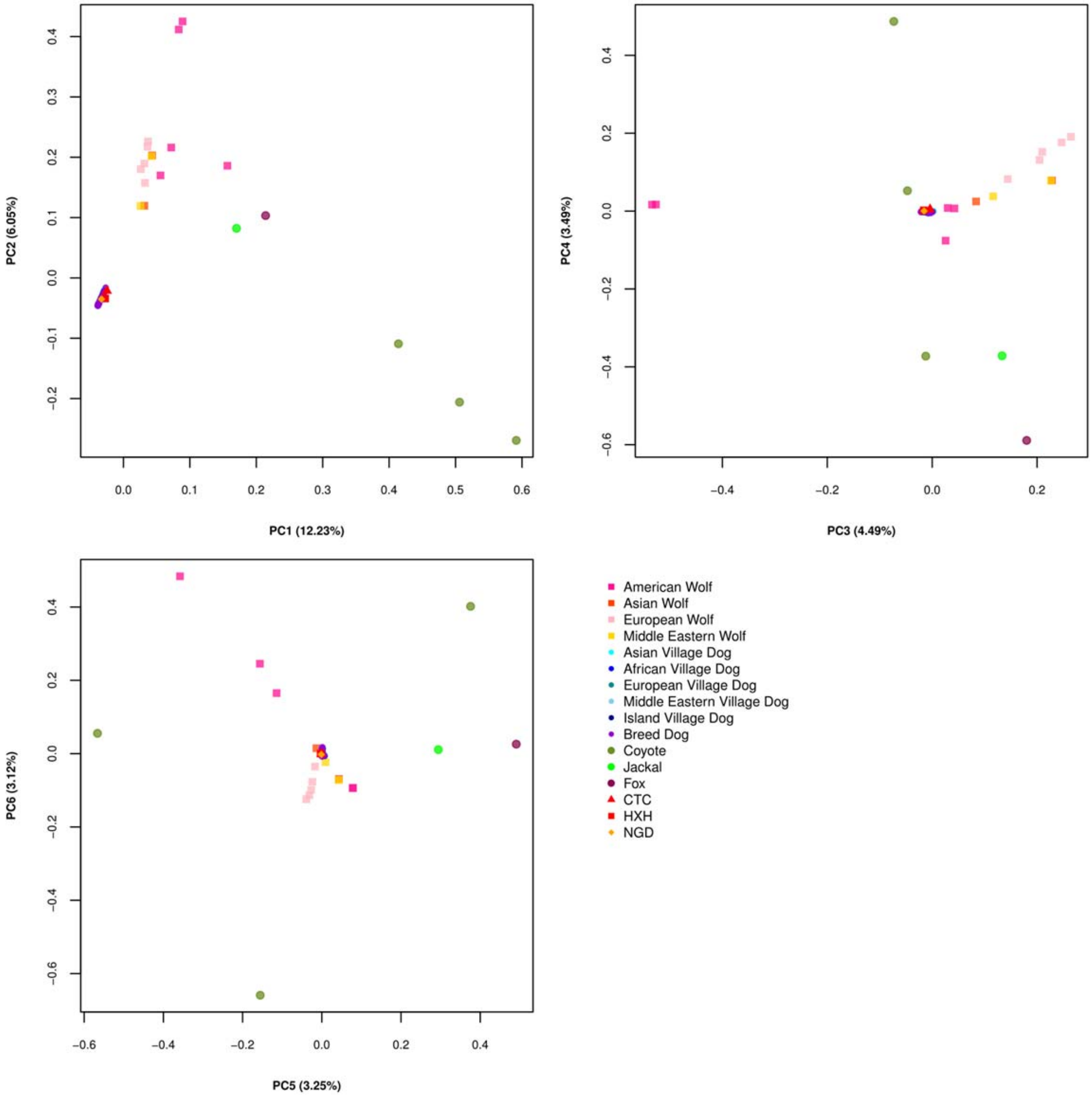

Supplementary Figure 36: PCA analysis. PCA of coyotes, jackal, fox, gray wolves, village dogs, breed dogs, NGD, CTC, and HXH using the genome sequenced Call set 1. PC space was defined by all samples.

Supplementary Figure 37

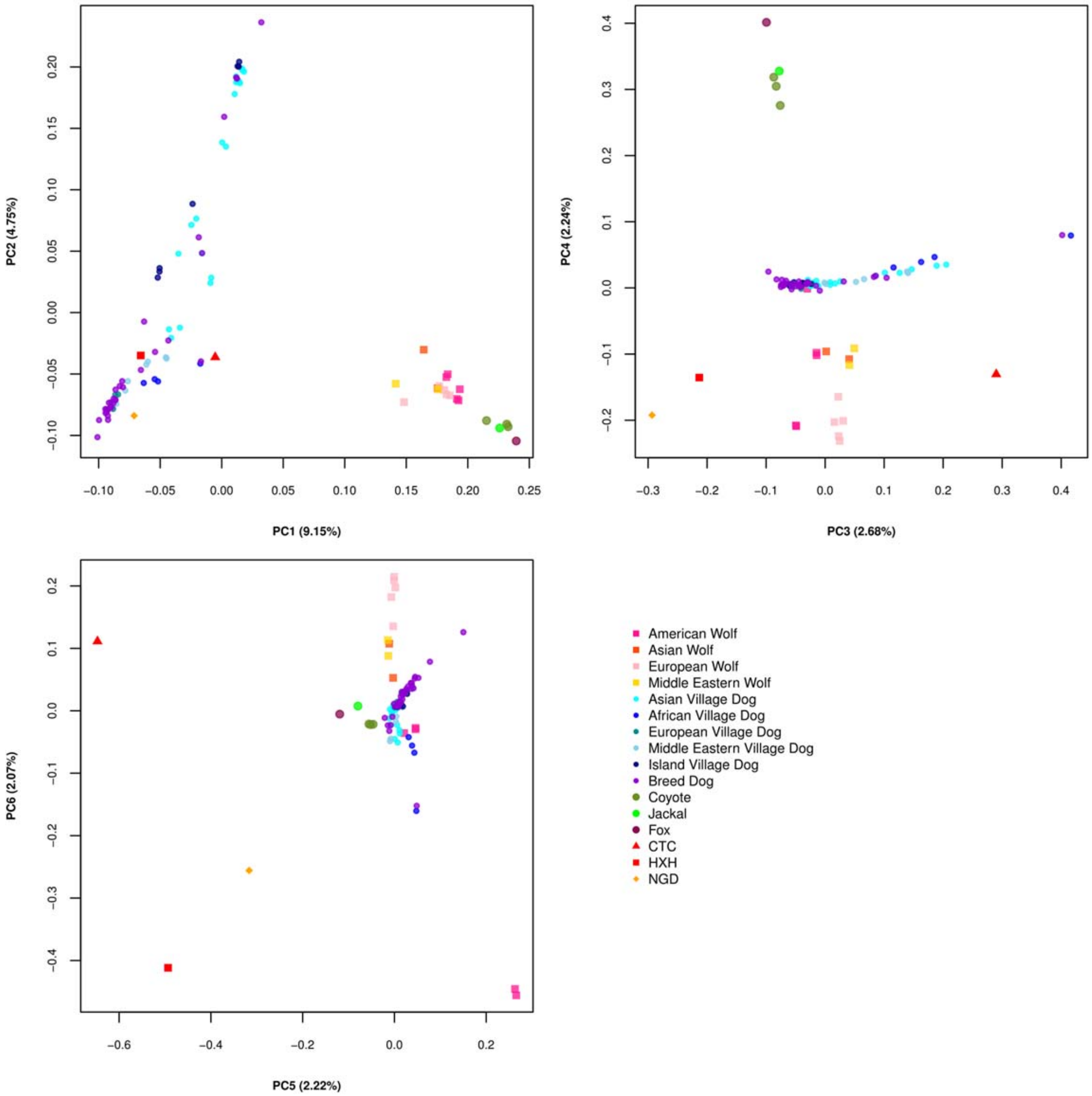

Supplementary Figure 37: PCA analysis. PCA of coyotes, jackal, fox, gray wolves, village dogs, breed dogs, NGD, CTC, and HXH using the genome sequenced Call set 2. PC space was defined by all samples.

Supplementary Figure 38

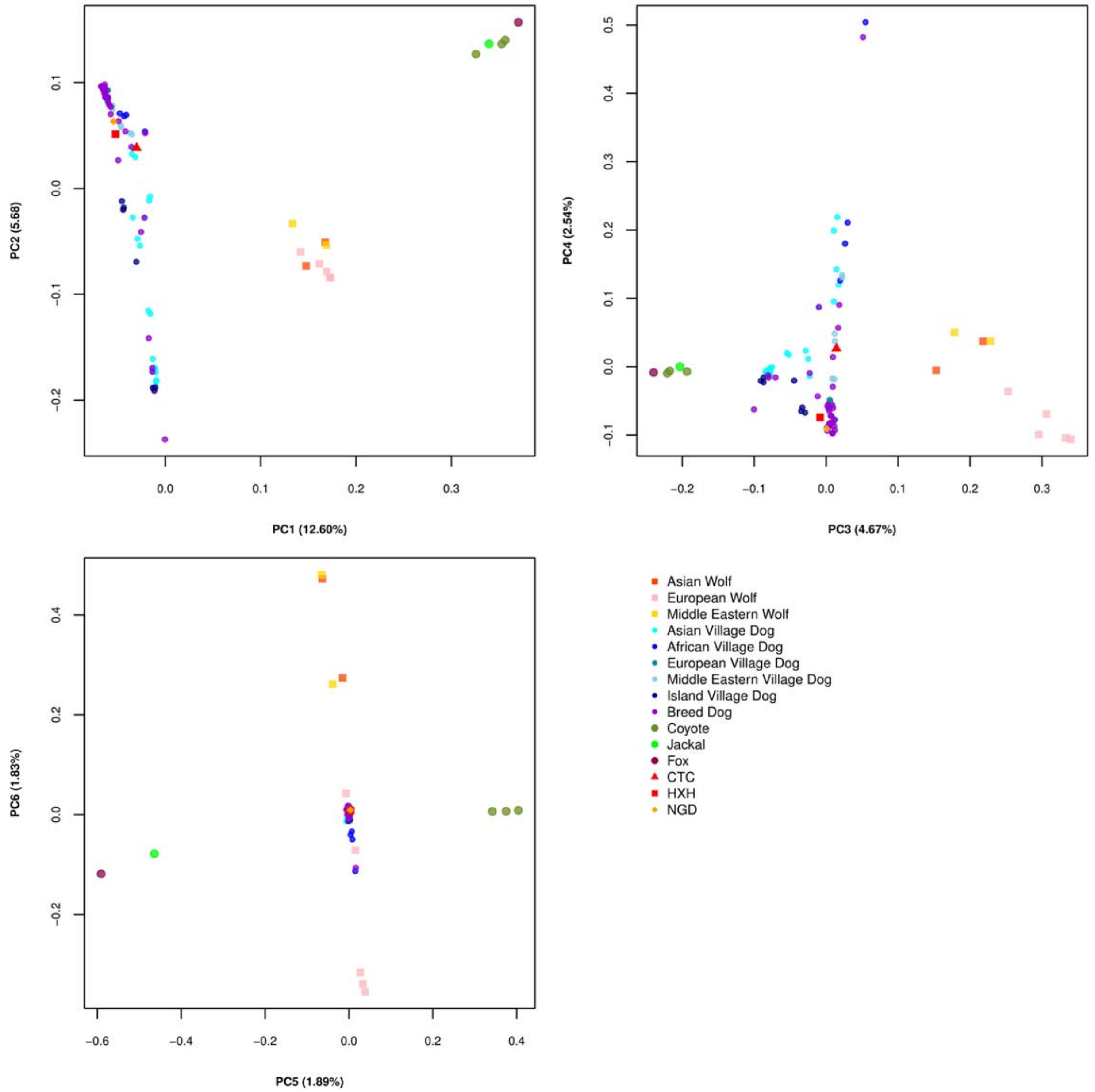

Supplementary Figure 38: PCA analysis. PCA of coyotes, jackal, fox, gray wolves (excluding New World wolves), village dogs, breed dogs,NGD, CTC, and HXH using the genome sequenced Call set 3. PC space was defined by all samples.

Supplementary Figure 39

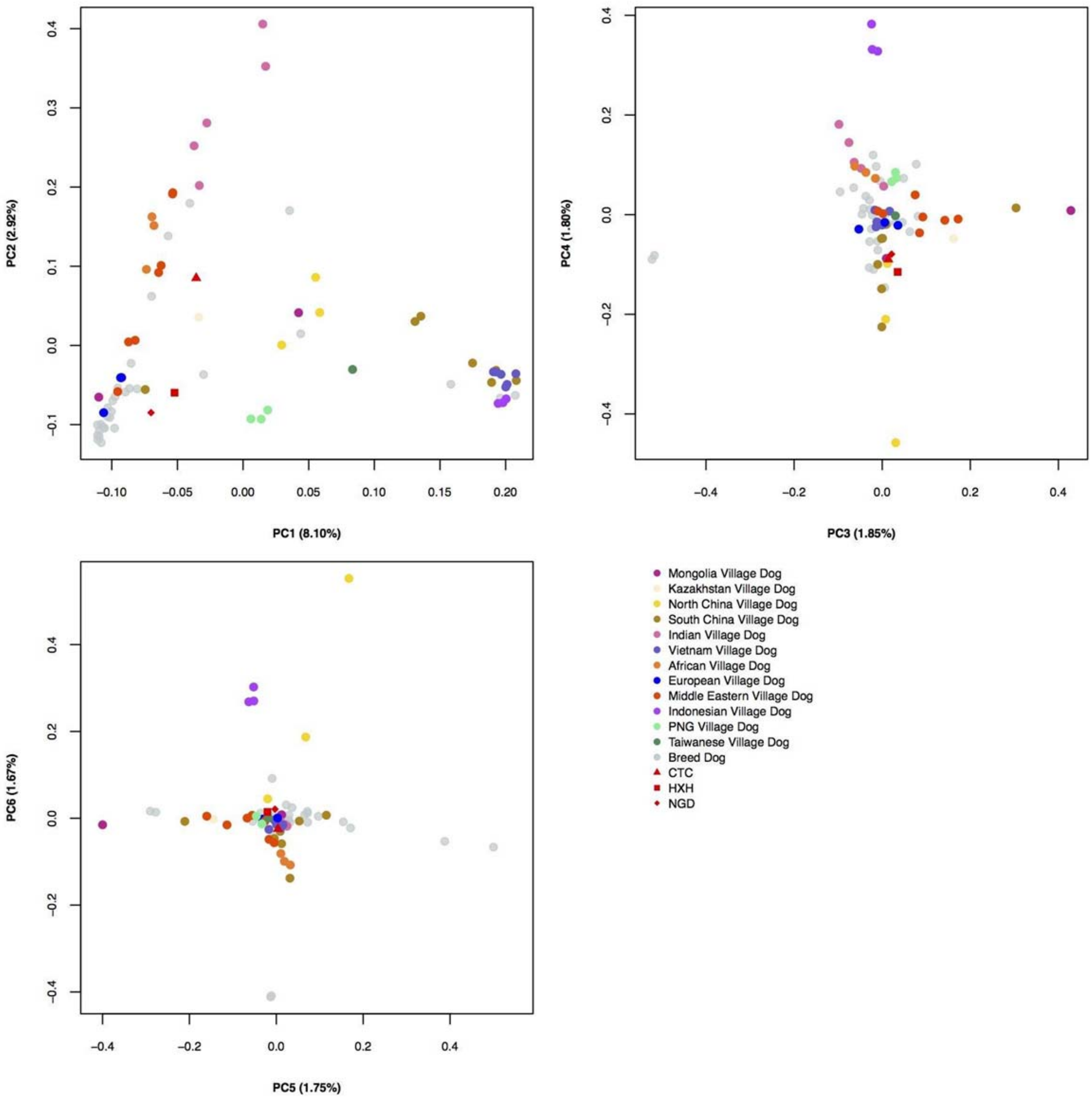

Supplementary Figure 39: PCA analysis. PCA of village dogs, breed dogs, NGD, CTC, and HXH using the genome sequenced Call set 1. PC space was defined by all samples.

Supplementary Figure 40

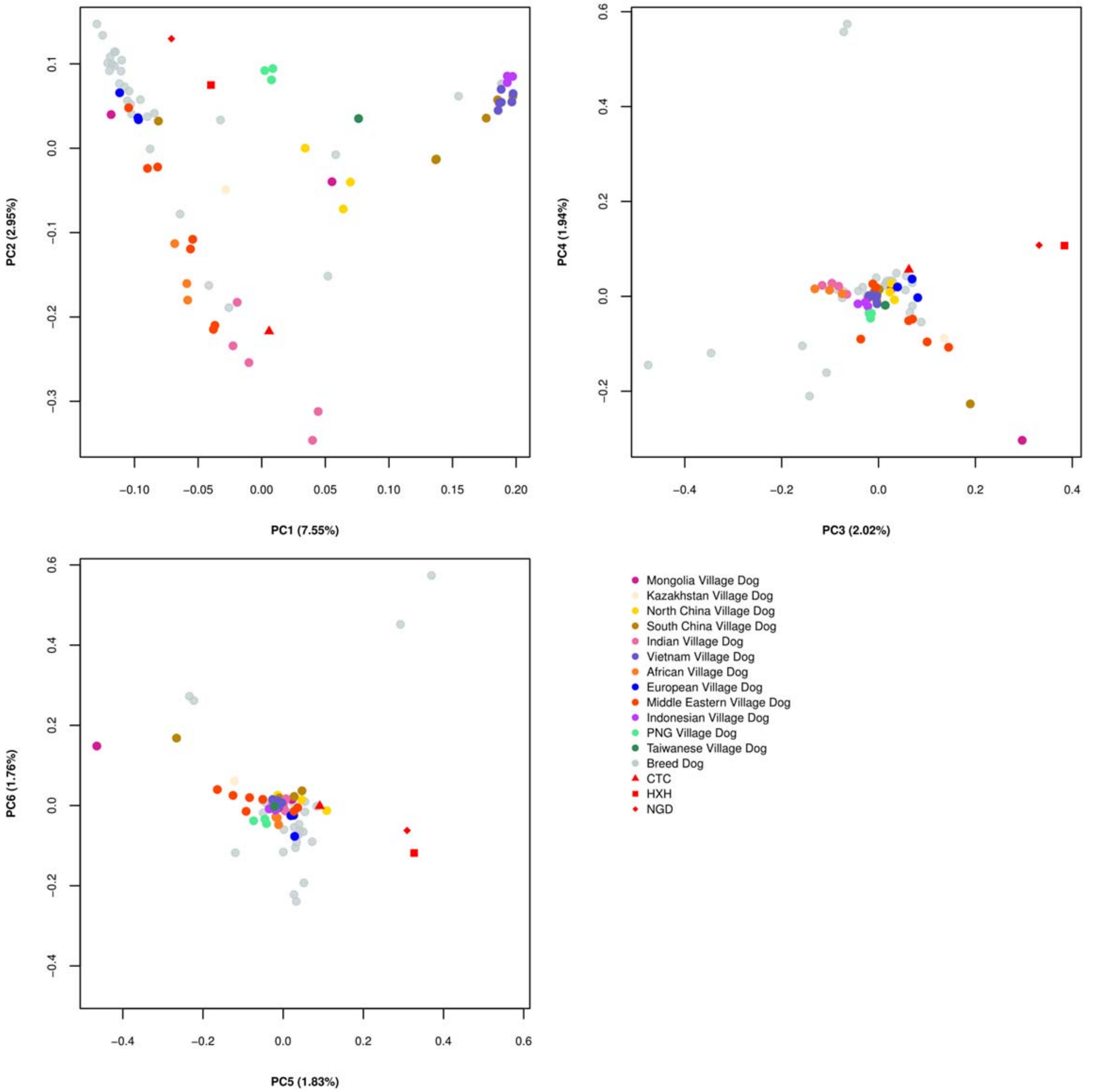

Supplementary Figure 40: PCA analysis. PCA of village dogs, breed dogs, NGD, CTC, and HXH using the genome sequenced Call set 2. PC space was defined by all samples.

Supplementary Figure 41

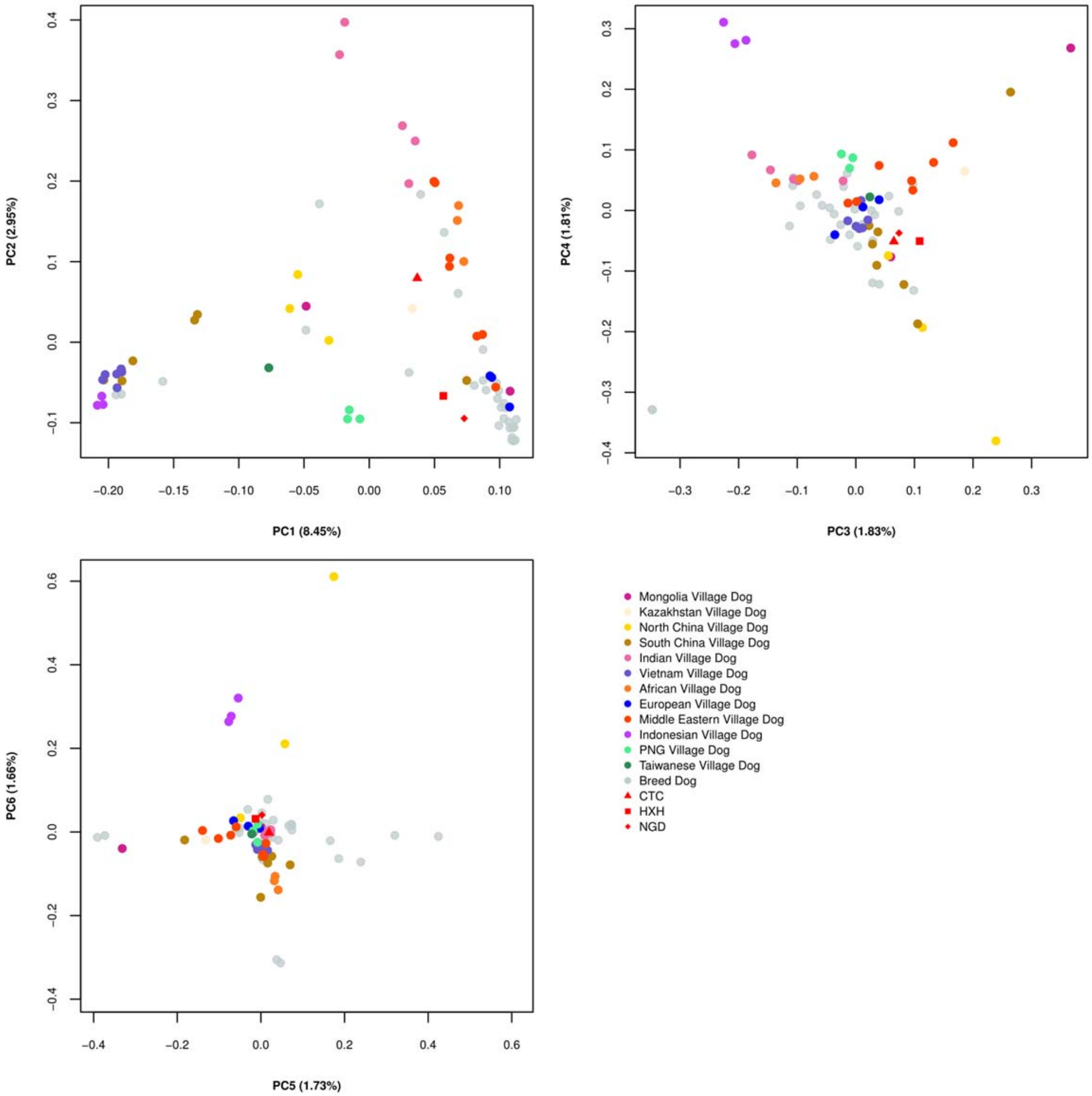

Supplementary Figure 41: PCA analysis. PCA of village dogs, breed dogs, NGD, CTC, and HXH using the genome sequenced Call set 3. PC space was defined by all samples.

Supplementary Figure 42

A) **B)**

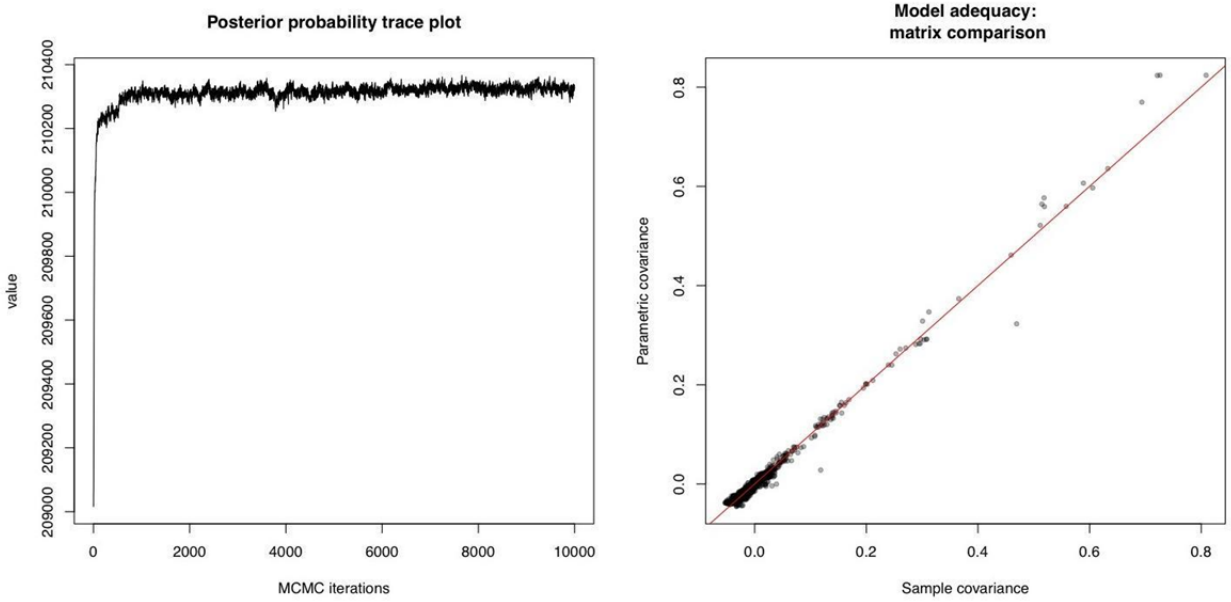

Supplementary Figure 42: Validation of Spacemix run. **A)** Stabilization of the posterior probability during MCMC iterations. **B)** Comparison of the parametric and the sample covariance.

Supplementary Figure 43

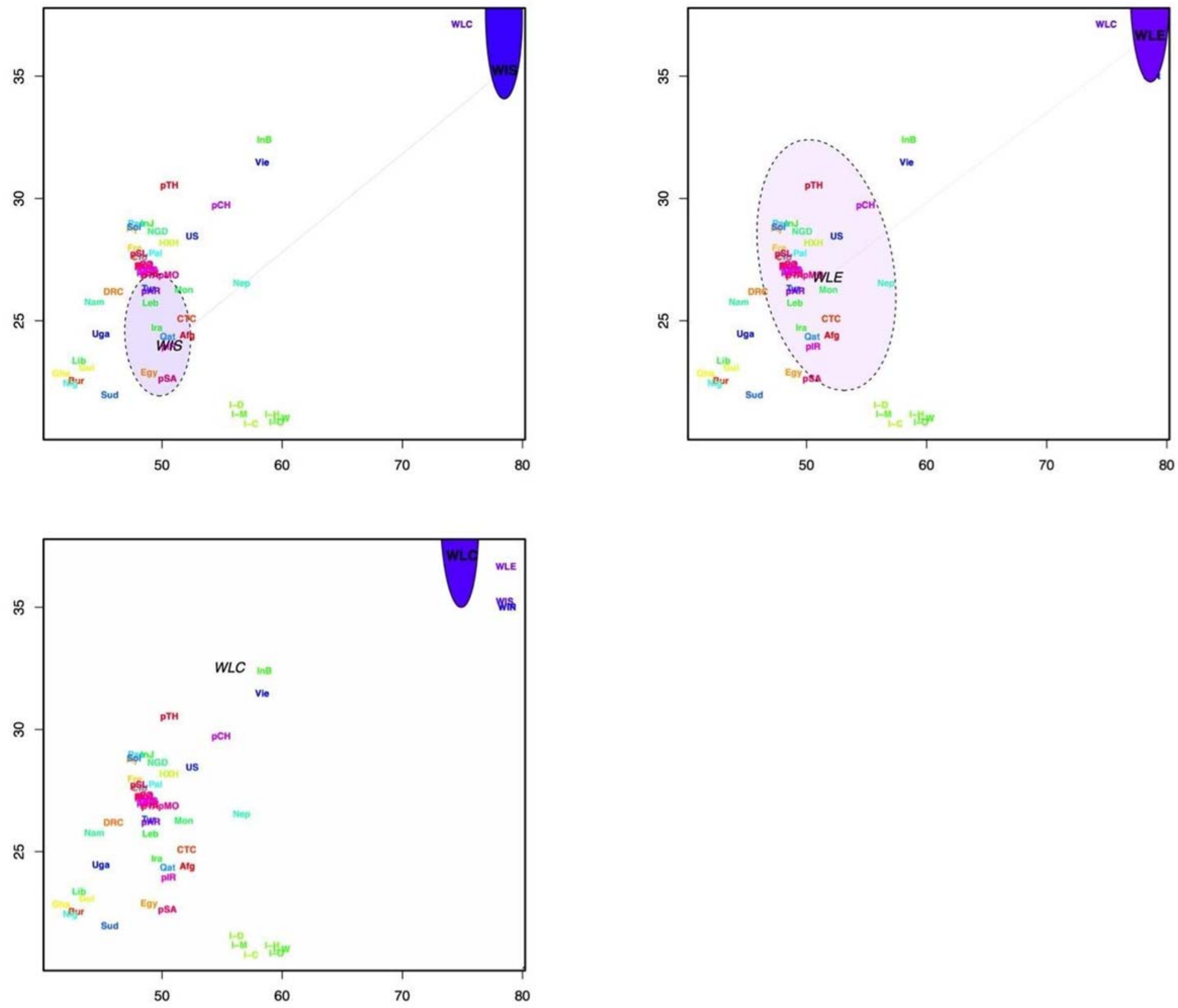

Supplementary Figure 43: Spacemix results. Wolf-dog admixture estimated by SpaceMix

Supplementary Figure 44

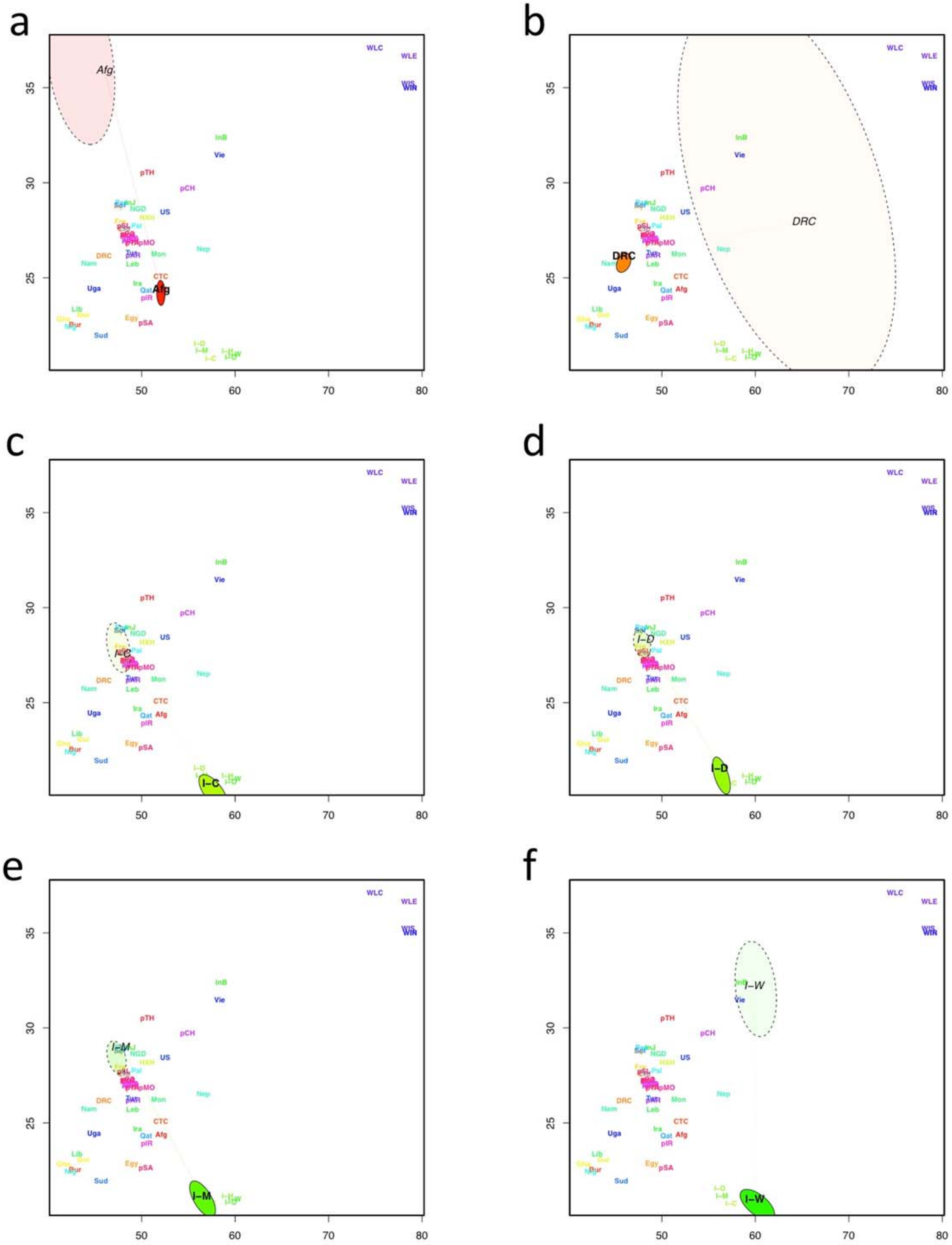

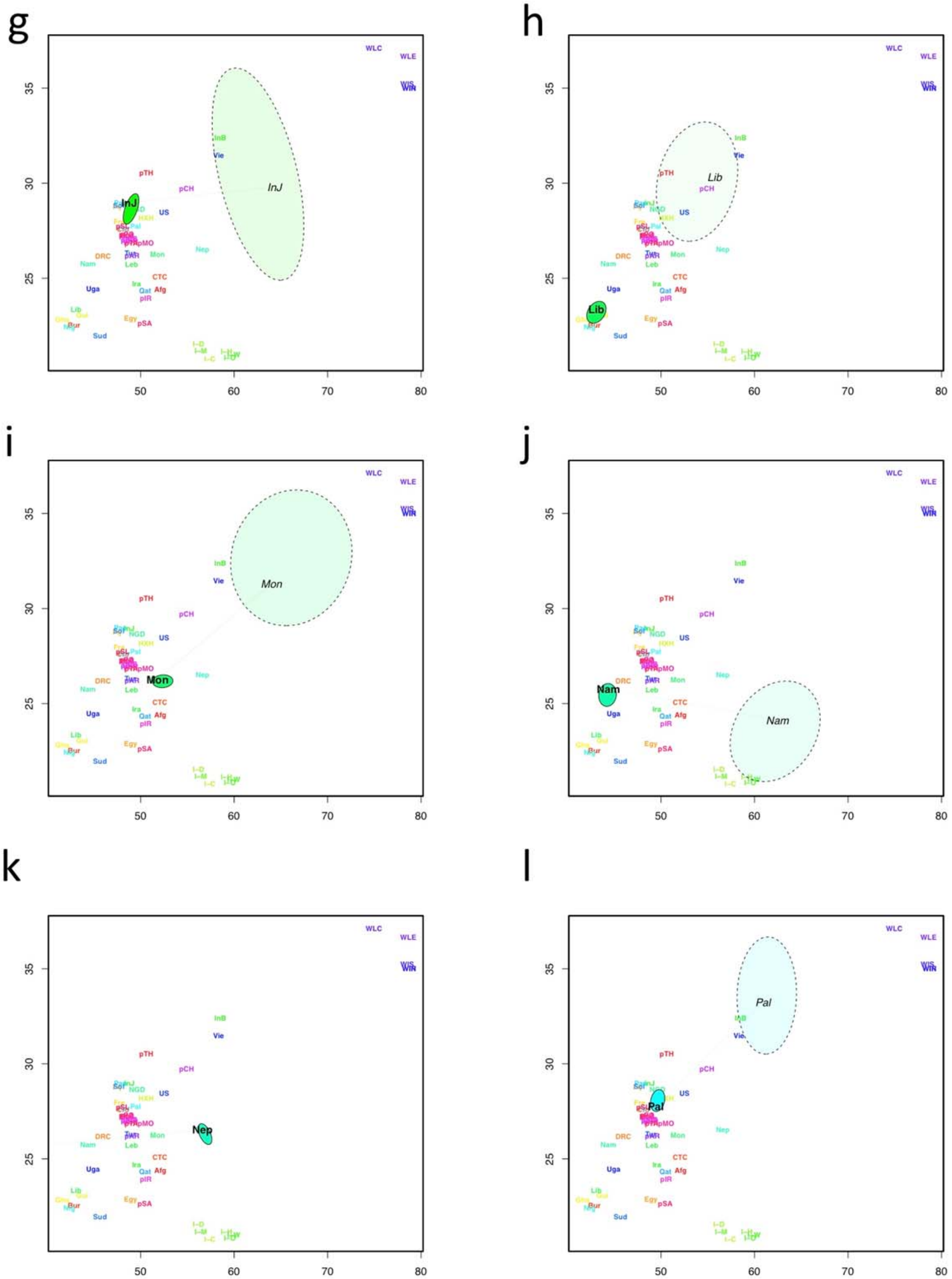
g
h
i
j
k
l
InJ
Lib
Mon
Nam
Nep
Pal
WLC
WLE
WIS
WIN
InB
Vie
pTH
pCH
US
HXH
NGD
Nep
DRC
Nam
Mon
Leb
Uga
CTC
Ira
Qat
Afg
pIR
Lib
Egy
pSA
Bur
Sud
I-D
I-M
I-C
35
30
25
50
60
70
80

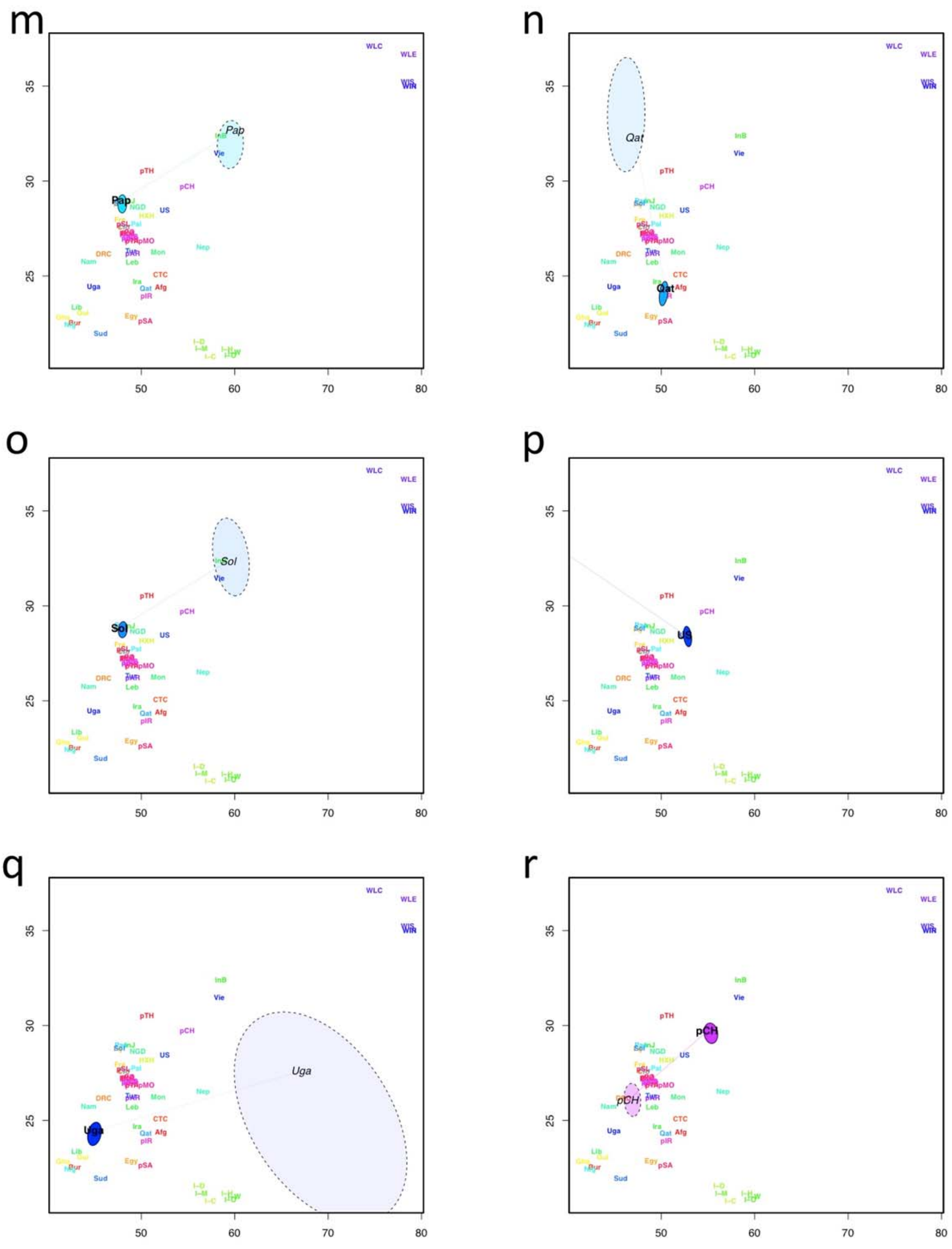
m
n
o
p
q
r
Pap
Qat
Sol
US
Uga
pCH
WLC
WLE
WIS
WIN
InB
Vie
pTH
NGD
HXH
Pal
Nep
Mon
DRC
Nam
Leb
CTC
Ira
Afg
pIR
Lib
Bur
Sud
Egy
pSA
pMO
25
30
35
50
60
70
80

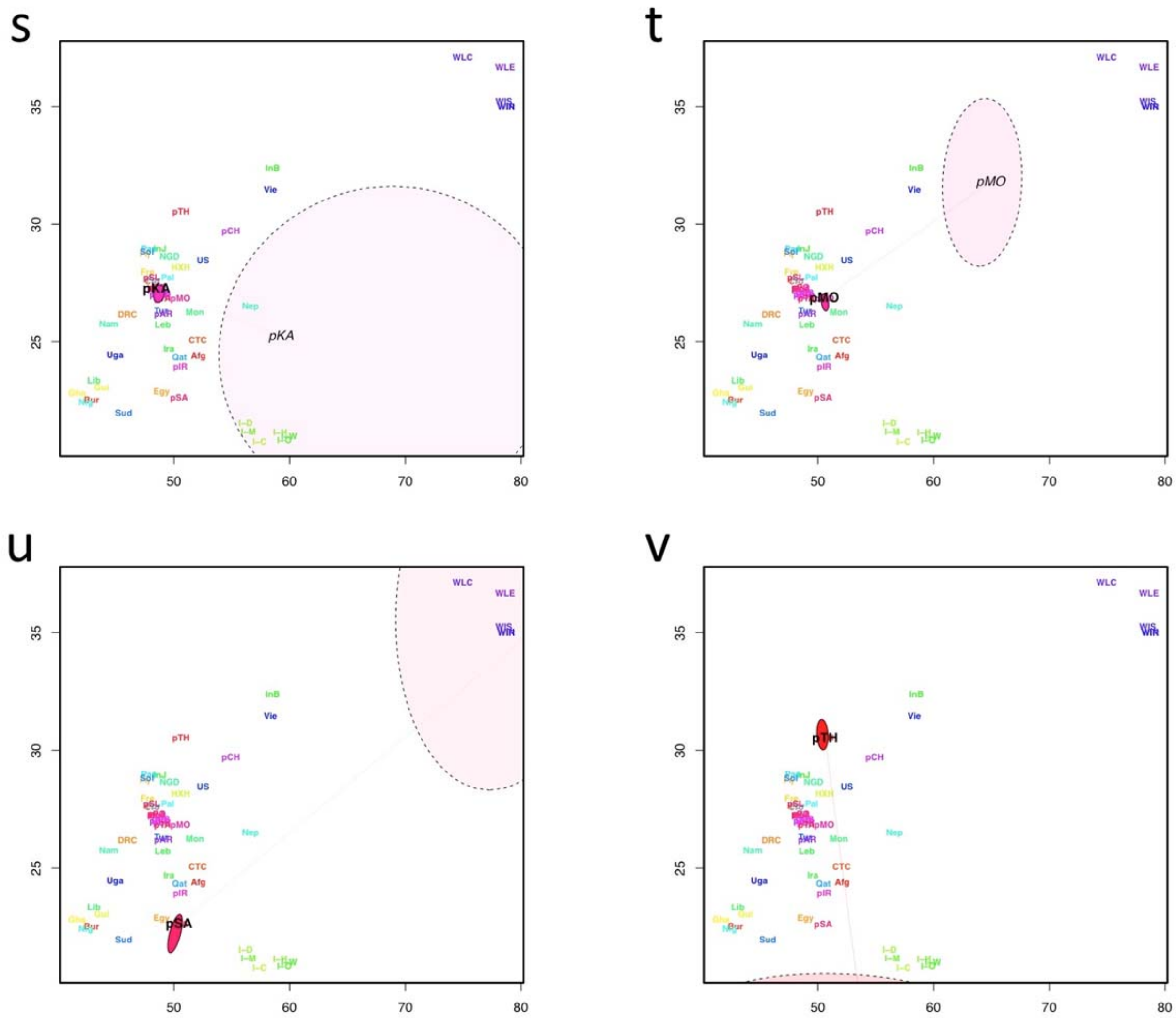

Supplementary Figure 44: SpaceMix results. Modern dog-modern dog admixture estimated by SpaceMix

Supplementary Figure 45

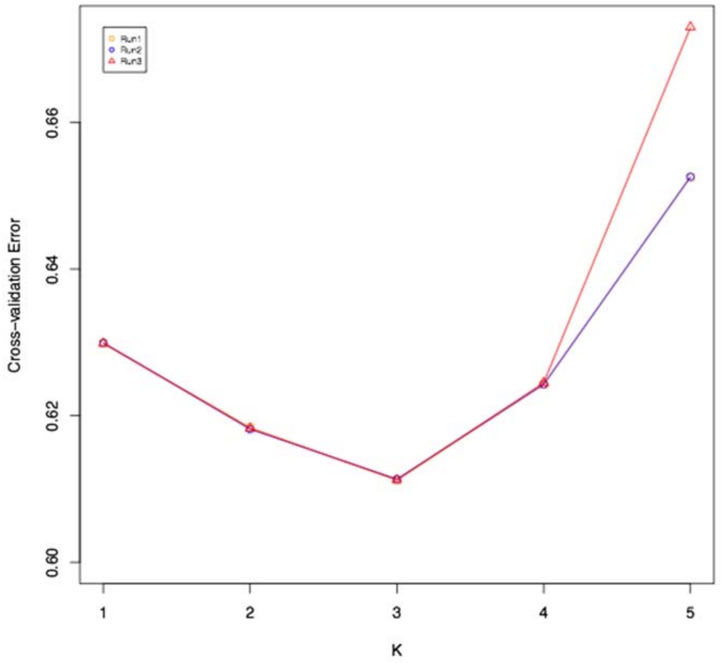

Supplementary Figure 45: Admixture results. Cross-validation errors for *K* values 1 through 5 for three separate runs of `ADMIXTURE` using the SNP array data set

Supplementary Figure 46

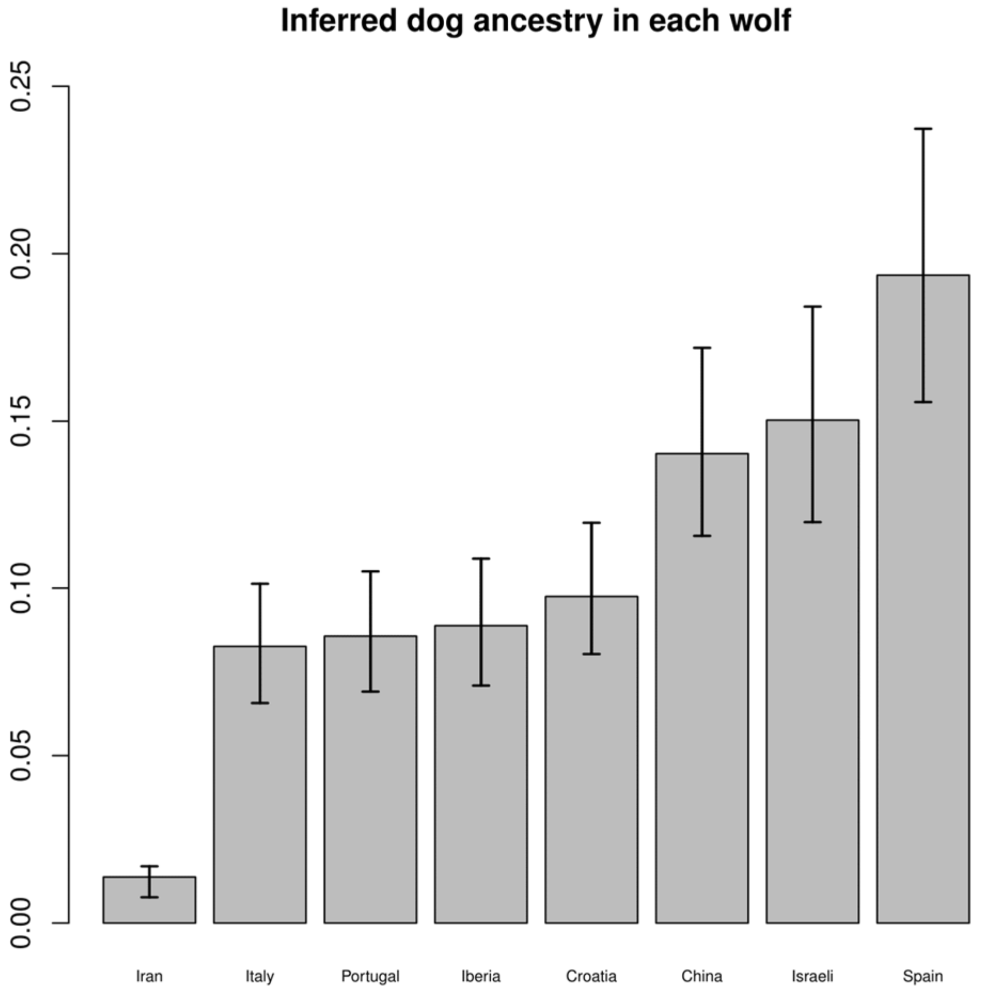

Supplementary Figure 46. The inferred proportion of dog ancestry in each wolf. The boxplot shows minimum, mean and maximum.

Supplementary Figure 47

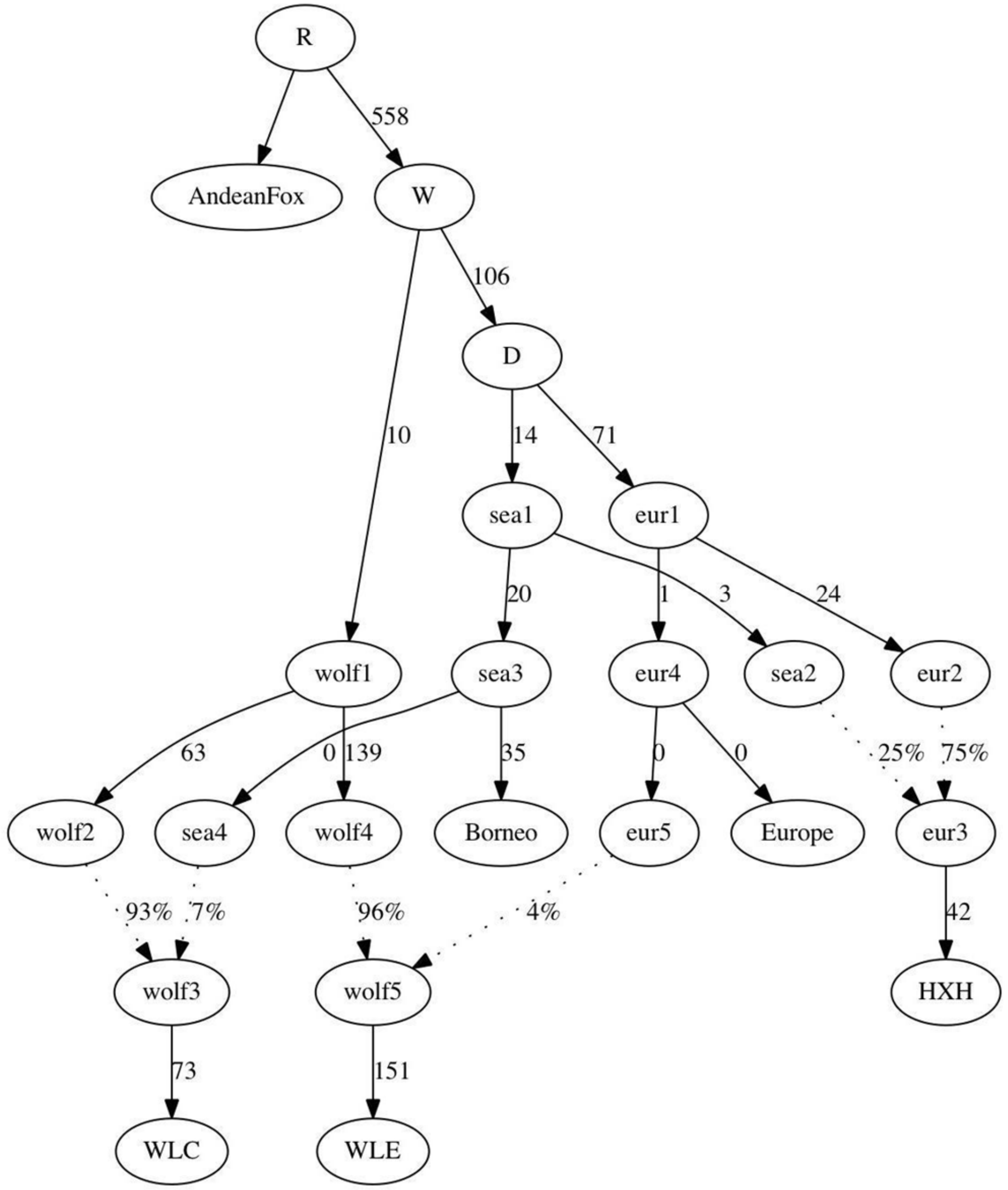

Supplementary Figure 47: Best fit admixture graph for HXH (NGD not shown but is similar) when including modern European and Southeast Asian village dogs and wolves.

Supplementary Figure 48

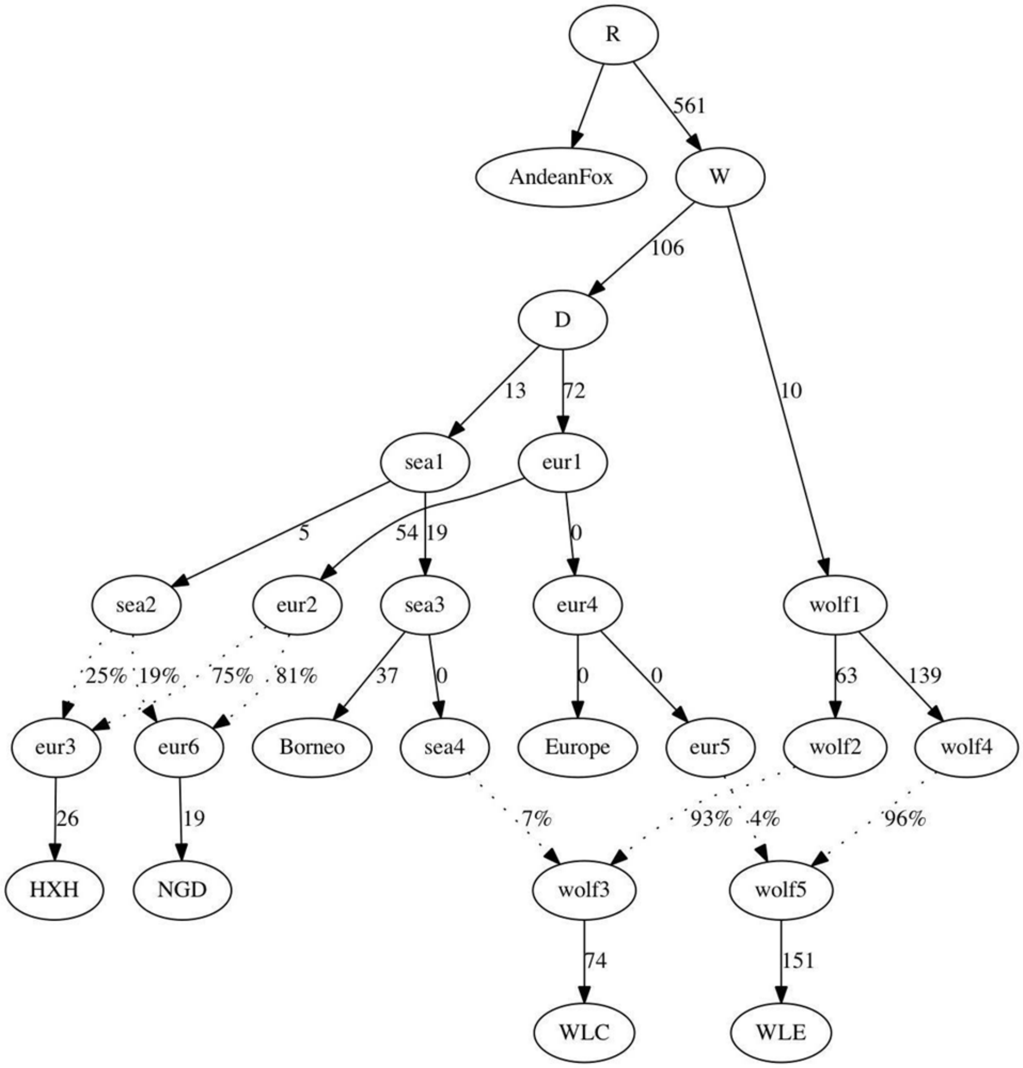

Supplementary Figure 48: Best fit admixture graph for HXH and NGD when including modern European and Southeast Asian village dogs and wolves.

Supplementary Figure 49

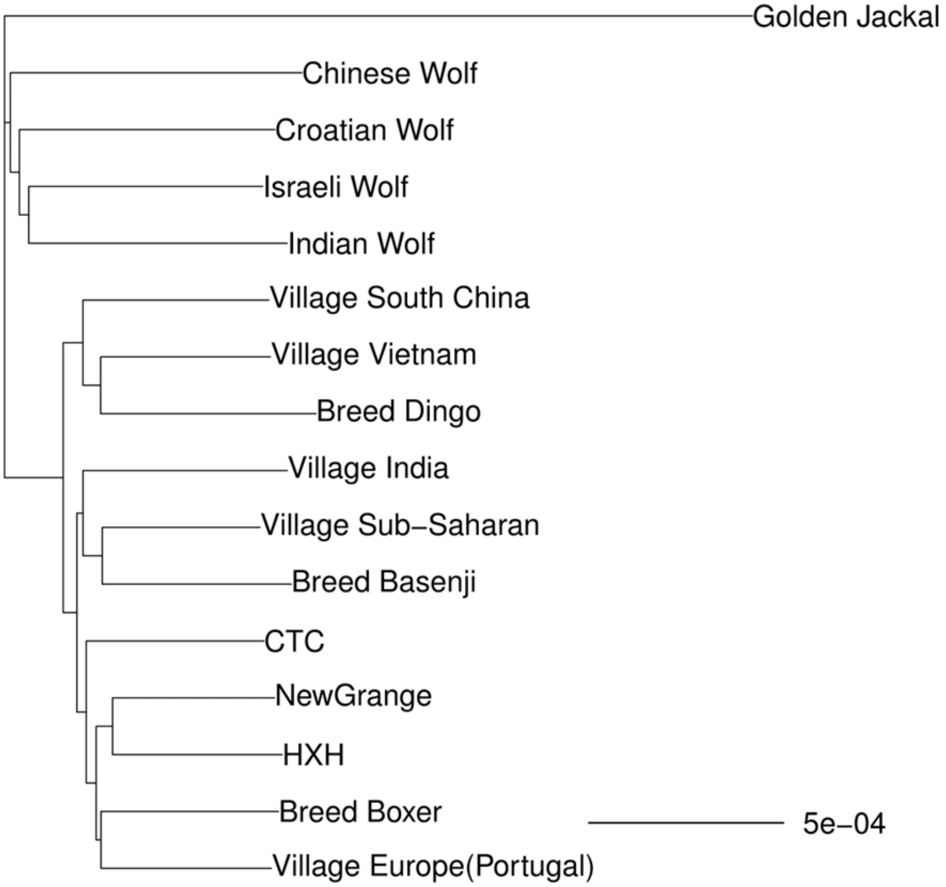

Supplementary Figure 49: NJ tree based on 16,434 "neutral" loci of selected samples used in G-PhoCS.

Supplementary Figure 50

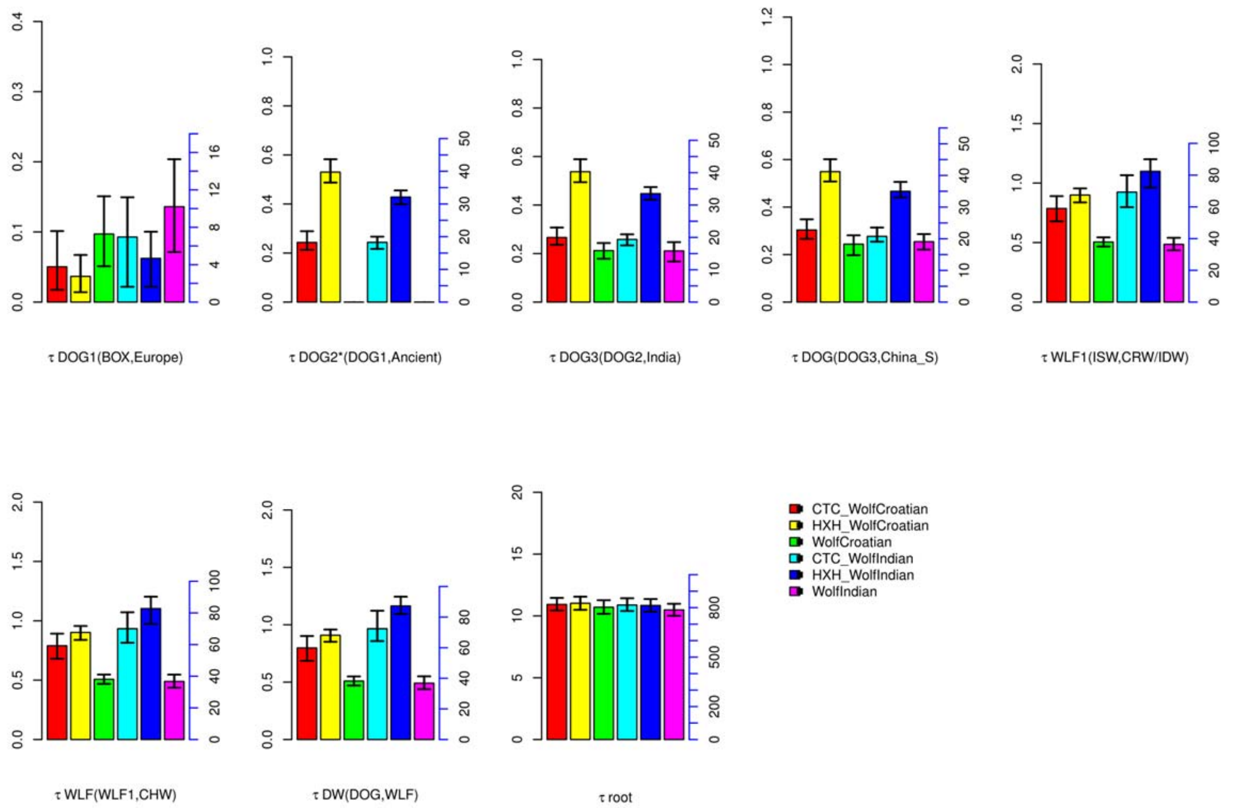

Supplementary Figure 50: Divergence time estimates in G-PhoCS analysis when including and excluding ancient samples. We use the tree of the form (((((boxer, Village_Europe), Village_India), Village_ChinaS), ((wolfY, IsraeliWolf), ChineseWolf)), GoldenJackal), with or without one ancient sample sister to the ancestral of boxer and Village_Europe: (1) wolfY as CroatianWolf, with CTC; (2) wolfY as CroatianWolf, with HXH; (3) wolfY as CroatianWolf, no ancient sample; (4) wolfY as IndianWolf, with CTC; (5) wolfY as IndianWolf, with HXH; (6) wolfY as IndianWolf, no ancient sample. Raw estimates on the left axis (scaled up by 1e04) and calibrated estimates on the right axis (in 1,000 years). This analysis used 5000 randomly selected loci.

Supplementary Figure 51

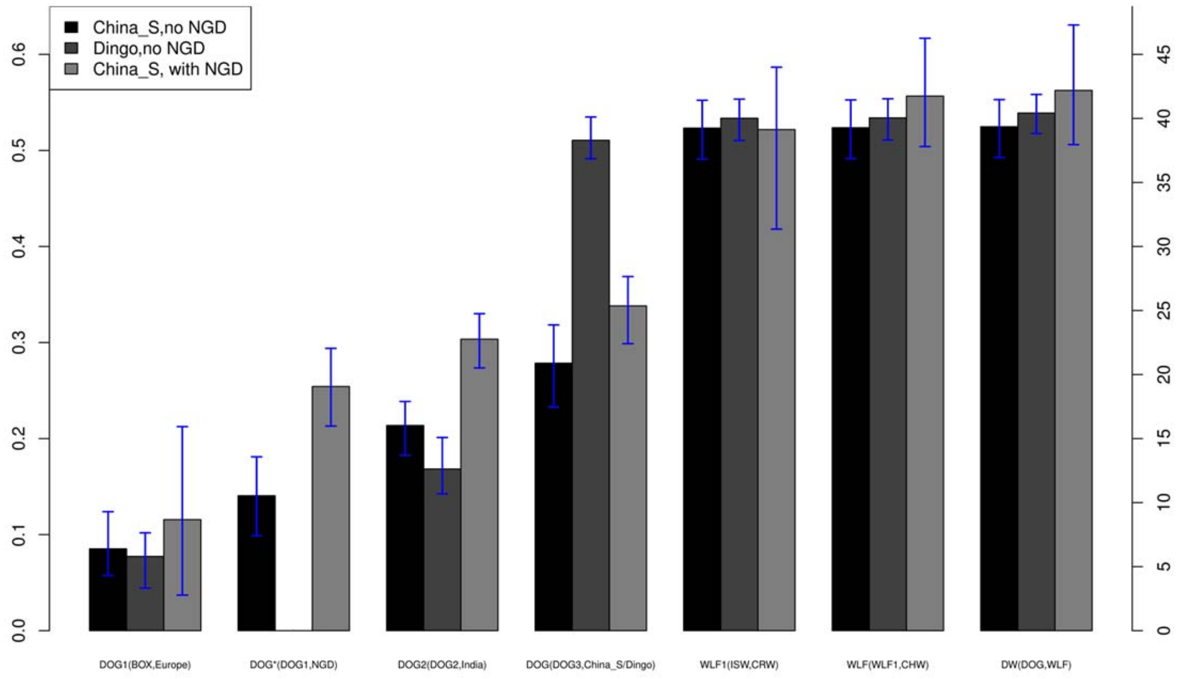

Supplementary Figure 51: Comparison of divergence time estimates with/without NGD in G-PhoCS analysis. We use the tree of the form (((((boxer, Village_Europe), Village_India), Village_ChinaS or dingo), ((CroatianWolf, IsraeliWolf), ChineseWolf)), GoldenJackal), with or without one ancient sample sister to the ancestral of boxer and Village_Europe. The divergence time for (DOG1,NGD) when NGD is not in G-PhoCS was estimated using a numerical approach (See Supplementary Note 13). Axis on the left are raw estimates, scaled up by 1e04. Axis on the right are recalibrated estimates in thousand years, assuming mutation rate $4x10^{-9}$ per/generation, generation time 3 years.

Supplementary Figure 52

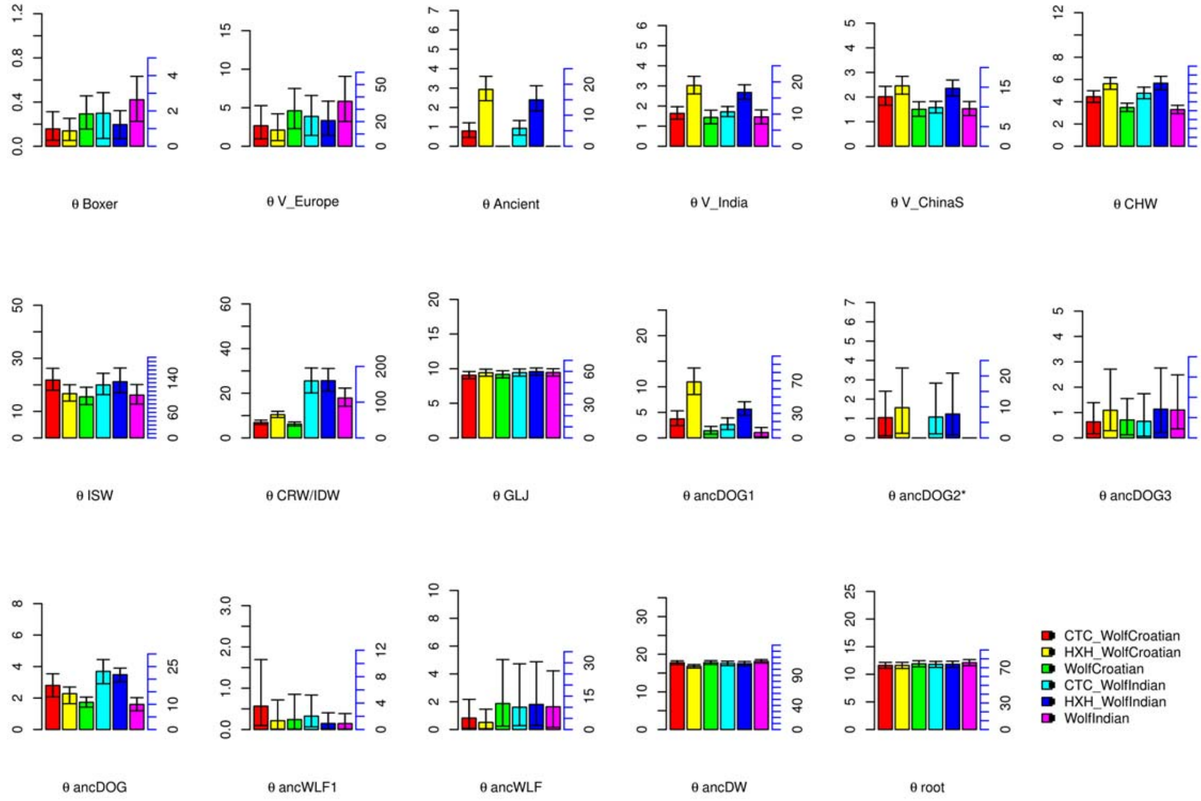

Supplementary Figure 52: Population size estimates from G-PhoCS analysis. We use the tree in form (((((Boxer, Village_Europe), Village_India), Village_ChinaS), ((wolfY, IsraeliWolf), ChineseWolf)), GoldenJackal), with or without one ancient sample sister to the ancestral of boxer and Village_Europe: (1) wolfY as CroatianWolf, with CTC; (2) wolfY as CroatianWolf, with HXH; (3) wolfY as CroatianWolf, no ancient sample; (4) wolfY as IndianWolf, with CTC; (5) wolfY as IndianWolf, with HXH; (6) wolfY as IndianWolf, no ancient sample. Raw estimates on the left axis (scaled up by $1x10^4$) and calibrated estimates on the right axis (in thousands of individuals). This analysis used 5,000 randomly selected loci.

Supplementary Figure 53

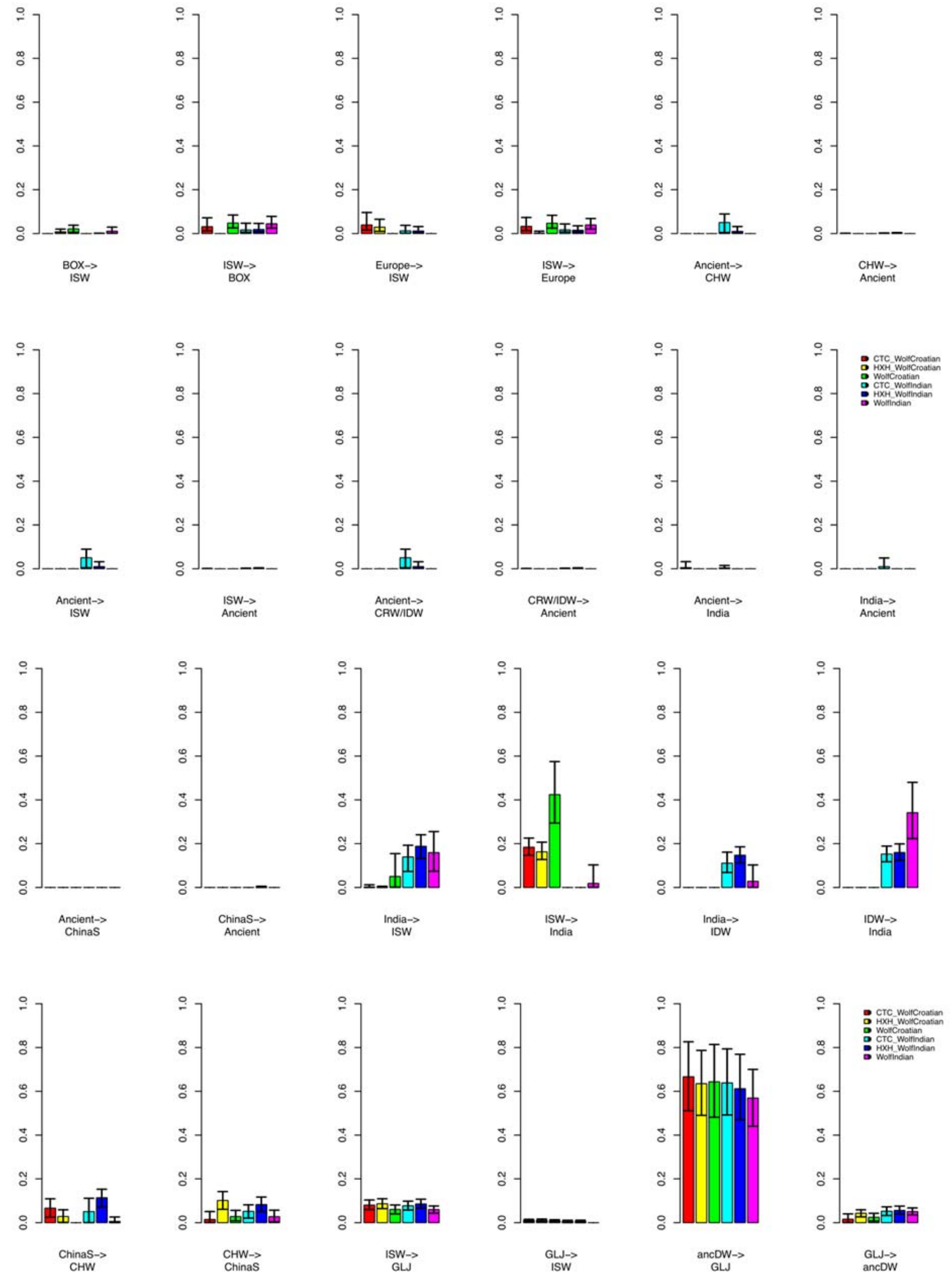

Supplementary Figure 53: Total migration rate estimates in G-PhoCS analysis. We use the tree of the form (((((Boxer, Village_Europe), Village_India), Village_ChinaS), ((wolfY, IsraeliWolf), ChineseWolf)), GoldenJackal), with or without one ancient sample sister to the ancestral of boxer and Village_Europe: (1) wolfY as CroatianWolf, with CTC; (2) wolfY as CroatianWolf, with HXH; (3) wolfY as CroatianWolf, no ancient sample; (4) wolfY as IndianWolf, with CTC; (5) wolfY as IndianWolf, with HXH; (6) wolfY as IndianWolf, no ancient sample. This analysis used 5,000 randomly selected loci.

Supplementary Figure 54

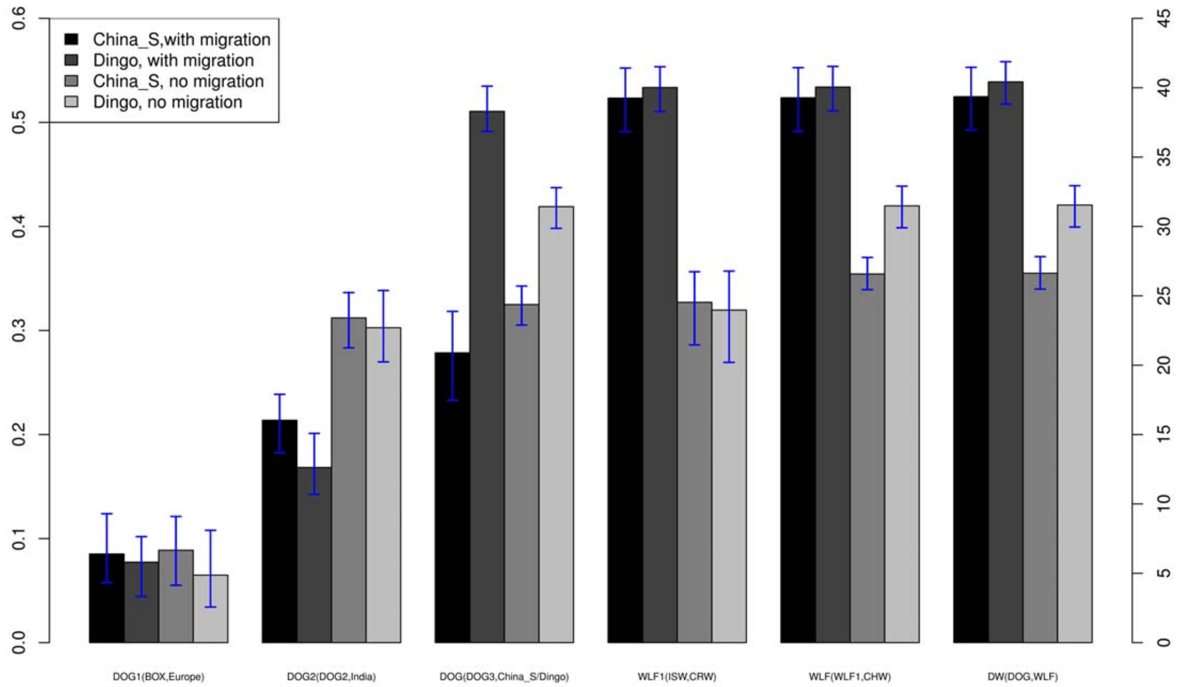

Supplementary Figure 54: Comparison of divergence time estimates with/without migration setting in G-PhoCS analysis. We use the tree in form (((((Boxer, Village_Europe), Village_India), Village_ChinaS or dingo), ((CroatianWolf, IsraeliWolf), ChineseWolf)), GoldenJackal), with or without migration setting. Axis on the left are raw estimates, scaled up by 1e04. Axis on the right are recalibrated estimates in thousand years, assuming mutation rate $4x10^{-9}$ per/generation, generation time 3 years. This analysis used 16,434 loci.

Supplementary Figure 55

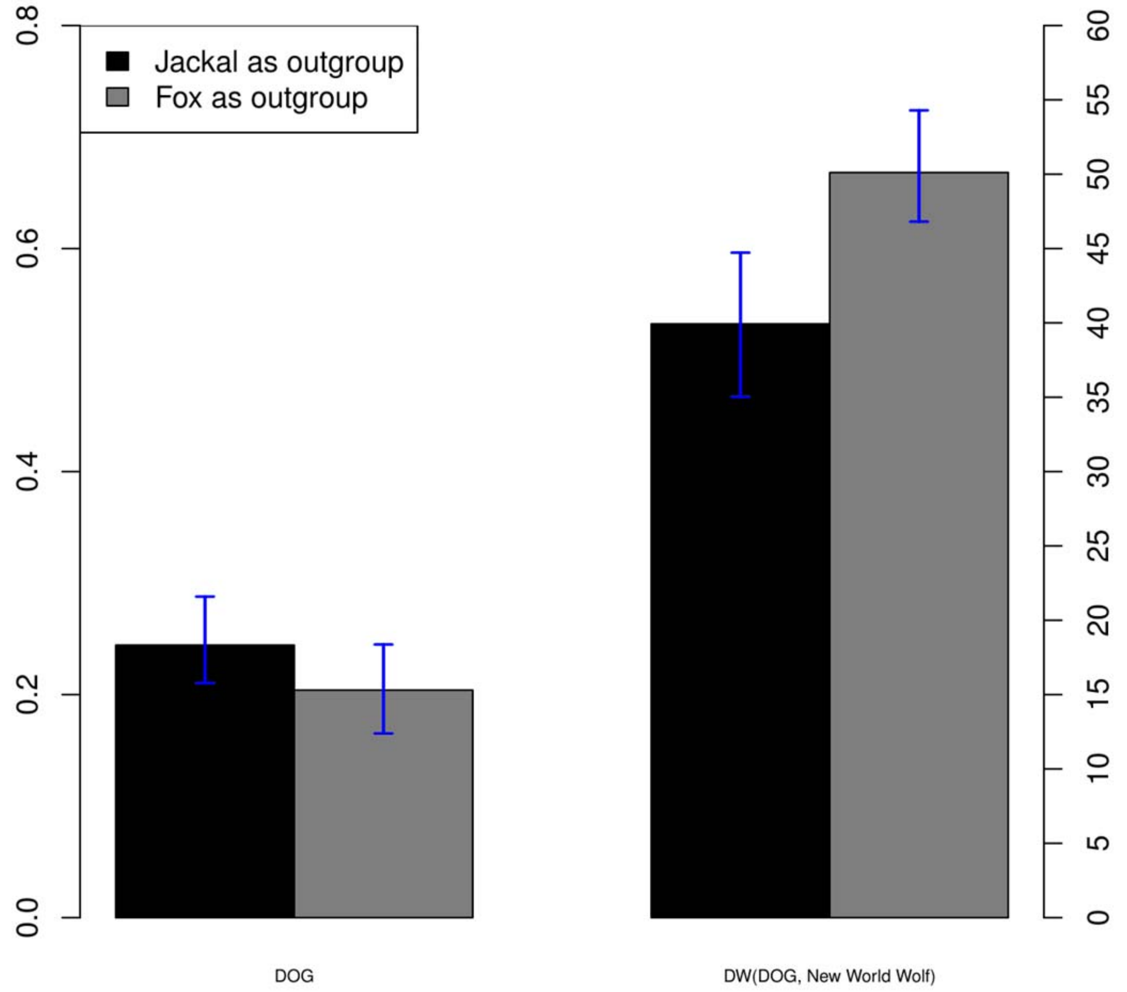

Supplementary Figure 55: Comparison of divergence time estimates using different outgroups. We use the tree of the form ((((Village_Europe1,Village_Europe2), (Village_ChinaS1, Village_ChinaS2)), (yellowstoneWolf1, yellowstoneWolf2)), outgroup), with outgroup being either golden jackal or Andean fox. Axis on the left are raw estimates, scaled up by $1x10^4$. Axis on the right are recalibrated estimates in thousand years, assuming mutation rate $4x10^{-9}$ per/generation, generation time 3 years. This analysis used 5,000 randomly selected loci.

Supplementary Figure 56

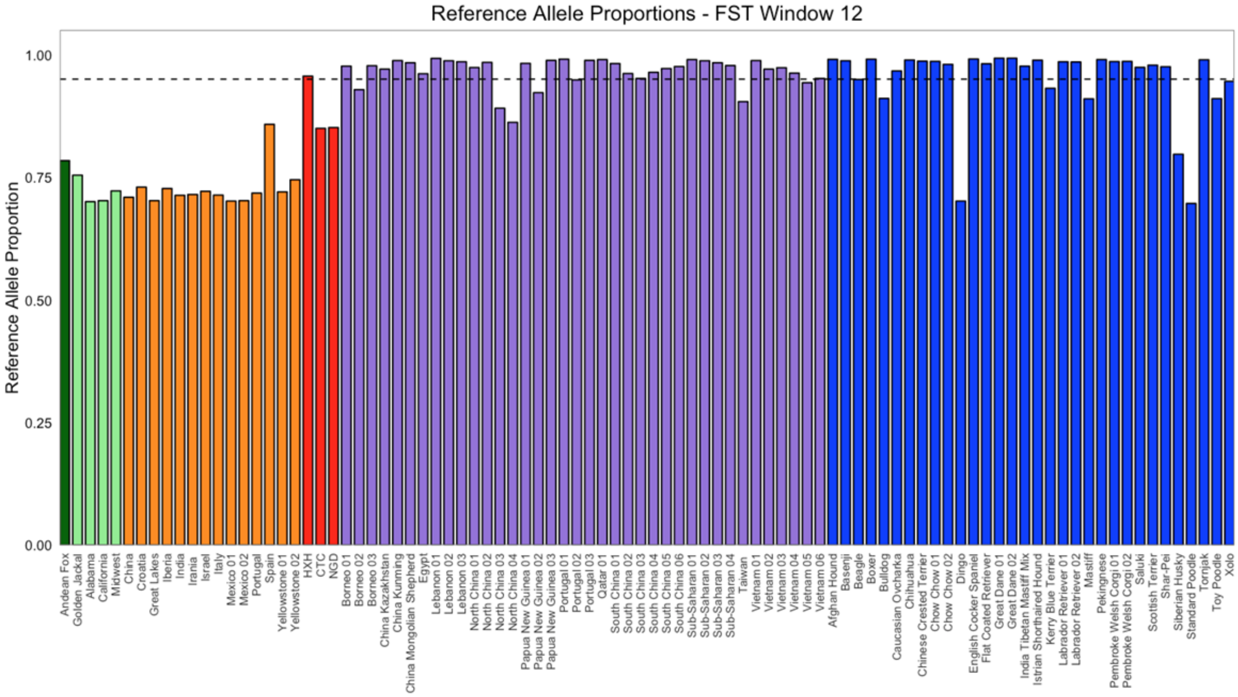

Supplementary Figure 56: Average reference allele count per sample in the *AMY2B* locus (F_{ST} Window 12). The colors are as follows: Andean fox (green), golden jackal and coyotes (light green), wolves (orange), ancient dogs (red), village dogs (purple), and breed dogs (blue). The dashed line indicates the 0.95 average reference allele threshold used to determine whether a sample was dog-like or wild-like.

Supplementary Figure 57

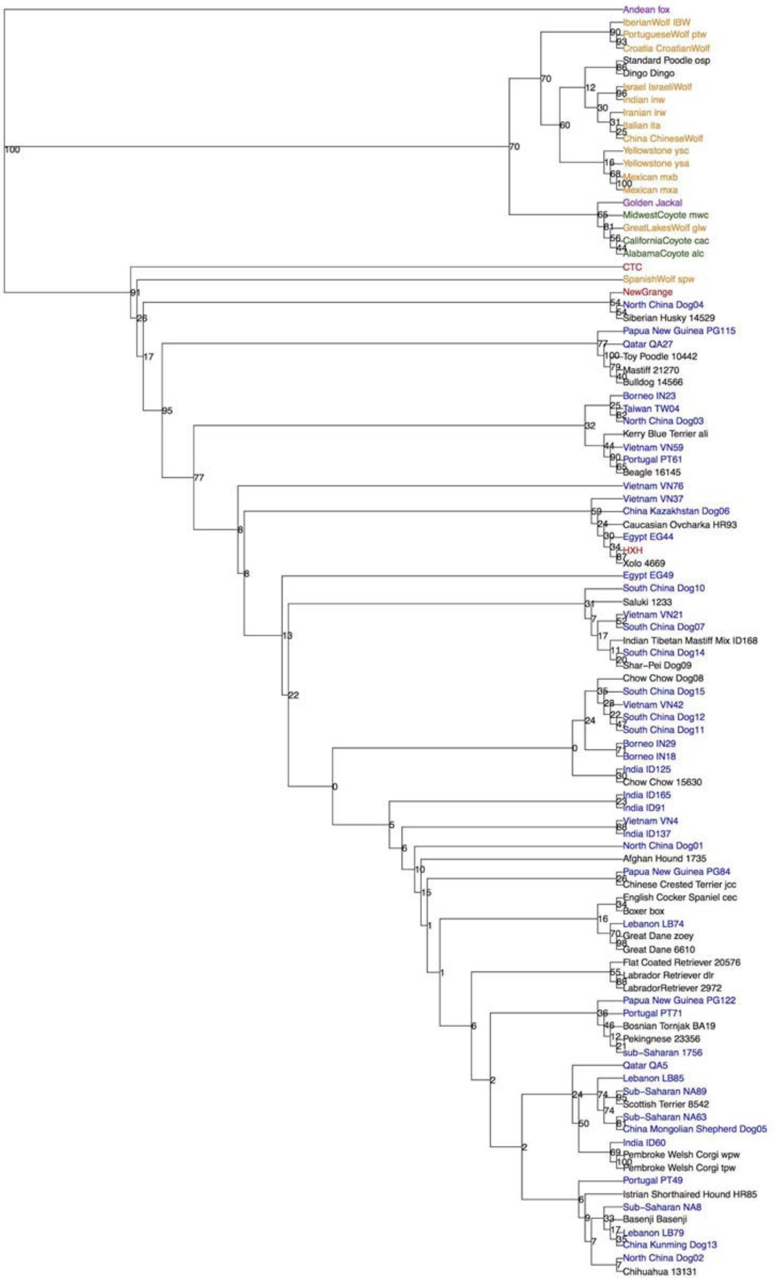

Supplementary Figure 57: NJ tree (100 bootstraps) for 1231 SNPs within the F_{ST} Window 12. Bootstrap values are indicated at each node. Sample types are distinguished by color: ancient dogs (red), breed dogs (black), village dogs (blue), wolves (yellow), coyote (green), and golden jackal (purple). Also indicated at each branch are the sample identifiers and geographic origin (if available). Branch lengths are proportional to ease viewing.

Supplementary Figure 58

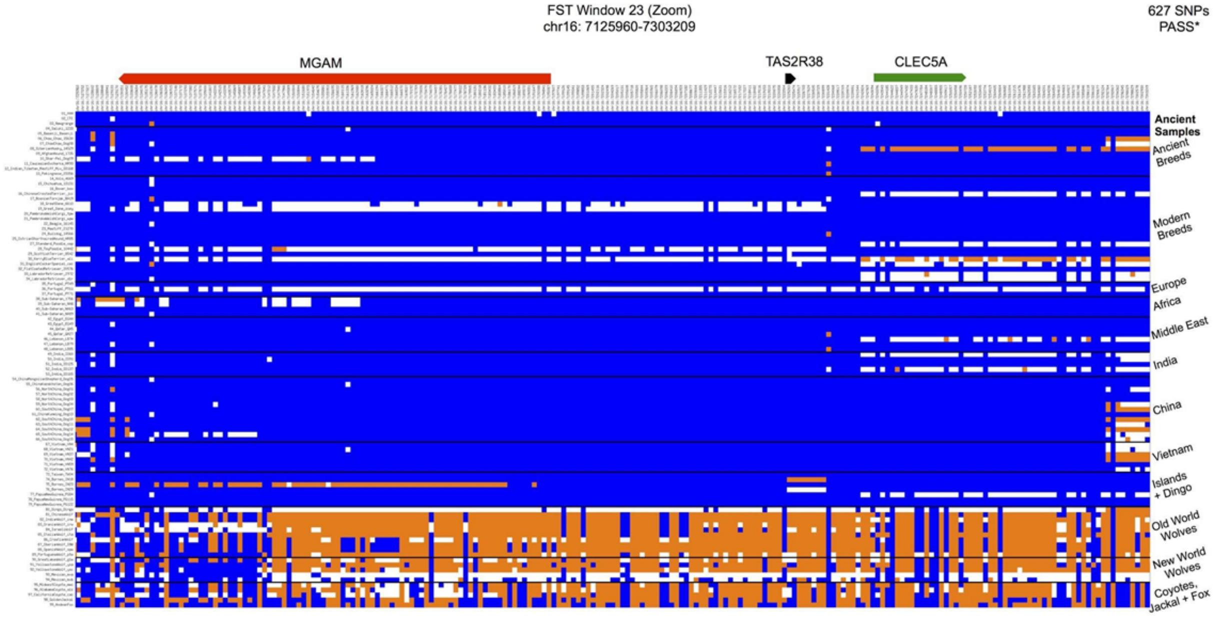

Supplementary Figure 58: A zoomed-in view of the less-variable portion of Axelsson *et al.* F_{ST}-derived domestication locus 23 (chr16: 7125960-7303209). The identifiers of each dog (left) and their broader classification or geographic origin (right) are provided. Genotypes for each SNP (along the top) are colored as sites homozygous for the reference allele (0/0) are blue, heterozygous sites (0/1) are white, and homozygous (1/1) for the alternate allele are orange. Gene models and orientation are above for *MGAM* (red), *TAS2R38* (black), and *CLEC5A* (green).

Supplementary Figure 59

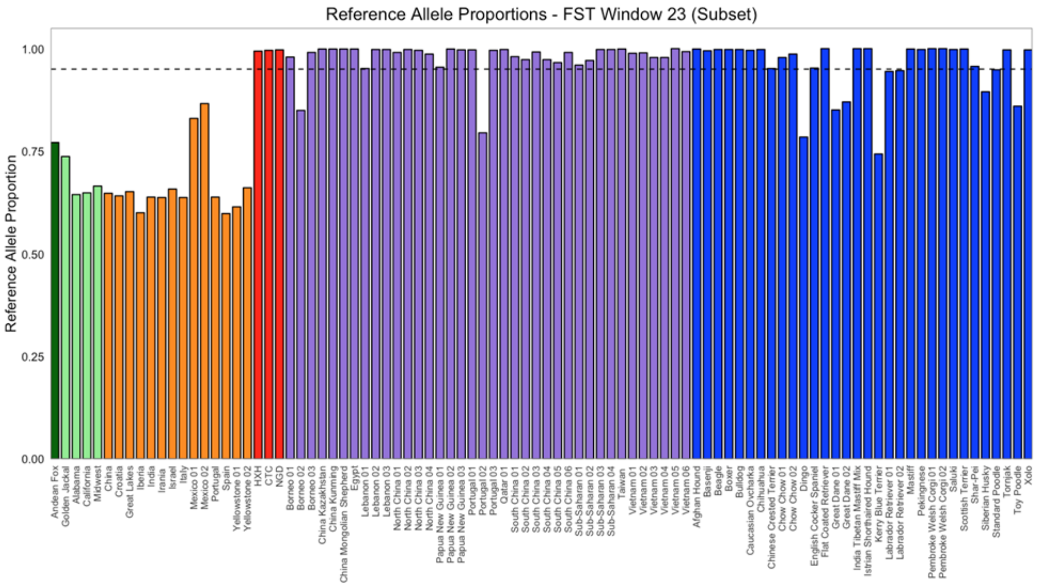

Supplementary Figure 59 - Average reference allele counts for SNP sites in the less variable subset of the *MGAM* locus (F_{ST} Window 23; chr16: 7125960-7303209). The colors are as follows: Andean fox (green), golden jackal and coyotes (light green), wolves (orange), ancient dogs (red), village dogs (purple), and breed dogs (blue). The dashed line indicates the 0.95 reference allele proportion threshold used to determine whether a sample was dog-like or wild-like.

Supplementary Figure 60

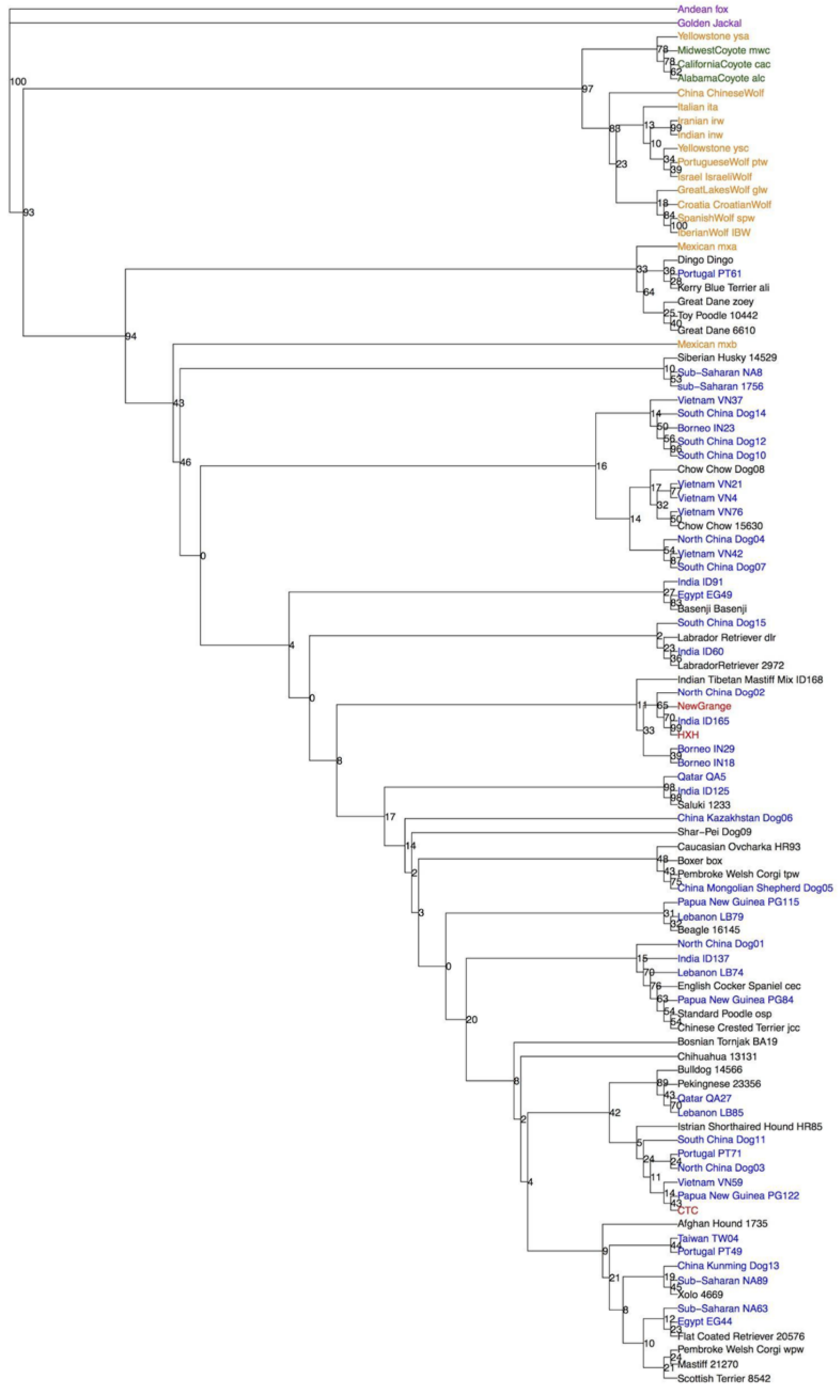

Supplementary Figure 59. NJ tree (100 bootstraps) for 1,448 SNPs within the F_{ST} Window 23. Bootstrap values are indicated at each node. Sample types are distinguished by color: ancient dogs (red), breed dogs (black), village dogs (blue), wolves (yellow), coyote (green), and golden jackal (purple). Also indicated at each branch are the sample identifiers and geographic origin (if available). Branch lengths are proportional to ease viewing

Supplementary Table 1: Neolithic sub-divisions in Southern Germany.

**Sub-division**	**Culture**	**Period**
Early Neolithic	Linear Pottery Culture	5,400-4,900 BCE cal.
Middle Neolithic	Hinkelstein/Stroked Pottery/Großgartach/Rössen	4,900-4,300 BCE cal.
Younger Neolithic	Michelsberg Culture/Altheim	5,300-3,500 BCE cal.
Late Neolithic	Horgen/Goldberg III/Cham/Wartberg/Bernburg	3,500-2,600 BCE cal.
End Neolithic	Corded Ware Culture/Bell Beaker Culture	2,800-2,000 BCE cal.

Supplementary Table 2: HiSeq sequencing statistics for ancient samples.

Sample	Total Reads sequenced	Merged reads mapped after filtering	Duplicate Reads	% Endogenous DNA (flagstat)	Coverage	Mean fragment length	% 5’ C>T	% 3’ G>A
HXH	693,443,481	433,815,523	56,068,409	67.21%	9x	60bp	34.8	35.8
CTC	563,180,319	414,120,523	108,815,544	77.29%	9x	70bp	28.2	28.3
NGD	1,310,607,189	NA	198,486,473	99.64%	25x	58bp	26.3	25.6

Note, unlike during MiSeq screening, endogenous DNA here is estimated based on the proportion of reads submitted to BWA that mapped without any quality score filtering. Endogenous DNA content is almost 100% for NGD as we only obtained mapped reads.

Supplementary Table 3. Sample information for SNP array datasets.

Sample Description	Source	Canine type	location	short-code	# of individuals
AncientDog	this study	AncientDog	AncientDog	CTC	1
AncientDog	this study	AncientDog	AncientDog	HXH	1
AncientDog	this study	AncientDog	AncientDog	NGD	1
-	Shannon	Breed	-	br	4848
Chinese Wolf	Freedman	Wolf	ChineseWolf	WLC	1
Croatian Wolf	Freedman	Wolf	CroatianWolf	WLE	1
Golden Jackal	Freedman	Jackal	Jackal	Jackal	1
Israeli Wolf	Freedman	Wolf	IsraeliWolf	WIS	1
Village Dog Afghanistan	Shannon	VillageDog	Afghanistan	Afg	13
Village Dog Belize	Shannon	VillageDog	Americas	Bel	4
Village Dog Bosnia	Shannon	VillageDog	Europe	Bos	3
Village Dog Brazil	Shannon	VillageDog	Americas	Bra	12
Village Dog Brazil	Shannon	VillageDog	Europe	Bra	1
Village Dog Burkina Faso	Shannon	VillageDog	Africa	Bur	2
Village Dog Colombia	Shannon	VillageDog	Americas	Col	9
Village Dog Costa Rica	Shannon	VillageDog	Americas	Cos	8
Village Dog Croatia	Shannon	VillageDog	Europe	Cro	6
Village Dog DRC-Boende	Shannon	VillageDog	Africa	DRC	15
Village Dog DRC-Katanga	Shannon	VillageDog	Africa	DRC	12
Village Dog DRC-Kinshasa	Shannon	VillageDog	Africa	DRC	6
Village Dog Dominican Republic	Shannon	VillageDog	Americas	Dom	12
Village Dog Egypt-Giza	Shannon	VillageDog	Egypt	Egy	3
Village Dog Egypt-Kharga	Shannon	VillageDog	Egypt	Egy	1
Village Dog Egypt-Luxor	Shannon	VillageDog	Egypt	Egy	12
Village Dog Fiji-Kadavu	Shannon	VillageDog	Islands	Fij	12
Village Dog Fiji-Tavenui	Shannon	VillageDog	Islands	Fij	1
Village Dog Fiji-Viti Levu	Shannon	VillageDog	Islands	Fij	16
Village Dog French Polynesia-Marquesas-	Shannon	VillageDog	Islands	Fre	12

Hiva Oa					
Village Dog French Polynesia-Society Islands-Bora Bora	Shannon	VillageDog	Islands	Fre	5
Village Dog French Polynesia-Society Islands-Huahine	Shannon	VillageDog	Islands	Fre	8
Village Dog French Polynesia-Society Islands-Moorea	Shannon	VillageDog	Islands	Fre	5
Village Dog French Polynesia-Society Islands-Raiatea	Shannon	VillageDog	Islands	Fre	6
Village Dog Ghana	Shannon	VillageDog	Africa	Gha	5
Village Dog Guinea	Shannon	VillageDog	Africa	Gui	1
Village Dog Honduras	Shannon	VillageDog	Americas	Hon	8
Village Dog India-Chennai	Shannon	VillageDog	India	I-C	6
Village Dog India-Dehli	Shannon	VillageDog	India	I-D	6
Village Dog India-Hazaribagh	Shannon	VillageDog	India	I-H	2
Village Dog India-Mumbai	Shannon	VillageDog	India	I-M	6
Village Dog India-Orissa	Shannon	VillageDog	India	I-O	6
Village Dog India-West Bengal	Shannon	VillageDog	India	I-W	6
Village Dog Indonesia-Borneo	Shannon	VillageDog	Indonesia	InB	9
Village Dog Indonesia-Jakarta	Shannon	VillageDog	Indonesia	InJ	2
Village Dog Iraq	Shannon	VillageDog	MiddleEast	Ira	1
Village Dog Lebanon-Bekaa	Shannon	VillageDog	MiddleEast	Leb	7
Village Dog Lebanon-Beruit	Shannon	VillageDog	Africa	Leb	1
Village Dog Lebanon-Beruit	Shannon	VillageDog	MiddleEast	Leb	8
Village Dog Liberia-Lofa	Shannon	VillageDog	Africa	Lib	18
Village Dog Liberia-Monrovia	Shannon	VillageDog	Africa	Lib	4
Village Dog Mexico-Mexico City	Shannon	VillageDog	Americas	Mex	6
Village Dog Mexico-Morelia	Shannon	VillageDog	Americas	Mex	9
Village Dog Mongolia	Shannon	VillageDog	CentralAsia	Mon	13
Village Dog Namibia-Central	Shannon	VillageDog	Americas	Nam	1
Village Dog Namibia-Northern	Shannon	VillageDog	Africa	Nam	14
Village Dog Nepal	Shannon	VillageDog	CentralAsia	Nep	12

Village Dog Nigeria	Shannon	VillageDog	Africa	Nig	1
Village Dog Palau	Shannon	VillageDog	Americas	Pal	1
Village Dog Panama	Shannon	VillageDog	Americas	Pan	2
Village Dog Papua New Guinea-East Highlands	Shannon	VillageDog	PNG	Pap	11
Village Dog Papua New Guinea-Port Moresby	Shannon	VillageDog	PNG	Pap	9
Village Dog Peru-Arequipa	Shannon	VillageDog	Americas	Per	16
Village Dog Peru-Cusco	Shannon	VillageDog	Americas	Per	25
Village Dog Peru-Ica	Shannon	VillageDog	Americas	Per	8
Village Dog Peru-Loreto	Shannon	VillageDog	Americas	Per	25
Village Dog Peru-Puno	Shannon	VillageDog	Americas	Per	17
Village Dog Portugal	Shannon	VillageDog	Europe	Por	9
Village Dog Puerto Rico	Shannon	VillageDog	Americas	Pue	9
Village Dog Qatar	Shannon	VillageDog	MiddleEast	Qat	9
Village Dog Solomon Islands-Central	Shannon	VillageDog	Islands	Sol	4
Village Dog Solomon Islands-Guadalcanal	Shannon	VillageDog	Islands	Sol	3
Village Dog Solomon Islands-Makira	Shannon	VillageDog	Islands	Sol	4
Village Dog Solomon Islands-Western	Shannon	VillageDog	Islands	Sol	4
Village Dog Sudan	Shannon	VillageDog	Africa	Sud	1
Village Dog Turkey-Giresun	Shannon	VillageDog	MiddleEast	Tur	3
Village Dog Turkey-Istanbul	Shannon	VillageDog	MiddleEast	Tur	10
Village Dog US-Alaska	Shannon	VillageDog	Arctic	US	11
Village Dog Uganda	Shannon	VillageDog	Africa	Uga	12
Village Dog Vietnam-Cao Bang	Shannon	VillageDog	Vietnam	Vie	6
Village Dog Vietnam-Ha Giang	Shannon	VillageDog	Vietnam	Vie	5
Village Dog Vietnam-Lang Son	Shannon	VillageDog	Vietnam	Vie	5
Village Dog Vietnam-Lao Cai	Shannon	VillageDog	Vietnam	Vie	4
free-breeding dog	Pilot	VillageDog	Armenia	pAR	25
free-breeding dog	Pilot	VillageDog	Bulgaria	pBU	9
free-breeding dog	Pilot	VillageDog	Central	pCR	16

			Russia		
free-breeding dog	Pilot	VillageDog	China	pCH	9
free-breeding dog	Pilot	VillageDog	East Russia	pER	19
free-breeding dog	Pilot	VillageDog	Iraq	pIR	8
free-breeding dog	Pilot	VillageDog	Kazakhstan	pKA	20
free-breeding dog	Pilot	VillageDog	Mongolia	pMO	27
free-breeding dog	Pilot	VillageDog	Poland	pPO	21
free-breeding dog	Pilot	VillageDog	Saudi Arabia 1	pSA	22
free-breeding dog	Pilot	VillageDog	Saudi Arabia 2	pSA	5
free-breeding dog	Pilot	VillageDog	Slovenia	pSL	13
free-breeding dog	Pilot	VillageDog	Tajikistan	pTA	19
free-breeding dog	Pilot	VillageDog	Thailand	pTH	21
wolfIberian	from genome dataset	Wolf	wolfIberian	WLE	1
wolfIndia	from genome dataset	Wolf	wolfIndia	WIN	1
wolfIran	from genome dataset	Wolf	wolfIran	WIR	1
wolfItaly	from genome dataset	Wolf	wolfItaly	WLE	1
wolfMexico	from genome dataset	Wolf	wolfMexico	WLA	2
wolfPortugal	from genome dataset	Wolf	wolfPortugal	WLE	1
wolfSpain	from genome dataset	Wolf	wolfSpain	WLE	1
wolfYellowstone	from genome dataset	Wolf	wolfYellowstone	WLA	3
wolfgreatLakes	from genome dataset	Wolf	wolfgreatLakes	WLA	1

Supplementary Table 4: Sample descriptions for whole-genome sequenced canines used for variant calling. Our unique sample idenitifier, sex, canine description, origin of sample (if available), SRA project (if available) and sample identifiers, and published study are provided for each sample used in our whole-genome analysis. Asterisks (*) indicated subset of read data from samples were described in Decker *et al.*. Raw sequencing files were obtained for all other samples from which variants were called.

Sample ID	Sex	Auto Coverage	Canine Type	Sample Description	Geographic Origin	SRA Project ID	SRA Sample ID	Publication
CTC	M	9	Ancient	CTC	Germany	PRJNA319283	SRS14077451	This study
HXH	M	9	Ancient	HXH	Germany	PRJNA319283	SRS1407453	This study
NGD	M	28	Ancient	Newgrange	Ireland	PRJEB13070	-	Frantz *et al.*
1735	F	8	Breed	Afghan Hound	n/a	PRJNA232497	SRS520039	Auton *et al.*
Basenji	M	19	Breed	Basenji	U.S.	PRJNA274504	SRS1025426	Freedman *et al.*
16145	M	7	Breed	Beagle	n/a	SRP073312	SRS1397614	Freedman *et al.* Schoenebeck *et al.*
box	F	23	Breed	Boxer	n/a	PRJNA255370	SRS661481	Fan *et al.*
14566	F	14	Breed	Bulldog	n/a	PRJNA288568	SRS984782	Freedman *et al.* Schoenebeck *et al.*
HR93	F	11	Breed	Caucasian Ovcharka	Bosnia	PRJNA232497	SRS520060	Auton *et al.*
13131	F	6	Breed	Chihuahua	n/a	PRJNA288568	SRS984783	Freedman *et al.* Schoenebeck *et al.*
jcc	M	22	Breed	Chinese Crested Terrier	U.K. (England)	PRJNA255370	SRS661484	Fan *et al.*
15630	F	13	Breed	Chow Chow	n/a	SRP073312	SRS1397613	Freedman *et al.* Schoenebeck *et al.*
cec	M	10	Breed	English Cocker Spaniel	n/a	PRJNA255370	SRS661482	Fan *et al.*
20576	F	8	Breed	Flat Coated Retreiver	n/a	SRP073312	SRS1397625	Freedman *et al.* Schoenebeck *et al.*
6610	F	7	Breed	Great Dane	n/a	PRJNA288568	SRS984791	Freedman *et al.* Schoenebeck *et al.*
zoe	F	19	Breed	Great Dane	U.S. (Michigan)	SRP073312	SRS1397510	This study
HR85	F	7	Breed	Istrian Shorthaired Hound	Bosnia	PRJNA232497	SRS520059	Auton *et al.*
ali	M	18	Breed	Kerry Blue Terrier	n/a	PRJNA255370	SRS661480	Fan *et al.*
dlr	M	12	Breed	Labrador Retriever	n/a	PRJNA255370	SRS661483	Fan *et al.*
2972	M	10	Breed	Labrador Retriever	n/a	PRJNA232497	SRS520040	Auton *et al.*
21270	F	13	Breed	Mastiff	n/a	PRJNA288568	SRS984789	Freedman *et al.* Schoenebeck *et al.*
23356	F	9	Breed	Pekingnese	n/a	PRJNA288568	SRS984793	Freedman *et al.* Schoenebeck *et al.*
tpw	F	9	Breed	Pembroke Welsh Corgi	n/a	PRJNA263947	SRS732550	
wpw	M	7	Breed	Pembroke Welsh Corgi	n/a	PRJNA263947	SRS732549	

1233	F	9	Breed	Saluki	n/a	PRJNA288568	SRS984796	Freedman *et al.* Schoenebeck *et al.*
8542	F	8	Breed	Scottish Terrier	n/a	PRJNA288568	SRS984798	Freedman *et al.* Schoenebeck *et al.*
14529	F	8	Breed	Siberian Husky	n/a	PRJNA288568	SRS984799	Freedman *et al.* Schoenebeck *et al.*
osp	M	14	Breed	Standard Poodle	U.K. (England)	PRJNA255370	SRS661485	Fan *et al.*
BA19	M	6	Breed	Tornjak	Bosnia	PRJNA232497	SRS520042	Auton *et al.*
10442	F	8	Breed	Toy Poodle	n/a	PRJNA288568	SRS984800	Freedman *et al.* Schoenebeck *et al.*
4669	M	17	Breed	Xoloitzcuintli	n/a	PRJNA232497	SRS520041	Auton *et al.*
ID168	M	10	Breed	India Tibetan Mastiff Mix	India	PRJNA232497	SRS520064	Auton *et al.*
alc	M	6	Coyote	Coyote_Alabama	U.S. (Alabama)	PRJNA255370	SRS661478	Fan *et al.*
mwc	M	8	Coyote	Coyote_Midwest	U.S. (Midwest)	PRJNA255370	SRS661479	Fan *et al.*
cac	M	33	Coyote	Coyote_California	U.S. (California)	PRJNA255370	SRS661477	Fan *et al.*
Dingo	M	19	Breed	Dingo	Australia	PRJNA274504	SRS1025425	Freedman *et al.*
Golden Jackal	F	24	Jackal	Golden Jackal	Israel	PRJNA274504	SRS1025419	Freedman *et al.*
PT49	M	16	Village Dog	Portugal	Portugal	SRP034749	SRS1397563	This study
PT61	F	18	Village Dog	Portugal	Portugal	PRJNA232497	SRS520079	Auton *et al.*
PT71	M	16	Village Dog	Portugal	Portugal	PRJNA232497	SRS520080	Auton *et al.*
ID125	M	15	Village Dog	India	India	PRJNA232497	SRS520061	Auton *et al.*
ID137	M	8	Village Dog	India	India	PRJNA232497	SRS520062	Auton *et al.*
ID165	M	10	Village Dog	India	India	PRJNA232497	SRS520063	Auton *et al.*
ID60	M	15	Village Dog	India	India	PRJNA232497	SRS520065	Auton *et al.*
ID91	F	8	Village Dog	India	India	PRJNA232497	SRS520066	Auton *et al.*
EG44	M	9	Village Dog	Egypt	Egypt	PRJNA232497	SRS520057	Auton *et al.*
EG49	M	8	Village Dog	Egypt	Egypt	PRJNA232497	SRS520058	Auton *et al.*
LB74	M	7	Village Dog	Lebanon	Lebanon	PRJNA232497	SRS520070	Auton *et al.*
LB79	M	9	Village Dog	Lebanon	Lebanon	PRJNA232497	SRS520071	Auton *et al.*
LB85	F	10	Village Dog	Lebanon	Lebanon	PRJNA232497	SRS520072	Auton *et al.*
QA27	F	12	Village Dog	Qatar	Qatar	PRJNA232497	SRS520081	Auton *et al.*
QA5	M	7	Village Dog	Qatar	Qatar	PRJNA232497	SRS520082	Auton *et al.*

Dog07	F	7	Village Dog	South China	South China, Xilin	PRJNA232497	SRS520048	Auton *et al.*
Dog08	M	7	Breed	Chow Chow	South China	PRJNA232497	SRS520049	Auton *et al.*
Dog09	M	7	Breed	Shar-Pei	South China	PRJNA232497	SRS520050	Auton *et al.*
Dog10	F	8	Village Dog	South China	South China, Sichuan Liangshan	PRJNA232497	SRS520051	Auton *et al.*
Dog11	M	7	Village Dog	South China	South China, Sichuan Qinchuan	PRJNA232497	SRS520052	Auton *et al.*
Dog12	M	7	Village Dog	South China	South China, Chongqing	PRJNA232497	SRS520053	Auton *et al.*
Dog14	M	8	Village Dog	South China	South China, Guizhou Xiasi	PRJNA232497	SRS520055	Auton *et al.*
Dog15	F	7	Village Dog	South China	South China	PRJNA232497	SRS520056	Auton *et al.*
IN18	M	6	Village Dog	Borneo	Borneo	PRJNA232497	SRS520067	Auton *et al.*
IN23	F	6	Village Dog	Borneo	Borneo	PRJNA232497	SRS520068	Auton *et al.*
IN29	M	6	Village Dog	Borneo	Borneo	PRJNA232497	SRS520069	Auton *et al.*
PG115	F	6	Village Dog	Papua New Guinea	Papua New Guinea	PRJNA232497	SRS520076	Auton *et al.*
PG122	F	7	Village Dog	Papua New Guinea	Papua New Guinea	PRJNA232497	SRS520077	Auton *et al.*
PG84	M	13	Village Dog	Papua New Guinea	Papua New Guinea	PRJNA232497	SRS520078	Auton *et al.*
TW04	F	17	Village Dog	Taiwan	Taiwan	PRJNA232497	SRS520083	Auton *et al.*
Dog01	F	7	Village Dog	North China	North China, Shandong	PRJNA232497	SRS520043	Auton *et al.*
Dog02	M	8	Village Dog	North China	North China, Shaanxi	PRJNA232497	SRS520044	Auton *et al.*
Dog03	M	8	Village Dog	North China	North China, Hebei	PRJNA232497	SRS520045	Auton *et al.*
Dog04	F	7	Village Dog	North China	North China, Mongolia	PRJNA232497	SRS520046	Auton *et al.*
Dog05	M	7	Village Dog	China Mongolian Shepherd	North China, Mongolia	SRP034749	SRS1397564	This study
Dog06	F	7	Village Dog	China Kazakhstan	North China, Kazakhstan	PRJNA232497	SRS520047	Auton *et al.*
Dog13	M	8	Village Dog	South China	South China, Kunming	PRJNA232497	SRS520054	Auton *et al.*
NA63	M	9	Village Dog	Sub-Saharan	Namibia	PRJNA232497	SRS520073	Auton *et al.*
NA8	F	15	Village Dog	Sub-Saharan	Namibia	PRJNA232497	SRS520074	Auton *et al.*
NA89	M	7	Village Dog	Sub-Saharan	Namibia	PRJNA232497	SRS520075	Auton *et al.*
1756	F	9	Village Dog	Sub-Saharan	Sub-Saharan Africa	SRP034749	SRS1397565	This study

VN21	F	10	Village Dog	Vietnam	Vietnam	PRJNA232497	SRS520084	Auton *et al.*
VN37	F	8	Village Dog	Vietnam	Vietnam	PRJNA232497	SRS520085	Auton *et al.*
VN4	M	9	Village Dog	Vietnam	Vietnam	PRJNA232497	SRS520086	Auton *et al.*
VN42	F	8	Village Dog	Vietnam	Vietnam	PRJNA232497	SRS520087	Auton *et al.*
VN59	M	10	Village Dog	Vietnam	Vietnam	PRJNA232497	SRS520088	Auton *et al.*
VN76	M	7	Village Dog	Vietnam	Vietnam	PRJNA232497	SRS520089	Auton *et al.*
Croatian Wolf	F	24	Wolf	wolf_Croatia	Croatia	PRJNA274504	SRS1025420	Freedman *et al.*
Israeli Wolf	F	24	Wolf	wolf_Israeli	Israel	PRJNA274504	SRS1025423	Freedman *et al.*
Chinese Wolf	F	24	Wolf	wolf_China	China	PRJNA274504	SRS1025418	Freedman *et al.*
inw	M	45	Wolf	wolf_India	India	PRJNA255370	SRS661487	Fan *et al.*
irw	F	28	Wolf	wolf_Iran	Iran	PRJNA255370	SRS661488	Fan *et al.*
ita	M	7	Wolf	wolf_Italy	Italy	PRJNA255370	SRS661489	Fan *et al.*
mxa	F	25	Wolf	wolf_Mexico	Mexico	PRJNA255370	SRS661490	Fan *et al.*
mxb	F	6	Wolf	wolf_Mexico	Mexico	PRJNA255370	SRS661491	Fan *et al.*
ptw	F	31	Wolf	wolf_Portugal	Portugal	PRJNA255370	SRS661492	Fan *et al.*
spw	F	23	Wolf	wolf_Spain	Spain	PRJNA255370	SRS661495	Fan *et al.*
pen	F	35	Wolf	wolf_Iberia	Spain	SRP073312	SRS1397562	This study
glw	M	34	Wolf	wolf_greatlake	U.S. (Great Lakes)	PRJNA255370	SRS661486	Fan *et al.*
ysa	F	28	Wolf	wolf_yellowstone	U.S. (Yellowstone)	PRJNA255370	SRS661496	Fan *et al.*
ysc	M	6	Wolf	wolf_yellowstone	U.S. (Yellowstone)	PRJNA255370	SRS661498	Fan *et al.*
Lcu2	F	9	Andean Fox	Andean Fox	Andean Fox	PRJNA232497	SRS523207	Auton *et al.*

Supplementary Table 5: Most significant *f3* test for populations with significantly negative statistics for SNP array data. Short-codes are indicated in Supplementary Table 3.

C	A	B	F3	Z score
pCH	pPO	Vie	-0.01085	-44.58
pTH	pSL	Vie	-0.00957	-43.83
I-D	pER	I-O	-0.00874	-33.63
Leb	pPO	I-O	-0.0058	-31.61
Afg	pPO	I-W	-0.00581	-28.21
I-M	I-O	Fre	-0.0088	-27.62
pMO	pPO	Vie	-0.00679	-26.91
Pap	pSL	Vie	-0.00679	-24.89
pAR	pPO	I-O	-0.0038	-24.23
Tur	pPO	I-W	-0.00454	-23.11
Mon	pAR	Vie	-0.00528	-21.92
Nep	I-W	Pap	-0.00436	-21.63
pKA	pPO	I-O	-0.00356	-21.49
pTA	pPO	I-W	-0.00427	-19.32
I-C	I-O	Hon	-0.00592	-18.7
WIN	InJ	WIR	-0.02242	-17.52
Qat	pPO	I-O	-0.00349	-10.9
WIR	WLE	WIN	-0.01895	-10.39
DRC	Hon	Nig	-0.00437	-7.25
Sol	pSL	InB	-0.00234	-7.01
I-H	I-O	InB	-0.00271	-5.49
Uga	pER	Nig	-0.00312	-5.21
I-W	I-O	InB	-0.00152	-4.91
pBU	pPO	I-O	-0.0015	-4.72
Lib	Hon	Nig	-0.00373	-4.66
pCR	pPO	Vie	-0.00099	-4.46
Bur	Lib	Nig	-0.00244	-4.17
pER	pPO	Vie	-0.00102	-3.67
Cro	pPO	I-W	-0.00101	-3.35
WIS	HXH	WIR	-0.00537	-2.26

Supplementary Table 6: Most significant *f3* test for populations with significantly negative statistics for whole genome SNP data.

C	A	B	F3	Z score
Taiwan	Qatar	Dingo	-0.01178	-24.12
sub-Saharan Africa	Europe	Basenji	-0.01136	-20.5
PNG	Lebanon	Dingo	-0.00955	-16.65
wolf_India	wolf_Israel	Jackal	-0.02024	-15.9
wolf_Iran	wolf_India	wolf_Israel	-0.01748	-11.83
Lebanon	China_MS	Basenji	-0.00585	-10.75
China South	Egypt	Dingo	-0.00383	-8.54
Vietnam	India	Dingo	-0.00325	-8.15
China North	Qatar	Dingo	-0.00296	-7.27
China Kumming	China Mongolian Shepherd	Dingo	-0.00777	-5.43
Egypt	Basenji	HXH	-0.00139	-2.62
Qatar	China Mongolian Shepherd	Basenji	-0.00189	-2.52

Supplementary Table 7: Two-way admixture events identified by `MixMapper` for HXH for both SNP array and genome data.

Data Set	Admixed Pop.	Branch1 (Br1)	Branch2	Residual norm	Br1 admixture %
Genome	HXH	Europe	Borneo	1.1E-05	0.721-0.791
SNP array	HXH	Por*	Vie	2.3E-04	0.866-1.000
SNP array	HXH	Fij*	Vie	2.3E-04	0.838-0.894
SNP array	HXH	pSL*	Vie	2.3E-04	0.865-0.873
SNP array	HXH	Bos*	InB	2.3E-04	0.836-0.906
SNP array	HXH	pPO*	InB	2.4E-04	0.845-1.000
SNP array	HXH	Por	InB*	2.4E-04	0.815-0.862
SNP array	HXH	Por	US	2.7E-04	0.688-0.716
SNP array	HXH	Por	WLE*	2.7E-04	0.827-1.000
SNP array	HXH	Bos	Vie	2.7E-04	0.836-0.876
SNP array	HXH	pSL*	InB	2.8E-04	0.858-0.873
SNP array	HXH	pPO	US	4.2E-04	0.674-1.000

Note.: * indicates admixture inferred to involve an ancestral population along the branch move in back in time from the modern representative. To simplify the table, when the same pair of modern populations occur multiple times (for example India v Europe and India v Europe*), the pair with the lowest residual norm is shown.

Supplementary Table 8: Two-way admixture events identified by `MixMapper` for NGD for both SNP array and genome data.

Data Set	Admixed Pop.	Branch1 (Br1)	Branch2	Residual norm	Br1 admixture %
Genome	NGD	Europe	Borneo	1.2E-05	0.769-0.854
SNP array	NGD	Por*	InB	2.2E-04	0.891-1.000
SNP array	NGD	pSL*	InB	2.4E-04	0.907-0.912
SNP array	NGD	pPO*	InB	2.6E-04	0.905-0.926
SNP array	NGD	pSL*	WLA	2.6E-04	0.954-1.000
SNP array	NGD	Por	Vie	2.6E-04	0.893-0.912
SNP array	NGD	Fij	Vie	2.7E-04	0.901-0.902
SNP array	NGD	Por	WLA	2.8E-04	0.944-0.961
SNP array	NGD	Bos	WLA	2.8E-04	0.949-1.000
SNP array	NGD	Bos	InB	2.8E-04	0.862-0.928
SNP array	NGD	Por	WLC	2.8E-04	0.954-1.000
SNP array	NGD	Por	WLE	2.8E-04	0.926-0.951
SNP array	NGD	pSL*	WLE*	2.9E-04	0.920-0.923
SNP array	NGD	Fij*	InB	2.9E-04	0.884-0.911
SNP array	NGD	Bos	WLE	2.9E-04	0.943-0.953
SNP array	NGD	pPO*	WLE*	3.0E-04	0.885-0.934
SNP array	NGD	pPO*	Vie	3.0E-04	0.866-0.887
SNP array	NGD	Fij	WLE	3.0E-04	0.942-0.947
SNP array	NGD	Por	US	3.3E-04	0.818-1.000
SNP array	NGD	Fij	WLA	3.3E-04	0.943-0.954
SNP array	NGD	pPO	WLA	3.7E-04	0.935-1.000
SNP array	NGD	pPO*	WLC	3.7E-04	0.940-1.000
SNP array	NGD	pPO	US	3.9E-04	0.739-0.796
SNP array	NGD	pPO	Jackal	4.1E-04	0.944-1.000
SNP array	NGD	Bos	Vie	4.2E-04	0.877-1.000

Note.: * indicates admixture inferred to involve an ancestral population along the branch move in back in time from the modern representative. To simplify the table, when the same pair of modern populations occur multiple times (for example India v Europe and India v Europe*), the pair with the lowest residual norm is shown.

Supplementary Table 9: Two-way admixture events identified by `MixMapper` for CTC for both SNP array and genome data.

Data Set	Admixed Pop.	Branch1 (Br1)	Branch2	Residual norm	Br1 admixture %
Genome	CTC	Europe	WLC	6.4E-06	0.930-1.000
Genome	CTC	Europe	14-Borneo	8.1E-06	0.803-0.876
Genome	CTC	Europe	WIS	8.7E-06	0.898-0.935
Genome	CTC	Europe	17-WLC	9.4E-06	0.929-1.000
SNP array	CTC	Ira*	WLE*	3.0E-05	0.757-1.000
SNP array	CTC	InJ*	WLE*	4.3E-05	0.712-0.735
SNP array	CTC	InJ*	InB*	4.4E-05	0.727-1.000
SNP array	CTC	pSA*	Bos	4.7E-05	0.752-1.000
SNP array	CTC	Ira*	WLE*	5.8E-05	0.780-0.856
SNP array	CTC	pIR*	WLE*	5.9E-05	0.790-1.000
SNP array	CTC	I-O*	InJ*	6.1E-05	0.509-1.000
SNP array	CTC	pSA*	Por	7.1E-05	0.741-0.784
SNP array	CTC	pSA*	pPO	7.9E-05	0.774-1.000
SNP array	CTC	I-O*	Bos*	9.5E-05	0.567-1.000

Note.: * indicates admixture inferred to involve an ancestral population along the branch move in back in time from the modern representative. To simplify the table, when the same pair of modern populations occur multiple times (for example India v Europe and India v Europe*), the pair with the lowest residual norm is shown.

Supplementary Table 10: *f4*-ratio test on the form of the ratio between f4(A,O; X,C) and f4(A,O; B, C).

A	B	X	C	O	alpha	std.err	Z
Indian	Portugal	HXH	South China	Andean fox	0.7561	0.0341	22.175
Indian	Portugal	HXH	Vietnam	Andean fox	0.7778	0.0313	24.840
Indian	Portugal	NGD	South China	Andean fox	0.7340	0.0369	19.907
Indian	Portugal	NGD	Vietnam	Andean fox	0.7576	0.0337	22.483
Basenji	India	CTC	Portugal	Andean fox	-1.2248	0.4393	-2.7880
Basenji	India	CTC	HXH	Andean fox	0.1852	0.0977	1.8960
Lebanon	Portugal	CTC	India	Andean fox	0.1816	0.0369	4.9220
Lebanon	HXH	CTC	India	Andean fox	0.2436	0.0489	4.9870
Portugal	HXH	CTC	India	Andean fox	0.3083	0.0402	7.6630
Vietnam	CTC	wolf_India	wolf_Iran	Andean fox	-0.0106	0.0095	-1.1110
Vietnam	Portugal	wolf_India	wolf_Iran	Andean fox	-0.0099	0.0089	-1.1110

Supplementary Table 11: Three-way admixture events identified by `MixMapper` for CTC for both SNP array and genome data.

Data Set	Admixed Population	Mixed Population 1	Branch3	Residual norm	Branch 3 admixture %
SNP array	CTC	HXH	I-O*	9.7E-05	0.220-0.225
SNP array	CTC	HXH	pSA*	1.0E-04	0.125-0.264
Genome	CTC	HXH	14-Borneo	1.8E-05	0.722-0.832
Genome	CTC	HXH	13-India	2.0E-05	0.139-0.372
Genome	CTC	HXH	WIS	2.2E-05	0.877-0.906

Note: * indicates admixture inferred to involve an ancestral population along the branch move in back in time from the modern representative.

Supplementary Table 12: *f4* statistics of the form F4(ancient,ancient, Wolf,AndeanFox). Significant values in bold.

Pop A	Pop B	Wolf	Outgroup	D-statistic	Z-score
HXH	CTC	ChineseWolf	AndeanFox	0.0102	2.32
HXH	CTC	CroatianWolf	AndeanFox	0.0034	0.778
HXH	CTC	IberianWolf	AndeanFox	0.0006	0.139
HXH	CTC	IndianWolf	AndeanFox	-0.0042	-0.966
HXH	CTC	IranianWolf	AndeanFox	-0.0053	-1.303
HXH	CTC	IsraeliWolf	AndeanFox	0.0052	1.087
HXH	CTC	ItalianWolf	AndeanFox	0.0014	0.279
HXH	CTC	PortugeseWolf	AndeanFox	0.0006	0.132
**HXH**	**CTC**	**SpanishWolf**	**AndeanFox**	**0.0219**	**4.214**
HXH	NGD	ChineseWolf	AndeanFox	0.0017	0.396
HXH	NGD	CroatianWolf	AndeanFox	-0.0011	-0.26
HXH	NGD	IberianWolf	AndeanFox	-0.0059	-1.222
HXH	NGD	IndianWolf	AndeanFox	-0.0017	-0.391
HXH	NGD	IranianWolf	AndeanFox	-0.0023	-0.515
HXH	NGD	IsraeliWolf	AndeanFox	0.0029	0.596
HXH	NGD	ItalianWolf	AndeanFox	-0.0006	-0.123
HXH	NGD	PortugeseWolf	AndeanFox	-0.0117	-2.332
HXH	NGD	SpanishWolf	AndeanFox	-0.0034	-0.678
NGD	CTC	ChineseWolf	AndeanFox	0.0086	1.897
NGD	CTC	CroatianWolf	AndeanFox	0.0043	0.973
NGD	CTC	IberianWolf	AndeanFox	0.0058	1.369
NGD	CTC	IndianWolf	AndeanFox	-0.0026	-0.622
NGD	CTC	IranianWolf	AndeanFox	-0.0032	-0.793
NGD	CTC	IsraeliWolf	AndeanFox	0.0026	0.568
NGD	CTC	ItalianWolf	AndeanFox	0.0019	0.386
NGD	CTC	PortugeseWolf	AndeanFox	0.0109	2.406
**NGD**	**CTC**	**SpanishWolf**	**AndeanFox**	**0.0246**	**4.619**

Supplementary Table 13: Two-way admixture events identified by `MixMapper` for CTC and Afg for SNP array data when including HXH as a scaffold population.

Admixed Population	Branch1	Branch2	Residual norm	Admixture %
CTC	I-O*	HXH	1.1E-04	0.483-0.601
CTC	pSA*	HXH	1.0E-04	0.601-0.770
CTC	HXH	WLE*	1.1E-04	0.640-0.725
CTC	HXH	InB*	1.1E-04	0.613-0.706
CTC	Pal*	HXH	2.2E-04	0.787-0.852
CTC	HXH	Jackal	1.6E-04	0.947-1.000
CTC	HXH	WLE	1.8E-04	0.927-0.934
CTC	HXH	WLC	1.7E-04	0.940-0.947
Afg	pSA*	Bos	4.5E-05	0.702-0.764
Afg	pSA*	Por	4.6E-05	0.716-0.768
Afg	pSA*	HXH	4.4E-05	0.660-1.000
Afg	Pal*	I-O	5.4E-05	0.769-0.835
Afg	InJ*	I-O	6.7E-05	0.904-0.930
Afg	Ira*	I-O	6.2E-05	0.907-1.000
Afg	HXH*	I-O	7.0E-05	0.881-0.899
Afg	Bos*	I-O	7.5E-05	0.884-0.905
Afg	Ira*	I-O	5.7E-05	0.840-1.000
Afg	Ira*	Vie	7.6E-05	0.959-1.000
Afg	Ira*	InB*	6.9E-05	0.925-0.936
Afg	pSA*	Bos*	4.9E-05	0.684-1.000

Supplementary Table 14: Outlier *f4* statistics for our best admixture graph fitting HXH, CTC, NGD, modern dogs and wolves.

Pop W	Pop X	Pop Y	Pop Z	Fitted *f*	Estimated *f*	Difference	std.err	Z_score
Andean Fox	wolf_China	Europe	HXH	2.6E-04	2.6E-03	2.3E-03	7.5E-04	3.07
Andean Fox	wolf_China	India	HXH	-1.2E-04	3.5E-03	3.6E-03	7.6E-04	4.73
Andean Fox	wolf_China	India	NGD	-1.9E-04	2.8E-03	3.0E-03	7.9E-04	3.80
Bornean	India	HXH	NGD	3.2E-03	2.8E-04	-2.9E-03	8.3E-04	-3.51

Supplementary Table 15: Parameter estimates in G-PhoCS analysis. We used the tree of the form (((((boxer, Village_Europe), Village_India), Village_ChinaS),((wolfY,IsraeliWolf), ChineseWolf)),GoldenJackal). Divergence time and population size estimates are scaled up by 1x10^{-4}. When using Croatian wolf, we report the result using 16,434 loci. When using Indian wolf, we report the result using 5,000 randomly selected loci.

	**Y=Croatian**		**Y=Indian**		**Dingo (instead of China_S), Y=Croatian**	
**Divergence time**	**mean**	**95% CI**	**mean**	**95% CI**	**mean**	**95% CI**
Box, Europe	0.0853	(0.0576-0.1239)	0.1360	0.07136-0.2036	0.0774	(0.0444-0.1019)
(Box, Europe),Indian	0.2137	(0.1825-0.2386)	0.2111	0.1674-0.2472	0.1683	(0.1425-0.2011)
((Box, Europe),Indian), China_S	0.2786	(0.2329-0.3184)	0.2545	0.2210-0.2863	0.5106	(0.4913-0.5348)
(Y,IsraeliWolf)	0.5234	(0.4912-0.5522)	0.4856	0.4351-0.5402	0.5336	(0.5104-0.5535)
(Y,IsraeliWolf),Chinese Wolf	0.5237	(0.4915-0.5526)	0.4893	0.4369-0.5468	0.534	(0.5109-0.5538)
(Dog,Wolf)	0.5247	(0.4926-0.5530)	0.4910	0.4384-0.5509	0.539	(0.5177-0.5583)
**Effective population size**	**mean**	**95% CI**	**mean**	**95% CI**	**mean**	**95% CI**
Box	0.2357	(0.1591-0.3369)	0.4217	0.2256-0.6329	0.2357	(0.1591-0.3369)
Europe	6.6661	(3.9935-10.2473)	5.8360	3.2223-9.0717	6.6661	(3.9935-10.2473)
Indian	1.3976	(1.1608-1.6241)	1.4559	1.1061-1.8082	1.3976	(1.1608-1.6241)
China_S	1.8453	(1.5546-2.1317)	1.5275	1.2452-1.8169	1.8453	(1.5546-2.1317)
ChineseWolf	3.1825	(2.9587-3.4114)	3.2977	2.9318-3.6799	3.1825	(2.9587-3.4114)
IsraeliWolf	20.4963	(17.3494-24.0356)	16.1577	12.7658-20.1083	20.4963	(17.3494-24.0356)
wolfY	6.9477	(6.3080-7.6399)	17.8693	14.1961-22.2976	6.9477	(6.3080-7.6399)
GoldenJackal	9.0472	(8.7730-9.3254)	9.4533	8.9287-9.9921	9.0472	(8.7730-9.3254)
Anc(Box, Europe)	1.4153	(0.8569-1.9452)	1.0577	0.2244-2.0301	1.4153	(0.8569-1.9452)
Anc((Box, Europe),Indian)	1.3602	(0.2464-2.3413)	1.1013	0.3578-2.4864	1.3602	(0.2464-2.3413)
Anc(Dog)	1.5643	(1.1890-2.0036)	1.5958	1.1872-2.0221	1.5643	(1.1890-2.0036)
Anc(Wolf1)	0.0216	(0.0143-0.0313)	0.1455	0.0104-0.3871	0.0216	(0.0143-0.0313)
Anc(Wolf)	1.2131	(0.0658-3.7675)	1.6395	0.1598-4.2191	1.2131	(0.0658-3.7675)
Anc(DW)	17.8534	(17.5605-18.1572)	18.0963	17.6312-18.5731	17.8534	(17.5605-18.1572)
**Total Migration rate**	**mean**	**95% CI**	**mean**	**95% CI**	**mean**	**95% CI**

Box->ISW	0.0003	(0.0000-0.0009)	0.0107	0.0006-0.0291	0.0016	(0.0000-0.0081)
ISW->Box	0.0823	(0.0540-0.1174)	0.0446	0.0243-0.0783	0.0682	(0.0461-0.1133)
Europe->ISW	0.0472	(0.0303-0.0693)	0.0000	0.0000-0.0000	0.0366	(0.0221-0.0600)
ISW->Europe	0.072	(0.0472-0.1025)	0.0392	0.0203-0.0685	0.0704	(0.0475-0.1174)
Indian->ISW	0.0116	(0.0000-0.0475)	0.1591	0.0739-0.2552	0.0125	(0.0001-0.0472)
ISW->Indian	0.4729	(0.3747-0.5910)	0.0188	0.0001-0.1030	0.6741	(0.5417-0.8132)
China_S->CHW	0.0016	(0.0001-0.0068)	0.0059	0.0000-0.0259	0.0142	(0.0011-0.0272)
CHW->China_S	0.0299	(0.0165-0.0437)	0.0276	0.0034-0.0570	0.016	(0.0024-0.0313)
ISW->GLJ	0.0629	(0.0515-0.0737)	0.0599	0.0430-0.0762	0.0621	(0.0506-0.0735)
GLJ->ISW	0.0065	(0.0044-0.0087)	0.0000	0.0000-0.0000	0.0069	(0.0049-0.0090)
AncDW->GLJ	0.7149	(0.6424-0.7955)	0.5693	0.4400-0.7002	0.7413	(0.6655-0.8246)
GLJ->AncDW	0.0208	(0.0134-0.0285)	0.0509	0.0356-0.0674	0.0156	(0.0099-0.0223)
Indian->IDW	-	-	0.0280	0.0001-0.1028		
IDW->India	-	-	0.3414	0.2230-0.4802		

Supplementary Table 16: Comparison of divergence time estimates from previous studies using G-PhoCS

	dog/wolf divergence (kyrs)		Dog divergence(kyrs)	
Studies	μ=1x10^{-8}	μ=4x10^{-9}	μ=1x10^{-8}	μ=4x10^{-9}
Freedman *et al.*	15.00	37.50	12.90	32.25
Wang *et al.*	24.60	61.50	9.60	24.00
Fan *et al.*	11.70	29.25	-	-

Note, μ is in units of per base per generation, assuming a generation time of 3 years

Supplementary Table 17: Jackknife estimates and confidence interval of the ratio of the number of SNPs. Estimates where i) a European village dog and dogX have the derived allele and an Indian sample has the ancestral allele versus ii) a European and Indian village dog have the derived allele and dogX has the ancestral allele.

dogX	Jackknife Estimates	Standard Deviation	Confidence Interval
Boxer	1.533886	0.020438	1.513448-1.554324
HXH	1.201485	0.015546	1.185939-1.217031
NGD	1.212794	0.017810	1.194984-1.230604

Supplementary Table 18: CanFam3.1 coordinates, IDs, and length of putative domestication loci first defined in Axelsson *et al.*. For each window, the number of SNPs from our dataset with and without minor allele frequency filtration is provided.

Chrom.	Start Position	End Position	Window ID	Window Length (bp)	No. SNPs (Unfiltered)	No. SNPs (0.05 < MAF < 0.49)
chr1	2515609	3315787	FST_1	800178	1506	492
chr1	46572801	46874590	FST_2	301789	483	158
chr1	63560427	63760428	FST_3	200001	680	234
chr1	79948274	80148387	FST_4	200113	559	180
chr3	15326158	15626159	FST_6	300001	429	133
chr3	18623349	18823350	FST_7	200001	371	116
chr3	32064283	32261994	FST_8	197711	348	115
chr4	14494337	14694347	FST_9	200010	638	183
chr4	40803259	41001116	FST_10	197857	269	111
chr6	24980113	25280253	FST_11	300140	743	236
chr6	46854109	47454177	FST_12	600068	1231	540
chr6	53253174	53453184	FST_13	200010	437	124
chr7	24632212	25033464	FST_14	401252	456	114
chr8	27696699	27896700	FST_15	200001	655	244
chr10	2714704	2914705	FST_16	200001	364	89
chr10	3615192	4015195	FST_17	400003	891	306
chr11	47269655	47669725	FST_19	400070	1037	371
chr14	7244540	7543124	FST_20	298584	373	115
chr15	5093547	5393613	FST_21	300066	311	83
chr15	35192648	35392649	FST_22	200001	338	120
chr16	6828779	7342805	FST_23	514026	1448	627
chr17	38657285	38857286	FST_24	200001	528	196
chr18	414503	1414626	FST_25	1000123	2415	649
chr18	3217604	4718037	FST_26	1500433	3533	1118
chr19	37683164	37883157	FST_27	199993	481	184
chr22	19995752	20192912	FST_29	197160	348	151
chr25	1015698	1515729	FST_30	500031	816	282
chr28	6403026	6603027	FST_32	200001	288	84
chr28	9202946	9502947	FST_33	300001	694	224
chr37	6915094	7115095	FST_35	200001	428	168

Supplementary Table 19: Proportion of dogs and outgroups (wolves, jackal, coyotes, and fox) that exhibit reference (breed dog) allele proportions over 0.95. Windows with less than 0.25 of dogs and greater than 0.75 of outgroup proportions are considered as passing. Reference allele proportions per window are provided for HXH, CTC, and NGD. Ancient sample proportions < 0.95 are indicated in bold font (i.e. exhibiting wild-like genotypes). Window marked with asterisk represents data from less variable subset of selection window.

FST Window Identifier	PASS/FAIL	Window SNP Count	Proportion of Dogs with Ref. Allele Counts Over Threshold	Proportion of Wolves + Outgroups with Ref. Allele Counts Over Threshold	HXH Ref. Allele Proportion	CTC Ref. Allele Proportion	NGD Ref. Allele Proportion
**FST_1**	PASS	1506	0.025	1.000	0.989	0.993	0.984
**FST_2**	PASS	483	0.288	1.000	0.990	0.995	0.994
**FST_3**	PASS	680	0.213	1.000	0.980	0.988	0.999
**FST_4**	PASS	559	0.050	1.000	0.997	**0.902**	0.997
**FST_6**	FAIL	429	0.350	1.000	0.992	0.972	0.962
**FST_7**	FAIL	371	0.250	1.000	0.991	0.992	0.911
**FST_8**	FAIL	348	0.388	0.895	0.970	0.986	0.977
**FST_9**	PASS	638	0.125	0.947	0.969	0.972	0.991
**FST_10**	PASS	269	0.125	1.000	0.994	**0.918**	0.996
**FST_11**	PASS	743	0.175	1.000	0.994	0.996	0.999
**FST_12**	PASS	1231	0.238	1.000	0.956	**0.850**	**0.851**
**FST_13**	PASS	437	0.138	1.000	**0.932**	0.995	1.000
**FST_14**	FAIL	456	0.400	1.000	0.891	0.935	0.876
**FST_15**	FAIL	655	0.388	1.000	0.969	0.989	0.976
**FST_16**	FAIL	364	0.313	0.737	0.985	0.973	0.992
**FST_17**	PASS	891	0.088	1.000	0.993	**0.921**	0.998
**FST_19**	PASS	1037	0.163	0.947	0.976	0.982	0.993
**FST_20**	PASS	373	0.313	1.000	0.981	0.976	**0.899**
**FST_21**	PASS	311	0.063	0.895	0.994	**0.875**	0.994
**FST_22**	FAIL	338	0.438	1.000	0.991	0.947	0.997
**FST_23***	PASS	1448	0.438	1.000	0.994	0.996	0.997
**FST_24**	FAIL	528	0.588	1.000	0.976	0.951	0.975
**FST_25**	PASS	2415	0.038	1.000	0.992	0.992	0.998
**FST_26**	PASS	3533	0.138	1.000	0.983	0.993	0.997
**FST_27**	FAIL	481	0.688	1.000	0.868	0.963	0.919
**FST_29**	FAIL	348	0.788	1.000	0.974	0.902	0.934
**FST_30**	FAIL	816	0.263	1.000	0.991	0.989	0.996
**FST_32**	FAIL	288	0.438	1.000	0.995	0.998	0.938
**FST_33**	PASS	694	0.088	1.000	0.986	**0.891**	0.996
**FST_35**	PASS	428	0.138	1.000	0.979	0.986	0.996

Supplementary Table 20: Miseq results for HXH.

Sample	Raw fastq reads	Trimmed fastq reads	Aligned reads to CanFam3.1	Reads following samtools rmdup	Aligned Reads following rmdup	Aligned following rmdup (%)	Aligned Reads following QC (-q30)	Aligned following QC (%)
HXH	322,579	318,759	193,007	318,543	192,791	60.52	151,978	47.68

Supplementary Table 21: MiSeq results of controls.

Control	Raw fastq reads	Trimmed fastq reads	Aligned reads to CanFam3	Reads following samtools rmdup	Aligned Reads following rmdup	Aligned following rmdup (%)	Aligned Reads following QC (-q30)	Aligned following QC (%)
Air Control	467	396	3	396	3	0.76	3	0.76
Water Control	578	523	3	523	3	0.57	1	0.19
Extraction Control 1	886	786	18	786	18	2.29	2	0.25
Extraction Control 2	552	512	3	512	3	0.59	2	0.39
Library Control 1	155	148	0	148	0	0.00	0	0.00
Library Control 2	848	540	2	540	2	0.37	2	0.37
PCR Control 1	116	115	1	115	1	0.87	1	0.87
PCR Control 2	635	610	0	610	0	0.00	0	0.00

Supplementary Table 22: Qubit measurements of all sequenced libraries and corresponding blank controls of CTC.

**Extraction_sample or blank**	**Extraction.Library**	**Qubit (ng/µl)**
1_Kir20	1.1	5.83
1_milling blank	1	0.20
1_extraction blank	1	0.17
1_library blank		0.17
2_Kir20	2.1	5.43
2_Kir20	2.2	14.3
2_Kir20	2.3	8.99
2_extraction blank	2	0.47

Supplementary Table 23: The range of parameters we sampled. *θ* and *τ* estimates were based on `G-PhoCS` results and alpha value was based on *f4*-ratio analysis.

Parameter	Uniform range
θ_1	(0.86 x 10^{-4}, 1.95 x 10^{-4})
θ_2	(0.25 x 10^{-4}, 2.34 x 10^{-4})
τ_0	(5.76 x 10^{-6}, 1.24 x 10^{-5})
τ_2	(1.83 x 10^{-5}, 2.39 x 10^{-5})
τ_3	(2.33 x 10^{-5}, 3.18 x 10^{-5})
α	(0.19,0.28) for HXH<br>(0.20,0.30) for NGD<br>0 for Boxer

Supplementary Table 24: D-statistics supporting the migration band setting used in G-PhoCS.

Pop A	Pop B	Pop C	Outgroup	f4	Z-score
wolf_Israeli	wolf_China	Boxer	Andean fox	0.0389	6.628
wolf_Israeli	wolf_Croatia	Boxer	Andean fox	0.0368	6.390
wolf_China	wolf_Croatia	South China	Andean fox	0.0139	4.079
wolf_Israeli	wolf_China	India	Andean fox	0.0366	7.474
wolf_Israeli	wolf_Croatia	India	Andean fox	0.0355	7.278
wolf_Israeli	wolf_India	India	Andean fox	0.0600	11.115
wolf_Israeli	wolf_China	Portugal	Andean fox	0.0378	6.744
wolf_Israeli	wolf_Croatia	Portugal	Andean fox	0.0363	6.602
Boxer	South China	wolf_China	Andean fox	-0.0129	-3.890
India	South China	wolf_China	Andean fox	-0.0170	-7.902
Portugal	South China	wolf_China	Andean fox	-0.0143	-5.187
Boxer	South China	wolf_Israeli	Andean fox	0.0342	9.086
India	South China	wolf_Israeli	Andean fox	0.0258	10.603
Portugal	South China	wolf_Israeli	Andean fox	0.0311	9.812

Supplementary Note 1: Archaeological background

Herxheim (HXH)

The internal ditch structure of the Herxheim site contains a significant amount of faunal remains that are mainly characterized by an important representation of dogs. The >250 remains of this species constitute the largest bone series of Early Neolithic dogs in western Europe. The pottery associated with the human and animal bones in this concentration date to the youngest phase of the Linear Pottery Culture (LBK), of which all the finds in the settlement ditches belong.

The dog bone series belong to all skeletal parts but with an over-representation of cranial fragments compared to rachis and limbs. Mandibles, which correspond to the best represented skeletal remains, attest the presence of at least 13 individuals. The distribution of the dog bones within the pit is characterized by concentrations of anatomically related elements (skull and cervical vertebrae, sections of spine and limb fragments). The bones are notably quite complete, and carry both burn and meat carving marks. This suggests that the animals were prepared by various procedures for consumption (i.e. carving in quarters, cooking by fire roasting). The dog remains were found mixed with remains from other animals and with abundant human remains. The latter were systematically and extensively fragmented and exhibit numerous marks of dislocation, meat carving and scraping, which are typical for cannibalism[1].

The sample used in this study, named HXH from this point on, (Supplementary Figure 1) belongs to a concentration of findings (K 3, rescue excavation) from the southern part of the inner ditch. Carbon dating estimated a date of 5,223-5,040 BCE cal. Only a single petrous bone from this dog was available. However, the biometrics of dog remains at the site in general (width and length of bones and teeth) are clearly distinct, with smaller values compared to those of wolves from the same Neolithic period. Namely, the modest sizes of the first molars are within the range of those of other Neolithic dogs rather than of wolves. Measurements of available long bones, which allow estimation of a height, provide values of around 46 cm. These values are in agreement with dogs from other Early Neolithic sites, but are somewhat larger than values from more recent Neolithic sites [2].

Kirschbaumhöhle (CTC)

The Kirschbaumhöhle (Cherry Tree Cave), situated near Forchheim in the karst landscape of Northern Franconian low mountain range, was discovered in November 2010 [3]. This quite small-scale vertical cave is divided into several parts: the vertical Entry Shaft, the steep Descent Tube, the very low and fissured Bone Chamber, located about 6 m below ground level, and the more accessible Sinter Chamber.

Visible finds lying on the surface of the cave consisted of six human skulls, other human bones and the remains of domestic and wild animals. Terrestrial 3D scanning was used to obtain the exact position of each bone. All work inside the cave was performed with protective clothing to avoid contaminating the prehistoric bones with modern DNA. In all, 188 bones with a weight of about 10 kg have been recovered thus far. A large series of radiocarbon dates demonstrate that the human and animal remains belong to at least six prehistoric periods: the Younger Neolithic (presumably Michelsberg Culture) between 3,800 and 3,650 BCE cal., the early End Neolithic

(presumably Corded Ware Culture) between 2,900 and 2,630 BCE cal., the middle End Neolithic (Corded Ware Culture or Bell Beaker Culture) between 2,580 and 2,460 BCE cal., the late End Neolithic (Corded Ware Culture or Bell Beaker Culture) between 2,340 and 2,140 BCE cal., the Early Bronze Age between 1,800 and 1,700 BCE cal. and the middle Iron Age (Late Hallstatt/Early Latène) between 580 and 420 BCE cal.

The dog cranium examined in this study (from this point on referred to as CTC) (Supplementary Figure 2) was found in the Bone Chamber near two human skulls which dated to the early End Neolithic period (Supplementary Figure 27), presumably connected with the Corded Ware Culture. The radiocarbon date of the dog skull coincides with them and reveals that it got into the cave between 2,900 and 2,632 BCE cal. CTC was adult and by the state of tooth abrasion, older than 5 years. The measurements of the skull show a similarity to the so-called Torfhund (*Canis familiaris palustris*) which is comparable to the modern Spitz breed [4]. This dog type is also known from lakeside settlements of Corded Ware Culture in Switzerland [5].

Supplementary Note 2: DNA Isolation and screening of HXH

Sample preparation

Note that in initial stages of the project sample HXH had the identifier HXH10a. The petrous part of the temporal bone of sample HXH was prepared in clean-room facilities dedicated to ancient DNA at Trinity College Dublin (Ireland). Researchers wore body suits, face masks, gloves and shoe covers to minimize potential contamination from outside. Surfaces and tools were cleaned using a 5% bleach solution at regular intervals.

The bone sample was decontaminated using UV exposure for 15 minutes on both sides, followed by surface cleaning with a standard dentist drill and drill bit. A sample of the bone was then excised using a dremel engraving cutter (saw), and which was reduced to a fine powder using a Mixer Mill (MM 400, Retsch). Two environmental controls (an air control and a water control) were also prepared, to estimate the possible contaminants in the working environment.

DNA extraction

Approximately 120 mg of powdered bone was used in DNA extraction. The protocol followed was introduced by [6] and subsequently modified by [7]. Two tubes each containing 1 ml H_2O were included as extraction controls, and subjected to identical treatment as bone powder samples.

One ml of lysis buffer (1M Tris-HCl; 2% SDS; 0.5M EDTA; 0.65 U/ml Proteinase K) was prepared and transferred to sample tubes, which were incubated in a thermocycler for 24 hours at 37□. Samples were then spun down at 13,300 rpm and the lysis buffer removed. 1 ml fresh extraction buffer was added and sample tubes incubated again under the same conditions. This was repeated again for a total of three extractions. After spindown of the final extraction, the supernatant was transferred to Amicon filters (Amicon Ultra-4 Centrifugal Filter Unit 30 kDA) with 3 ml 1x Tris-EDTA was added to each filter. Samples were then spun down to the 250 µl mark, and the flow-through discarded. Fresh 3 ml 1x Tris-EDTA was added and tubes spun down to the 100 µl mark. This final 100 µl was then transferred to a MinElute Silica column (MinElute PCR Purification Kit, Qiagen, Hilden, Germany). Purification was completed as per the manufacturer's instructions. DNA was eluted in 40 µl EBT (Elution Buffer with 0.05% Tween).

Library preparation

Two Next Generation Sequencing (NGS) libraries were constructed using 16.25 µl of DNA extract each. Libraries were constructed as described in [8] with modifications from [9]. Two blanks of 16.25 µl H_20 were included.

Briefly, NEB Next End Repair Module (New England BioLab Inc.) with reagent volumes scaled to 70% (final reaction volume 70 µl) was used to perform blunt-end repair. The reaction mix was incubated for 15 minutes at 25□, then 5 minutes at 5□. This was followed by Qiagen MinElute PCR purification following manufacturer's instructions and elution in 20 µl EBT. Adaptor ligation using T4 DNA ligase was performed on the elute, followed by MinElute purification as described previously. The elute was then subjected to adaptor fill-in, with the addition of a heat

inactivation step (20 minutes at 80□) in place of additional purification.

Amplification was performed using 3 µl fill-in product, 1 µl of a unique index oligo (5 µM) and 21 µl of amplification master mix composed of 20.5 µl AccuPrime Pfx Polymerase (Invitrogen) and 0.5 µl primer IS4 (10 µM). Amplification was performed for 13 cycles for a single PCR used for MiSeq screening, and for 12 cycles for an additional 19 PCRs used for HiSeq sequencing in a dedicated ancient DNA PCR room (95°C for 5 min; 11-12 x 95°C for 15 sec, 60°C for 30 sec, 68°C for 30 sec; 68°C for 5 min). Two blank controls (3 µl H_2O) were included. Following amplification, all individual PCRs were purified using MinElute columns as described above and eluted in 10 µl EB. The 19 PCRs used for HiSeq sequencing were pooled equimolarly and then re-purified with Agencourt® AMPure® XP beads (Beckmann Coulter) to reduce primer dimers. DNA quantification was performed using an Agilent 2200 Tapestation using a D1000 ScreenTape (Agilent Technologies) and Qubit® Fluorometric quantitation (dsDNA HS assay, Invitrogen).

MiSeq screening

20 ng of the purified PCR product for HXH were included on a pooled sequencing run with spiked-in control libraries (1 µl) on an Illumina MiSeq platform (Trinity Genome Sequencing Laboratory, Trinity College Dublin, Ireland) using 70 bp single-end sequencing. The resulting reads were trimmed using `Cutadapt V1.6`[10], discarding reads less than 30 bp in length and allowing for a minimum length of 1 between the read and adaptor (cutadapt -a AGATCGGAAGAGCACACGTCTGAACTCCAGTCAC -O 1 -m 30). Reads were then aligned to a collection of genomes using `bowtie2`[11] and `fastq screen` (Babraham Bioinformatics) to determine species identity. Based on affinity of reads to the *Canis familiaris* reference genome in the fastq screening (Supplementary Figure 28), trimmed fastq files were aligned to CanFam3.1 using `bwa aln`[12] with seeding disabled (`bwa aln -l 1000`). After duplicate removal, length and quality filtering, 47.68% of reads mapped the dog genome (Supplementary Table 20).

Blank controls

Prepared control libraries (environment, extraction, library and PCR) were quantitatively analyzed with the Tapestation 2200 and did not display substantial levels of DNA (Supplementary Figure 29). Signals at approximately the 60 bp range in control libraries likely reflects index dimerization.

Control libraries were included in a pooled MiSeq sequencing run and were aligned to CanFam3 to directly estimate potential contamination. The number of reads aligning to CanFam3 following QC for each control library was small (< 1%; Supplementary Table 21) relative to the those aligning in the HXH library.

Supplementary Note 3: DNA Isolation and screening of CTC

Sample preparation

Note that in initial stages of the project sample CTC had the identifier Kir20. Sample preparation was conducted in dedicated ancient DNA facilities of the Palaeogenetics Group at Johannes Gutenberg-University Mainz under strict rules for contamination prevention as described in Bramanti *et al.* [13]. The petrous bone was extracted from the skull using a rotary saw (Electer Emax IH-300, MAFRA) and UV-irradiated for 45 min from two sides. The surface and spongious parts were mechanically removed using a sandblaster (P-G 400, Harnisch & Rieth). The most dense parts remaining of the petrous bone were again UV-irradiated for 45 min from each side. The sample was then pulverised using a mixer mill (MM200, Retsch).

DNA extraction

DNA was independently extracted twice. For the first extraction, 0.3 g of bone powder was incubated for 45 min at 37°C on a rocking shaker in a decalcification and digestion solution containing 1 ml of EDTA (0.5 M, pH8; Ambion®, Applied Biosystems), 250 µl of N-Laurylsarcosine (0.5 %, Merck) and 30 µl of Proteinase K (18 U, Roche). The sample was spun down and the supernatant removed. For the second extraction, 0.5 g of bone powder was used and the first round of incubation was completed in 30 min. The remaining bone powder of both samples was again incubated in 6 ml of EDTA, 250µl of N-Laurylsarcosine and 30 µl of Proteinase K on a rocking shaker for two days at 37°C. Further steps were performed according to Scheu *et al.* [14] on the basis of a phenol-chloroform protocol with subsequent washing and concentration to 200 µl of extract using 50 kDA 50 ml Amicons (Millipore).

Library preparation

50 µl of the first extract were transformed into one NGS library (library 1.1), and 60 µl of the second extract were transformed into three libraries (2.1, 2.2 and 2.3; 20 µl of extract diluted with 30 µl of water each). Library preparation followed the protocol in Meyer and Kircher [8] with slight modifications as described in Hofmanová *et al.* [15]. Double indexing was performed according to Kircher *et al.* [16] using the index sequences from Illumina's NexteraXT Kit v2.

Libraries were amplified in 3 parallel PCRs using AmpliTaq Gold® DNA polymerase (Applied Biosystems) and 16 cycles for library 1.1, and 12 cycles for libraries 2.1, 2.2 and 2.3.
The amplified library 1.1 was purified using the Stratec MSB® Spin PCRapace kit (Stratec Biomedical AG), and all 3 PCRs were eluted together in 34 µl of EB. The 9 PCRs of libraries 2.1, 2.2 and 2.3 were purified with Agencourt® AMPure® XP beads (Beckmann Coulter) and all 3 per library eluted together in 12 µl of EB. The resulting 4 samples were subsequently quantified with both, Qubit® Fluorometric quantitation (dsDNA HS assay, Invitrogen) and Agilent 2100 Bioanalyzer System (HS, Agilent Technologies).

MiSeq screening

The amplified library of the first extract was sequenced in a pool with other libraries from different projects on the Miseq at StarSEQ GmbH (Mainz, Germany). The resulting 1,321,372 50 bp single-end reads were processed using the pipeline described in Hofmanová *et al.* [15] . The

reads were aligned against the dog reference genome CanFam3 with the mitochondrial genome replaced by GenBank U96639. After duplicate removal, length and quality filtering, 61.5% of reads mapped the dog genome.

Blank controls

Blank controls were processed during milling (as described in Scheu *et al.* [14]), extraction and library preparation. Amplified libraries of the blank controls were quantified with Qubit® Fluorometric quantitation (dsDNA HS assay, Invitrogen) and Agilent 2100 Bioanalyzer System (HS, Agilent Technologies). None of the blank controls showed signs for amplification at the respective fragment lengths of the samples that were processed in parallel, and the Qubit measurements revealed on average 94% lower values in the blanks compared to the sample (see Supplementary Table 22). The remaining measured molecules are most likely reflecting the dimer peaks visible in the Bioanalyzer measurements of the blank controls (Supplementary Figure 30).

Supplementary Note 4: HiSeq Sequencing and Read Processing

HXH and CTC

Combinations of various genomic libraries from each ancient sample (CTC and HXH) were sequenced on two lanes of an Illumina HiSeq 2500 1TB at the New York Genome Center (NYGC) using the High Output Run mode to produce 2x125 paired end reads (Supplementary Table 2). For sample CTC, library 1.1 and an equimolar pool of libraries 2.1, 2.2 and 2.3 (Supplementary Table 22) were sequenced on one lane each. For sample HXH, an equimolar pool of 19 PCRs from two libraries were sequenced on two lanes. Fastq files from individual libraries were demultiplexed by the NYGC prior to delivery.

We largely followed the ancient DNA pipeline of Kircher [17] to process our read data, though slight modifications were made to scripts to accommodate our data. The first 50bp of each R1 read was trimmed using the `TrimFastQ.py` script. `TagDust` [18] was then used to identify potential library artifact sequences based on known Illumina adaptor sequences. The thirty most frequent artifacts for each sample were identified and a pairwise alignment was constructed for each against the adaptor sequences. Pairwise alignments were then manually inspected for evidence of sequence motifs that likely represent artifacts such as forward and reverse adaptors containing no or very small inserts. This list of motifs was used as input for the *`MergeReadsFastQ_cc.py`* script in order to merge paired-end reads with substantial sequence overlap into single reads. Any remaining paired-end reads were discarded. Finally, reads with at least 5 base calls with a Phred-scaled quality score of less than 15 were removed using the `QualityFilterFastQ_gz.py` script.

Reads were mapped to the CanFam3.1 reference genome using `BWA aln` [12] using the following parameters: maximum edit distance=1%, number of gap opens=2, l=16500. Read groups were added using the `PICARD` tool `AddOrReplaceReadGroups` and duplicate reads were removed using `MarkDuplicates`. Finally, InDels were realigned using the `GATK RealignerTargetCreator` and `IndelRealigner` tools [19] to produce a finished BAM file for each sample.

The proportion of reads mapping to the reference genome was >67% for both samples (Supplementary Table 2), confirming high endogenous DNA content identified during screening. `MapDamage` [20] analysis demonstrated that both samples possessed characteristics typical of ancient DNA [21] such as high numbers of 5’ C>T and 3’ G>A changes at the end of fragments (Supplementary Figure 3) (~35% and 28% of each transition category for HXH and CTC, respectively), while fragment length was also small (mean 60-70bp). When examining a subset of ~1 million random reads, only ~3% of reads mapped to the hg19 reference genome for both samples, and almost all of these reads also mapped to CanFam3.1, which indicated very low levels of human contamination in our data. Therefore, we conclude that both our samples appear to contain substantial authentic canine ancient DNA. The mean coverage for both samples was ~9x. Additionally, the mean coverage for the X and Y chromosomes was ~5x for both samples, indicating they are males.

Newgrange Dog

As well as the two ancient samples generated in this study, we also reanalyzed the ancient Irish Newgrange dog (henceforth known as NGD) described in Frantz *et al.* [22]. A BAM file containing only single ended unique mapped reads was provided by the authors of that study. In order to map to our modified version of CanFam3.1, reads were converted to fastq files using the `bamtofastq` function in `Bedtools`. All subsequent processing, beginning with mapping using `BWA aln,` were performed as described above for CTC and HXH. Mapping and post-mortem damage characteristics of NGD are also described in Supplementary Table 2.

Supplementary Note 5: Genotype Calling for ancient samples

We utilized distinct schemes to ascertain variants depending on the analysis being conducted (Supplementary Note 6). In all cases involving an ancient sample, we used a custom genotype caller implemented in Python (code available at https://github.com/kveeramah/aDNA_GenoCaller) that incorporates DNA damage patterns estimated from `MapDamage` using the likelihood model described in [15]. Briefly, damage patterns with respect to read position are fit with a Weibull distribution of the form *a* X exp(-(*x*^*c*) X *b*), where *x* is the proportion of damaged C>T or G>A bases at a particular position along the read (unlike [15]., we find a slightly better fit with a Weibull than when assuming exponential decay) (Supplementary Figure 31). We then calculated the likelihood of each possible diploid genotype using a model that incorporates the possibility of both sequencing error and post-mortem damage (see Table S13 in [15] for the likelihood expression for each possible allele, which can then be averaged for two alleles to obtain the likelihood for a particular genotype).

However, rather than simply reporting the best likelihood we also incorporated additional hard filtering steps to produce final genotype calls. Firstly, any site with less than 7x coverage was reported as missing. In addition, any position where the highest likelihood is a heterozygote must have a minimum Phred-scaled genotype quality of 30 or the next highest homozygote likelihood genotype was chosen instead. We found that this practice eliminated many false positives that are the likely result of post-mortem damage, resulting in much more balanced numbers of C>T vs T>C and G>A vs A>G heterozygous reference to alternate allele changes compared to when using the standard `GATK Unified Genotyper` caller (Supplementary Figure 4) (however, we note that when a site is already known to be segregating in other dogs or wolves, our algorithm and `GATK Unified Genotyper` are almost completely concordant for sites with >7x coverage). The balance was slightly improved for CTC compared to HXH, presumably as post-mortem damage is less extensive for the former. Despite its high mean coverage, balance was also noticeably improved for NGD. The transition/transversion ratio was only slightly less than other modern canid samples for CTC and HXH, while NGD, concomitant with its increased coverage, was clustered within the modern canid samples (Supplementary Figure 32 and Supplementary Figure 33) Base calls with a quality score less than 15 and reads with a mapping quality less than 15 were not included during genotype calling. Base calls with a quality score greater than 40 (which can occur during paired-end read merging) were adjusted to 40.

Supplementary Note 6: Contemporary SNP set construction and filtration

Sample and data collection

Variants from ninety-nine whole-genome canine sequences were analyzed in this study, including the CTC, HXH and NGD ancient samples. Illumina TruSeq libraries from a Great Dane and Iberian wolf were constructed from genomic DNA extracted from blood samples using the Qiagen PureGene Blood kit. The genomes of a Portuguese village dog (PT49), a Chinese Mongolian shepherd village dog (Dog05), and an Afghan hound (1756) were sequenced and processed using the methods described in Auton *et al.* [23]. All remaining samples were acquired from previously published datasets deposited on the NCBI sequence read archive (SRA) database (Supplementary Table 4; [23–27]). This includes a subset of read data from samples described in Decker *et al.* [28]. Variant call format files (VCF) for six samples from the Freedman *et al.* [25] study (basenji, dingo, golden jackal, Croatian wolf, Israeli wolf, and Chinese wolf) were acquired from John Novembre's group. Further processing for each canine is detailed below.

Variant calling and filtration

All dog genome sequence data was aligned against a modified version of CanFam3.1 reference genome with unplaced contig sequences combined into a single chromosome sequence (separated by 200 'N' characters) and including a representation of the non-pseudo autosomal Y chromosome sequence [29] using `BWA` [12]. PCR duplicates were removed by `Picard v1.62` (http://broadinstitute.github.io/picard), reads in regions with candidate indels were locally realigned and base quality scores were recalibrated using `GATK v3.4` [19], resulting in a dataset that exhibited mean autosomal coverages of 5.53-44.74x. We generated GVCF files (Genomic VCF) with a record for every position in the genome using `GATK HaplotypeCaller` (GATK v3.4) [19].

We generated three different call sets with different ascertainment schemes. The variant calling process for each call set is detailed below:

Call set 1-Comprehensive variants

This call set aims to include all variants from the 89 genomes (with bam files), 3 ancient genomes (with bam files) and Freedman's 6 genomes (with vcf files from Freedman). We first generated call sets from the three groups separately, took the union of the call sets, genotyped all the variants in each group, and then applied filtering accordingly.

1) We applied the `GATK HaplotypeCaller` to call variants (SNPs and indels) from the 89 genomes for which we had BAM files together. We applied a hard filter to remove sites that are within 5bp of an indel, and with MQ<25, QD<10, qual<33, mean DP < mean_read_depth/2, or mean DP> mean_read_depth x 2. The mean read depth for 89 genomes together is 879X. This set of variants contained 18.7 M SNPs. For the three ancient genomes, we applied the ancient DNA caller to discover variants in each ancient sample , using DP7, MQ15, BQ15, GQ30 as cut-off as described in Supplementary Methods 5. The variants from each ancient genome were merged, resulting in 5.8M

SNPs. For Freedman's genomes, we took variants from the vcf files from Freedman *et al.*

2) We took the union of these call sets and genotyped each variant in each group. For the 89 genomes, we genotyped those variants from 89 genomes together and applied a hard filter to remove sites with mean DP < mean_read_depth/2, mean DP> mean_read_depth x 2, or MQ<25. For the ancient genomes, we used the ancient DNA caller to genotype each variant, using DP7, MQ15, BQ15 as a cut-off. For Freedman's samples, we directly obtained genotype calls from Freedman's emit-all vcf files. The comprehensive SNP call set contains 24M SNPs. We additionally genotyped these SNPs in the Andean fox.
3) After removing sites with at least one missing genotype, we ended up with a final set of 7.4M SNPs.

Call set 2-Ascertained in ancient genomes

This call set only includes sites discovered in the three ancient genomes.

1) We applied the ancient DNA caller to discover variants in each ancient genome, using DP7, MQ15, BQ15, GQ30 as cut-off as described in Supplementary Note 5. The variants from each ancient genome were merged, resulting in 5.8M SNPs.
2) We applied the GATK UnifiedGenotyper to genotype these variants in other contemporary dog genomes and applied the aDNA caller to genotype those variants using DP7, MQ15, BQ15 as cut-off in each ancient genome. We directly obtained genotype calls from Freedman's emit-all VCF files. We additionally genotyped these SNPs in the Andean fox.
3) After merging VCF files and restricting to no missing genotypes, we ended up with a final set of 1.9M SNPs

Call set 3-Ascertained in New World wolves

This call set is designed to include only sites that are variables in New World wolves, a group that is sister to Old World wolves and dogs. Fan *et al.* [27] have demonstrated that the New World wolves have the least amount of admixture with dogs. D-statistics using golden jackal as the outgroup show no significant admixture between dogs and either of the New World wolves. We note that G-PhoCS analysis found 1.2%-3.2% gene flow from the basenji into the Mexican wolf population.

1) We chose three New World wolves (glw, ysa, mxa), each with ~20X coverage. We applied `HaplotypeCaller` implemented in `GATK` to call variants (SNPs and indels) from the three genomes together and applied a hard filter to remove sites that are within 5bp of indel, MQ<25, QD<10, qual<33, mean DP < mean_read_depth/2, or mean DP> mean_read_depth x 2. The mean read depth for the three genomes together is 76X. We additionally only keep variants with an alternative allele count of 1-5, resulting in 8.4M SNPs.
2) We applied `GATK UnifiedGenotyper` to genotype those variants in other contemporary dog genomes and applied the aDNA caller using DP7, MQ15, BQ15 as cut-offs to genotype those variants in each ancient genome. We directly obtained genotype calls from Freedman's emit-all vcf files and only retained variants that passed Freedman's Genome Filter (repeat divergence greater than or equal to 25, no CNV, MA, CpG). We additionally genotyped those SNPs in the Andean fox.
3) After merging VCF files and restricting to no missing genotypes, we ended up with 1.8M SNPs.

4) Ignoring the impact of recurrent mutation and post-divergence gene flow, this call set includes only mutations that either are private to New World wolves or occurred in the ancestral population of New World wolves, Old World wolves and dogs and thus have an essentially unbiased ascertainment with respect to Eurasian dog and wolf populations.

We performed Principal Component Analysis (PCA) on the three call sets to explore how the potential biases in each might affect the genetic differentiation observed in our data (Supplementary Methods 8). When we include all canids, we observe that in call sets 2 and 3 genetic differentiation across dogs is reflected in PC2, whereas all dogs cluster together in that PC in call set 1 (Supplementary Figures 36-38). However, when we only include dogs and remove groups used for ascertainment, the three call sets are consistent with the pattern observed in a PCA performed using the SNP array data, suggesting that the patterns of genetic differentiation amongst samples are robust across the different ascertainment schemes. Additionally, we explored the effect that potentially damaged bases (C > T and G > A transitions) could have in downstream analyses by performing a PCA on the SNP array dataset (Supplementary Figure 34) including our ancient samples with and without transitions. We found that the relative position of the three ancient samples was essentially unaffected, further validating our ancient DNA calling method for known SNP positions. Therefore, transitions were included in all subsequent SNP-based analyses. Although PCA reveals that the three call sets broadly capture the same variation patterns, we cannot control bias related to differences in coverage from call set 1 (which includes higher-coverage breed dogs) and cannot reliably identify false variation in call set 2 as a consequence of post-mortem damage. We therefore utilize call set 3, with variants ascertained in New World wolves, as the primary call set for most subsequent analyses. While gene flow between New World wolves and Old World canids could potentially bias the observed genetic variation in this call set, previous genomic studies have reported very low to negligible migration rates only between Mexican wolves and basenji/dingo [27], and another study suggests a potential old introgression from dogs to North American wolves (though we note that selection on standing variation cannot be ruled out) [30]. In both cases, these admixture events should have minimal impact on our analyses that primarily involve Eurasian dogs.

SNP array data collection and processing

Canine SNP array datasets were obtained from Dryad from [31] (doi:10.5061/dryad.v9t5h) and [32] (doi:10.5061/dryad.078nc). Sample information and the short-codes are summarized in Supplementary Table 3. We used `GATK GenotypeCaller` (GATK v3.4; [19]) to obtain genotype calls of SNP array loci from the whole genome vcf files (see methods above). For genomes from Freedman *et al.* [25] genotype calls were obtained directly from emit-all vcf files. We only included calls that passed sample filters (GQ greater than 20, DP less than 2*genome-wide depth, DL=0, and DV greater than 5). Ancient DNA genotyping was performed as described in Supplementary Note 5. After removing sites with more than 5% missing data across individuals our final SNP array dataset consisted of genotypes at 128,743 autosomal SNPs.

Supplementary Note 7: Mitochondrial Analysis

Mitochondrial haplogroup assignment

To infer the mitochondrial haplogroup placement of the HXH and CTC samples, we aligned their mitochondrial sequences to samples from the canid alignment released by [33] and NGD. The Thalmann *et al.* [33] canid alignment includes a comprehensive panel of modern dogs across four major clades (A-D), modern wolves, coyote, and both ancient wolf-like and dog-like mitochondrial sequences.

As expected, the sequencing depth of the mtDNA was high: the average sequencing depth was 179x and 208x in CTC and HXH samples, respectively. For the present analysis, we aligned the reconstructed HXH and CTC mitochondria sequences to the ancient canids (retaining samples with > 16kb callable sequence), modern dogs from the Thalmann *et al.*[33] alignment (90 samples) and NGD ancient dog, constructed a phylogenetic tree and identified which of the four major mitochondrial clades the two ancient German samples belong. For measurements of nucleotide diversity and phylogenetic reconstruction, we only used positions where genotypes were called across all samples. After removing missing sites, our mtDNA alignment consisted of 14,936 nucleotide positions and 616 variant sites. `MEGA 6.06` was then used to conduct a phylogenetic analysis [34]. A comprehensive pairwise comparison of nucleotide differences across the dataset showed that HXH and CTC mitochondrial sequences are more similar to each other (n = 5 differences) than to any other ancient canid or modern dog. Given the geographic proximity of the excavation sites of the HXH and CTC, a strong relationship between the mitochondrial haplotypes is not surprising. Furthermore, the low count of pairwise differences between the HXH and CTC suggests that random ancient artifacts are not a significant contribution to our call set of mitochondrial variants. HXH and CTC show higher sequence identities to NGD ancient dog than to any Thalmann sample: HXH has a slightly higher identity to NGD (n = 13 substitutions) than the CTC dog (n = 18 substitutions). Interestingly, the most similar haplogroup in the Thalmann *et al.* [33] dataset was the ancient dog-like sample from Germany (Germany 12.5 kya), which differed from the HXH and CTC dogs by 17 and 22 sites, respectively. A NJ tree built with a TN93 substitution model (500 bootstraps) of our alignment revealed that CTC and HXH mtDNA haplotypes are members of the C clade of modern dogs (100% support) (Supplementary Figure 5).

Once we characterized the HXH and CTC dogs as members of clade C, we sought to identify more granular haplogroup information as clade C branches into the C1 and C2 haplogroups. We downloaded 24 C1 and C2 mitochondria samples from GenBank with haplogroups annotated by Duleba *et al.*[35] and included them in our alignment (EU789659, EU789760, EU789661, KM061561, EU408267, KM061540, KM061534, DQ480489, KM061534, KM061535, EU789764, EU789762, KM061533, KM061497, KM061488, KM061481, KM061475, KM061591, KJ637139, KJ637136, EU789753, EU789751, EU789750, and EU789657) (Supplementary Figure 8).

We performed an additional analysis including the low coverage (8,667 retained nucleotides) Bonn Oberkassel ancient sample (Germany 14,700) from the Thalmann dataset. Relative to the alignments used to generate Supplementary Figure 5 and Supplementary Figure 8, this alignment

is significantly diminished in nucleotide positions (n=8,090) and variant sites (n=369). Our NJ tree of this alignment shows the Blue Heeler as an outgroup of the C clade and an unexpectedly long branch length of the Bonn Oberkassel dog, which may be the result of a reduction in mitochondrial diversity in this dataset and DNA damage of the Bonn Oberkassel dog, respectively (Supplementary Figure 6). We performed a transversion-only analysis of this alignment (Supplementary Figure 7). However, by reducing diversity of the dataset we no longer find bootstrap support for our clades of interest. Given the unexpected topological changes and additional diversity attributed to DNA damage of the Bonn Oberkassel dog, we interpret the high coverage only alignment as a more accurate estimate the true mitochondrial tree.

Supplementary Note 8: Neighbor-joining tree estimation

We computed a matrix of pairwise genetic distances between each canid in our autosomal whole genome SNP set ascertained in the New World wolves (Call set 3) and built a NJ tree using the Andean fox as an outgroup. The genetic distance d(X,Y) between diploid genomes is calculated using the formula in [36]. A hundred bootstrap replicates were generated by dividing the genome into 5 cM windows and sampling with replacement from those windows. We used the `R` package `ape` to generate the NJ tree and used `nw_support` from the `newick_utils package` [37] to compute bootstrap support.

We found that all coyotes were clustered into a single clade, sister to all gray wolves and dogs (Supplementary Figure 9). All New World wolves (Mexican, Great Lakes, and Yellowstone wolves) first branched out, separated from the rest. Since we are using SNPs ascertained in the New World wolves (Call set 3), we are missing sites that are non-variable in the New World wolves, which likely explains the reason why the New World wolves do not form a single clade. Dogs were sister to the Old World wolves, consistent with previous findings [27]. Within the dog phylogeny, the first branch with 100% bootstrap support contained village dogs from South China, Vietnam, Borneo and breeds that originated from those areas (chow chow and shar-pei from China, dingo originated from East Asia before later being brought to Australia), consistent with a recent finding that dogs from southeast Asia are one of the basal group [38]. Sister to the group with mostly southeast Asian dogs, are the rest of the village and breed dogs that have a tree structure only with high inner branch support: Sub-Saharan village dogs with the basenji, Indian village dogs, European village dogs with most modern breed dogs, and the three ancient samples (CTC, HXH and NGD) each form distinct clades.

We further removed most breed dogs, North China village dogs (found to be admixed with European dogs, see Supplemental Note 10) and Papua New Guinea village dogs from further analysis. The remaining village dogs and ancient breeds were then used to generate a second NJ tree (Supplementary Figure 10). We found much higher bootstrap support with respect to village dogs with this tree. Sister to the group with mostly southeast Asian dogs, there are three major clades, a clade containing Indian village dogs branched out first, then two sister clades, one containing Sub-Saharan, Egypt and Qatar village dogs and the other containing ancient dogs, European breeds, Portuguese and Lebanon village dogs. Among the third clade, CTC branches out first, followed by NGD and HXH, which forms a clade and sister to other European breeds and Portuguese, Lebanon village dogs.

Supplementary Note 9: Population structure analyses

Principal component analysis

SNP array-based PCA

Principal component analysis (PCA) for both the SNP array and genome sequenced SNP datasets were run using `smartpca`, part of the `EIGENSOFT` package version 3.0 [39]. Previously published samples consisting of 783 globally distributed village/free-breeding dogs, 196 dogs from 164 different breeds and NGD [31,32] were examined in the SNP array-based PCA in addition to our two German ancient samples (CTC and HXH). Diploid and pseudo-haploid PCAs were generated using this data set where: i) the PC space was defined by the village/free-breeding dogs and the breed dogs and ancient samples were projected onto the PC space, and ii) the PC space was defined by all samples. The pseudo-haploid ped file was generated with a custom script that randomly chooses one allele at each site. In addition, we generated a pseudo-haploid PCA where sites potentially subject to post-mortem damage (C <> T, G <> A) were removed, resulting in 22,699 filtered SNPs.

We observed little difference between the diploid and pseudo-haploid PCAs, with and without projection, and removing potentially damaged sites (Supplementary Figure 34-35). Subsequently, we generated a diploid PCA defining the PC space on village dogs and did not remove the potentially damaged bases as it appears that our ancient genotype calling largely accounts for post-mortem DNA damage as predicted. To ensure covariance amongst dogs of European ancestry did not dominate the first few components of the PCA, European breed dogs and village dogs of the Americas (both of which possess large amounts of European ancestry) were removed from the PCA.

Genome sequence-based PCA

PCA was performed on the genome sequence SNP data for all three call sets (see Supplementary Note 6.2). Since the SNP array PCAs showed little difference between diploid, pseudo-haploid, and removing potential damaged bases, we generated only diploid PCAs for the genome sequence sets, defining the PC space on all samples. For call sets 1 and 2, we generated PCAs with all samples (n = 99); these sets of PCAs include 3 coyotes, 1 golden jackal, 1 fox, 14 geographically distributed gray wolves, 45 geographically distributed village dogs, 32 dogs from 28 distinct breeds, and the three ancient samples (CTC, HXH and NGD). We applied "--bp-space 5000" filter to thin SNPs; this resulted in 376,777 sites for Call set 1 and 306,319 sites for Call set 2. For Call set 3, we generated a PCA with the same set of individuals but excluded the New World wolves (n = 94); these wolves were excluded because a subset of the New World wolves were used to ascertain the SNPs in Call set 3. We applied a minor allele frequency filter ("--maf 0.05") in order to retain SNPs segregating in the current set and a "--bp-space 5000" filter, which resulted in 261,081 sites. For all three call sets, we additionally performed analyses that excluded the coyotes, jackal, fox, gray wolves, and outlier dogs (basenji, dingo and a Sub-Saharan village dog, 1756); we applied "--bp-space 5000" and "--maf 0.05" filters to each set which resulted in 300,864 sites for Call set 1, 290,917 sites for Call set 2, and 245,363 sites for Call set 3. All site filtering for PCA was done using PLINK v1.90 [40].

In the PCA of Call set 1 with all individuals, PC 1 distinguishes coyotes, jackal, and fox from the

other samples, while PC 2 separates the wolves from the dogs. Interestingly, little variation is seen in the dog grouping which forms a tight cluster and includes CTC, HXH, and NGD (Supplementary Figure 36). However for Call set 2 and 3, PC 1 separates the wolves, coyotes, jackal, and fox from dogs and PC 2 explains variation among the dog samples (Supplementary Figure 37, 38). Additional PCs reveal variation among the outgroup and wolf samples. Overall, it is evident that CTC and HXH are dogs, and are not similar to modern wild canids.

When we remove the outliers and outgroups used for ascertainment, PC 1 and PC 2 are similar to the SNP array PCA, with modern breed dogs generally clustering together, village dogs distributing following geographic patterns, and “ancient”/”basal” breed dogs (ie. Siberian Husky, Saluki, etc.), situating away from the modern breed dogs and grouping near the village dogs matching their geographical origins (Supplementary Figure 39). Also, like the SNP array analysis, CTC falls along the Indian axis and HXH and NGD fall nearest the European core but along the Southeast Asian axis. This general pattern is seen across all SNP call sets (Supplementary Figure 40-41).

SpaceMix

Spacemix is a relatively new method [41] that models allele frequency covariance amongst populations to create a “geogenetic map” . The method provides a similar description of isolation-by-distance-based population structure as PCA but also has the added advantage of visualizing major deviations of increased covariance that are likely to reflect long-distance admixture events. As this analysis will have most power with large numbers of populations, we performed this analysis on the three ancient genomes, Old World village dog populations and Old World wolves from the SNP array data set. In addition, this method requires independent SNPs and relatively even sample sizes from each population. Therefore, the data was filtered by choosing SNPs randomly such that the minimum distance between any pair was 100Kb, and the number of individuals in each population was limited to five (for populations with more, five were randomly chosen). This resulted in a dataset containing 19,021 SNPs in 51 populations.

SpaceMix requires fitting a complex parameter space and thus utilizes an Markov Chain Monte Carlo (MCMC) search to find the most likely model parameters and also estimate confidence intervals. We ran 10 initial burn-ins of 100,000 generations to identify the best starting position for the MCMC chain, followed by a single long run for 10,000,000 generations, which appeared to be sufficient for the chain to stabilize, sampling every 1,000 generations. We performed eight separate iterations of this process and present results from the run with the highest peak probability. The model appeared to largely fit the major features of the data for this run (Supplementary Figure 42). Latitude and Longitude coordinates for each population’s country of origin was also provided to inform priors for the fitting of geogenetic space.

A plot of populations in geogenetic space separates wolves and dogs as expected (Supplementary Figure 21). In addition the clustering of dogs essentially replicates the results from the PCA for individual samples, with HXH found close to NGD and CTC located closest to Afghanistan village dogs. Though the authors of SpaceMix note that it has a tendency to underestimate admixture proportions, the software inferred that 25 populations had admixture from an outside source greater than 5%. Visualizations of all inferred admixture events inferred to be greater than 5% are given in Supplementary Figure 44 but will not be discussed at length here as many are

replicated from the NGSadmix and *f3* analysis. For example, the highest admixture component was observed for free roaming chinese dogs (pCH), which also had the most negative *f3* statistic. The figure for this population shows pCH drawing ancestry from a population extending through Europe (Supplementary Figure 44r) (SpaceMix appears to overshoot potential source populations on occasion).

Perhaps most strikingly, while HXH and NGD showed no particular evidence of strong admixture, `SpaceMix` inferred a strong admixture source component (~10%) for CTC from the Old World wolf geogenetic space (Supplementary Figure 22). Given CTC is represented by only a single sample and thus allele frequencies for its underlying population can only be crudely estimated, this suggests a fairly strong wolf component. A similar but less strong inference is made for village dogs from Egypt (Egy, 4%), Iran (pIR, 4%) and Saudi Arabian (pSA, 6%). Interestingly SpaceMix infers that the Israeli wolf draws approximately 10% of its ancestry from this same dog geogenetic space (i.e. in the opposite direction) (Supplementary Figure 43). Amongst the other wolves the Iranian and Indian wolves do not appear to draw substantial ancestry from dogs, while the European (~7%) and Chinese (1%) wolves are inferred to possess dog ancestry compatible with their geographic location (though Asian dogs were found closer to the general wolf cluster in geogenetic space, which may reduce the inferred admixture proportion for Chinese wolves).

SNP array-based `ADMIXTURE`

`ADMIXTURE` (v.1.22) [42] was run on a subset of individuals (n = 108) from the SNP array data set; this included our two ancient German samples, NGD and a global representation of village dogs. We generated three replicates, with different seeds, and plotted the run with the overall lowest cross validation error across all K (number of defined clusters) values. The cross validation procedure was also used to determine that three ancestral populations ($K = 3$) was the best value for K (Supplementary Figure 45).

At $K = 2$, Southeast Asian village dogs separate from the rest of the village dogs; this ancestry is seen in most of the other village dogs and westwardly decreases in percentage among the samples. CTC, HXH, and NGD have a moderate amount of Southeast Asian ancestry, 23%, 17%, and 12% respectively. At $K = 3$, in addition to the Southeast Asian grouping (red), Indian (blue) and European (green) ancestries emerge (Supplementary Figure 15). And at $K = 4$, African ancestry appears. Interestingly, CTC harbors Indian and African ancestry (27% and 8%), while HXH and NGD do not. Finally, at $K = 5$, Europe additionally divides into two groups. Overall, the results of this analysis are consistent with results of the other population structure analyses; both CTC, HXH and NGD contain a moderate amount of Southeast Asian ancestry, while only CTC contains Indian and perhaps a small amount of African ancestry.

NGSadmix

As many of our genomes are medium to low coverage, including our two ancient samples, we used `NGSadmix` [43] to infer population ancestry for each genome given pre-specified numbers (K) of clusters. While similar in spirit to `Admixture` [42], `NGSadmix` accounts for genotype uncertainty by integrating over all three possible genotypes for a site that is known to be biallelic. We extracted genotype likelihoods for our genome data for all village dogs, Old World

wolves and the three ancient samples at SNPs that were separated by at least 10,000bp (179,072 SNPs) and ran `NGSadmix` under default parameters (changing these had little effect) for *K*=2-10 (for the purposes of brevity only results for *K*=2-6 are presented here). We performed multiple runs for each *K* and found them to be highly consistent.

Unsurprisingly, for *K*=2 wolves separate from dogs, though some dogs, including CTC along with African, Middle Eastern and Indian village dogs, demonstrate small amounts of putative wolf ancestry (some wolves also demonstrate dog ancestry) (Supplementary Figure 16). At *K*=3 dogs separate into two primary clusters, one containing southeast Asian dogs from Vietnam, Borneo and some southern Chinese dogs. Other dogs are either assigned exclusively to the other cluster or are mixed. As in the `ADMIXTURE` analysis of the SNP array data CTC, HXH and NGD all demonstrate a small east Asian component unlike modern European dogs. This is compatible with HXH and NGD's position in the PCA. The Chinese wolf appears to possess substantial Southeast Asian dog ancestry, consistent with local dog/wolf gene flow. For *K*=4, the non-southeast Asian cluster is further subdivided into two clusters, with Europeans and Lebanese orientating one cluster, and Indian and African dogs orientating the other. Noticeably, CTC, unlike HXH and NGD, draws a significant amount of ancestry from the Indian/African cluster, which again would be consistent with this sample's PCA position intermediate of European, Indian and East Asian village dogs and the `ADMIXTURE` analysis. Subsequent *K* values begin distinguishing specific village dog populations.

Supplementary Note 10: *f*-statistics and *D*-statistics analysis

Patterson *et al.* [44] have developed a general approach for examining allele frequency correlations amongst populations to make powerful inferences about population histories that involve admixture. This approach involves estimating *f*-statistics and D-statistics within the context of certain theoretical expectations given a particular demographic scenario. The major advantage of these methods is that inferences can be made with even low coverage single genomes (<1x) and that inferences are relatively robust to ascertainment biases. Therefore we implemented these methods to better understand the history of our ancient genomes. Genetic map positions for each SNP used in these analyses were inferred from Auton *et al.* [23].

Outgroup *f3* analysis

We estimated the relative genetic similarity of the three ancient dogs to each other and various modern dog and wolf populations by calculating an outgroup *f3*-statistic [44] of the form F3 (C : A, B). We set either CTC, HXH or NGD as population B and applied this test to both the SNP array data (with the golden jackal set as an outgroup and population A set as a Old World village dog or Wolf population, with individuals from the same population merged) and the genome data (Call set 3 with Andean fox set as an outgroup and population A set as individual Village dog samples, plus the basenji and dingo). *f3* statistics were estimated using the `qp3pop` program (modified to allow more than 22 autosomes) found within the `Admixtools` software package. Standard errors were estimated using a weighted block jackknife as previously described in Patterson *et al.* [44].

Results using both datasets are highly consistent. When set as the target B both HXH and NGD show greatest similarity to each other followed by modern European village dogs, and are most distant to East Asian and Indian village dogs. CTC shows a similar level of similarity to HXH and NGD as other modern Central Asian and Middle Eastern village dogs (Figure 2, Supplementary Figure 13A,C, Supplementary Figure 14A,C). However, when CTC is set as population B, it shares the most similarity with HXH, then NGD and then European dogs (Figure 2, Supplementary Figure 13B, Supplementary Figure 14B). This may suggest some continuity leading from HXH to CTC for their European component (tested formally below).

MixMapper

`MixMapper` [45] provides a method for inferring admixture events within the context of a population tree based on *f*-statistics. The approach is similar in principle to `TreeMix` in that a bifurcating tree is first fit to allele frequency correlations and migration is then inferred on top of the tree if it improves the fit of the data. However the `MixMapper` approach is less automated than `TreeMix` and is considered a bottom-up approach, allowing more control and reliability in testing specific hypotheses with regard to admixture events between two or three populations.

We applied the `MixMapper` approach to both the SNP array data and genome SNP data (Call set 3). First, we used the *f3* statistic to determine which populations were likely to be admixed. Again, this test statistic is of the form F3 (C : A, B). However, when C is not chosen specifically to be an outgroup, any phylogeny where population C is admixed between two populations

descended from A and B will have some portion where allele frequencies are negatively correlated, which can result in negative *f3* values. Z-scores for significant negative *f3* statistics were again calculated using a weighted block jackknife. All combinations of three way phylogenies were examined for the three ancient dogs, Old World modern village and free-breeding dogs, dingo, basenji, wolves (Old and New World), coyotes, golden jackal and Andean fox, with individuals grouped by population. Using SNP array data 30 non-New World canid populations had significantly negative (Z-score <-2) *f3* statistics (Supplementary Table 5), while for the whole genome data 12 populations were putatively admixed (Supplementary Table 6). These populations did not include CTC, HXH or NGD, but the *f3* test has low power when the target consists of only a single diploid individual.

f2 statistics were then used to construct scaffold NJ trees for the remaining non-admixed populations (for genome data we also excluded the three ancient genomes and modern dog populations represented by only a single genome, basenji, dingo, Mongolian shepherd and the China-Kazakhstan dog). These trees consisted of 25 (Supplementary Figure 17) and 10 (Supplementary Figure 18) populations for the SNP array and genome data respectively (CTC, HXH and NGD were not included in these trees). We note that the topology of these trees are consistent with those constructed in Supplementary Note 8.

Two-way admixture fitting was then performed for CTC, HXH, and NGD separately. This involves testing every pair of branches in the scaffold as potential source populations for a third target population by solving a system of *f2*-based equations, and choosing the pair with the smallest residual norm value. Significance was assessed via bootstrapping by dividing the genome into 50 even sized blocks of SNPs, resampling with replacement 500 times and recalculating the test statistics.

For both SNP and genome data `MixMapper` identified a potential admixture event for both HXH and NGD involving ~80% ancestry from one source population leading to modern Europeans and another source leading to a modern East Asian populations, consistent with the `ADMIXTURE`/`NGSadmix` analysis (Supplementary Table 7-8). Some dog-wolf admixture was observed for both samples using the SNP data, but was not replicated with the genome data. However multiple potential admixture events were identified for CTC involving branches leading to modern European, Indian, East Asian, Middle Eastern and wolf populations (Supplementary Table 9).

Given the *f3*-outgroup test showed that HXH demonstrated the greatest similarity to CTC when the latter was the target, we also attempted to fit a three-way admixture event with HXH being an initial admixed population (between the European and East Asian populations shown in Supplementary Table 7, and CTC being a descendent of HXH as well as being admixed with an additional third population. For both SNP array and genome data MixMapper found the greatest support for a three-way admixture event for CTC involving HXH and a population leading to modern Indian or Saudi Arabian village dogs as well as Israeli wolves, similar to the pattern identified in the `ADMIXTURE` and `NGSadmix` analysis (Supplementary Table 11).

Thus our results suggest that CTC represents an admixed lineage that is directly descended from HXH, which itself is admixed and may represent an ancient branching from the modern

European lineage.

PCA of the SNP array data demonstrates that CTC occupies a similar space to Afghanistan (Afg) village dogs for the first two PCs. Supplementary Table 5 shows that Afg village dogs appear to be the result of an admixture event, and while many pairs of test source populations can generate negative *f3* values, the most significant was when utilizing pPO and I-W (i.e. a European and an Indian population). Negative *f3* values are also generated when utilizing pPO (and other European populations) and Viet. Thus Afg shows many of the same admixture signals as CTC. We hypothesized that admixture events seen in Afg village dogs may have the same origins as those in CTC, which itself is potentially descended from HXH. Therefore we included HXH as part of the scaffold NJ tree and allowed `MixMapper` to model a two-way admixture event for both CTC and Afg village dogs. Every putative pair of sources inferred for CTC involve HXH and no modern European village dogs (Supplementary Table 13). However, the fitted pairs for Afg not only include HXH but also Bosnian (Bos) and Portuguese (Por) village dogs. Thus it is seems likely that there has been additional European admixture in Afghanistan village dogs that are distinct from that seen in the CTC dog.

D-statistics to examine wolf admixture

Fan *et al.* [27] has recently demonstrated that many wolves from across the world contain substantial ancestry derived from dogs, including some of the dogs used in this study (for example >20% dog ancestry in the Israeli and Iberian wolf). We calculated D-statistics of the form *D*(A,B,Wolf,Outgroup) using the `qpDstat` function in `Admixtools` to examine potential differences in dog-wolf admixture between the three ancient dogs. Because of the potential effects of ascertainment bias that might result from choosing sites that are variable in dogs as part of the construction of SNP arrays, we limited this analysis to the whole genome data only (Call set 3). In addition we limited our analysis to modern village dogs used in the scaffold tree for the `MixMapper` analysis. All Old World wolves were examined individually. Z-scores for significant negative D-statistics were again calculated using a weighted block jackknife, and we only considered D-statistics with a Z-score of |3| when using Andean fox as an outgroup.

The only significant difference when examining *D*-statistics of the form *D*(ancient_sample, ancient_sample, Wolf,Outgroup) was for HXH and NGD sharing more alleles with the Spanish wolf than CTC (Supplementary Table 12). Given that the Spanish wolf likely has substantial dog-wolf admixture (See section below), this result is probably because of the decreased European component in CTC because of its apparent Indian-like admixture. Though not significant, the only genomes CTC was consistently closest to were the Indian and Iranian wolves, suggesting this may be the source of wolf admixture observed in this sample.

f4 ratio test

We applied the *f4*-ratio test (qpF4ratio in AdmixTools package) to estimate ancestry proportions in an admixed population for our ancient and modern dogs. Consider a topology where X is admixed from two source populations that are ancestral to B and C population, with proportions α and 1-α, respectively. The admixture proportion α is calculated as the ratio between *f4*(A,O; X,C) and *f4*(A,O; B, C). Population A is more closely related to population B, and population O is an outgroup. We used the Andean fox as the outgroup, Indian dog as A, European dog as B,

Southeast Asian village dog as C. The estimated proportion α represents European dog ancestry, and 1-α represents the southeast Asian dog ancestry. We found around 19.1%-27.8% Southeast Asian village dog ancestry in HXH and 20.9%-30.3% Southeast Asian village dog ancestry in NGD. (Supplementary Table 10). We also attempted different orientations of the tree to estimate Indian dog ancestry in CTC, but due to the complexity of the admixture, the appropriate topology to utilize was unclear. In order to estimate potential dog ancestry in wolves, for each wolf as X we used village dogs from South China as A, the Indian wolf as C (based on Fan *et al.* [27] this is the wolf amongst our dataset with the least dog ancestry) and alternated each dog as B. We then summarized the range of dog ancestry in each wolf (Supplementary Figure 46). The Spanish wolf has the most dog ancestry, 16% to 24%, followed by the Israeli wolf, 12% to 18%, then the Chinese wolf, 12% to 17%. Consistent with the hypothesis that the Indian wolf has the least dog ancestry, setting the Indian wolf as X produced negative α values.

Supplementary Note 11: ADMIXTUREGRAPH analysis

ADMIXTUREGRAPH (implemented in qpgraph in the Admixtools package) performs an approximate likelihood maximization to best fit *f2*, *f3*, *f4* statistics for all combinations of populations considered in a user-defined model of population demography that includes population split and admixture [44]. MixMapper can be considered an automated version of ADMIXTUREGRAPH. However ADMIXTUREGRAPH can incorporate more complex models with multiple admixture events and ghost populations, providing the opportunity to fit all f-statistics (MixMapper only minimizes these within the context of one or two admixture events on a scaffold tree). Therefore we drew upon the various demographic inferences from the previous analyses to use ADMIXTUREGRAPH to find a model of population demography for our ancient and modern canids. We restrict our analyses to the genome data (Call set 3) and utilize the Andean fox. We consider a *f-statistic* to be fit if the inferred value in the model is within three standard errors of the estimated value from the data (| *Z*-score | >3), as utilized previously by others [46]. Note that occasionally best fit branch lengths between two nodes will have a value of 0. While these could be collapsed into descendent nodes, we generally keep them in the graph to clearly demonstrated the topology of the model (unless the admixture graph becomes to visually unwieldy), as they have no effect on the overall statistical fit of the model.

Fitting HXH and NGD

We first attempted to fit a model for HXH and NGD (individually at first) and modern village dogs as they show a similar pattern of population structure and admixture across various analyses involving primarily European ancestry with an additional Southeast Asian component. Implementing a model with HXH/NGD being sister to European village dogs and Borneo village dogs (representing Southeast Asian village dogs) results in one *f4* outlier. However, allowing the ancient samples to be admixed between European and Bornean village dogs results in no outliers (i.e. the model fits the data) for both HXH and NGD, with the highest *Z*-score being 0.071, though HXH demonstrated a slightly higher Southeast Asian-derived component (Supplementary Figure 19).

We next included European (represented by the Italian wolf, as this was the least admixed based on the *f4*-ratio analysis) and Chinese wolves in the admixture graph model above. When including wolves as sister to dogs we obtained 10 outliers. However, when we allowed regional-specific admixture from dogs to wolves (as indicated in the NGSadmix analysis) we obtained a model with only one barely significant *f4* outlier (Z=3.074) for HXH, no outliers for NGD, and similar admixture proportions for both the ancient samples as before (Supplementary Figure 47).

When attempting to include HXH and NGD on the same graph, placing them as sister populations descended from a single admixed population resulted in five outliers. One (again barely significant, *Z*=3.08) *f4* outlier can be obtained when allowing HXH and NGD to have distinct admixture events. This is likely not a realistic model but instead reflects the limitation that ADMXITUREGRAPH cannot model differential admixture in a structure population (Supplementary Figure 48).

Fitting CTC

In addition to the Europe and Southeast Asian components observed in HXH and NGD, CTC appears to possess a complex history, with an ancestry component found predominantly in modern Indian village dogs, as well as potentially some Indian admixture. It also appears to be genetically closest to HXH, suggesting some level of continuity when considering both samples are from Germany. As such, it proved extremely difficult to fit a suitable admixture graph for CTC along with other dogs and wolves. Ignoring HXH initially, one model that generates no outliers can be obtained by a) adding Indian village dogs to the graph, b) incorporating both Southeast Asian dog and wolf admixture into Indian village dogs and c) CTC being the result of European and Indian dog admixture (Supplementary Figure 23). While caution must be applied because of the possibility of overfitting such a complex model, we do note that all such events were supported by other analyses.

Incorporating HXH into this model as a descendent of CTC followed by Indian admixture results in two *f4* outliers (one barely significant, Z=3.013) (Supplementary Figure 20). While again cautioning against overfitting (and we note a very high Indian component in CTC of 71%), this model fits the data far better than when CTC and HXH are allowed to descend from distinct European lineages, which result in 74 outliers, primarily because `ADMIXTUREGRAPH` cannot fit the *f*-statistics involving HXH and CTC as they are too phylogenetically distant on the admixture graph. This supports previous analyses suggesting some level of population continuity for the European component of HXH and CTC, and that the subsequent Indian-like admixture must have occurred into a descendent of HXH.

Finally, we included NGD into the above model (Figure 5a). This resulted in four *f4* outliers (but still no *f2* or *f3* outliers) (Supplementary Table 14). While we could potentially add additional admixture events or ghost populations to eliminate these outliers, given the complexity of the admixture graph already, we feel it would be unreasonable to do so, especially since we do not consider the effects of multiple testing when utilizing the Z scores.

Supplementary Note 12: G-PhoCS analysis to determine Neolithic-Modern European divergence time

G-PhoCS settings

`G-PhoCS` [36] is a full-likelihood-based method that uses independent loci in order to perform Bayesian coalescent-based inference of divergence times (τ), population diversity (θ) and, if specified, migration bands. To apply this method to our canid data and estimate when our ancient samples (and in particular our oldest sample, HXH) may have diverged from modern European samples, we performed a LiftOver on the 16,434 "neutral loci" (interspersed genomic segments of 1kb length) previously identified by Freedman *et al.* [25]. We then generated alignments at these loci that included the golden jackal, multiple Old World wolves (Israeli, Croatian, Chinese and Indian), several village (each from South China, India and Portugal) and breed dogs (boxer and dingo), and the two ancient samples. Genotypes for all samples were called from recalibrated BAM files using reads with mapping quality >=15 and base quality >= 15 for modern samples, and calls with genotype qualities less than 30 and less than 7x coverage were marked as 'N'. Ancient samples were called as described above (see Supplementary Note 5). We masked sites for all samples if any sample appeared to have a 'CpG' dinucleotide sequence and removed loci if any sample had complete missing data. As we tested many different combinations of samples and as G-PhoCS is computationally intensive, we often randomly selected 5,000 loci to perform the analysis. The final results presented in Figure 5 however are based on the full data set (16,434 loci), as well as select analyses where indicated. When the two ancient samples were included, we set the age of HXH to be 7,000 years old and the age of CTC to be 5,000 years old assuming the mutation rate μ=1 x 10^{-8} bp/generation and generation time = 3 years. For each run we ran 500,000 MCMC iterations and used Tracer [47] to examine the chain convergence.

G-PhoCS results

We built a NJ tree based on the neutral loci alignments on selected samples using the same method as described in Supplementary Note 9. This tree and the global NJ tree were largely concordant (Supplementary Figure 49). We ran G-PhoCS on the following tree structure, (((((Boxer, Village_Europe), Village_India), Village_ChinaS), ((wolfY, IsraeliWolf), ChineseWolf)),GoldenJackal) (Figure 5b), with wolfY either being the Croatian or Indian wolf. We then ran `G-PhoCS` with each ancient sample added separately, with HXH or CTC sister to the ancestral of boxer and Village_Europe. For the migration bands, we set Village_ChinaS dog with the Chinese wolf, and other modern village dogs with the Israeli wolf, Village_India dog with the Indian wolf, the Israeli wolf with the golden jackal, and dog/wolf ancestors with golden jackal. Evidence for these migration bands either come from significant *D* statistics calculated by `Admixtools` (Supplementary Table 24) or were previously identified by Freedman *et al.*[25]. For ancient samples, we added migration between South China village dogs and HXH, Indian village dogs and CTC, South China village dogs and NGD, and each wolf with the ancient samples. We found that 500,000 iterations were sufficient to establish convergence for all parameters and we sampled the the last 200,000 iterations to estimate the posterior distribution and calculate the 95% CI for each parameter.

When assuming the slower mutation rate, μ, of 4 x 10^{-9} bp/generation (also see next section),

examining only modern village dogs resulted in an estimate of the Asian and non-Asian dog divergence time of 17,500 to 23,900 years ago and dog-wolf divergence time approximately 36,900 to 41,500 years ago. As seen previously in [25,27], we observed that wolves appeared to diverge rapidly (within the space of ~1,000 years). The branching of the main dog lineages occurred over a much longer period of time: after the initial Asian-non-Asian dog divergence, the divergence between Indian and European dogs occurred around 13,700 to 17,900 years ago, while the divergence between European village dogs and modern breeds were around 4,300 to 9,300 years ago. (Supplementary Figure 50, Supplementary Table 15). We found that using the Indian or Croatian wolves generally gave similar results to each other. We compared our divergence time estimates with the ones in previous studies using `G-PhoCS` (Supplementary Table 16). We found that dog-wolf divergence time is similar to Freedman's estimates when using the same mutation rate; however our dog divergence time is younger than the Freedman *et al.* [25] estimate (33,000 years) but similar to the Wang *et al.* [38] estimate (24,000 years) [38] . This discrepancy appears to be result of the sample used to represent the southeast Asian lineage. Freedman *et al.* used the dingo [25], while our study and Wang *et al.* [38] used village dogs from South China. When we changed the village dog from South China to dingo, we observed higher dog divergence time estimates, while other estimates remain the same (Supplementary Table 15). We primarily emphasize result using the village dogs versus the dingo [25] because the latter are generally considered to be only semi-domesticated.

When adding either of the ancient dogs, we found that the divergence time between European dogs and CTC was ~18,000 years and HXH more than 30,000 years, much older than the estimated European-Indian dog split inferred from using only modern samples. These in turn led to a larger European-Indian dog split (similar to the divergence time between European and ancient dog) and dog wolf divergence time almost double the original (60,000-80,000 years). Though our genotype calling did substantially lower the number of false positive due to post-mortem damage, when examining the number of private variants for our two ancient samples at these loci, we found a slight excess compared to the modern European village dogs. Thus we anticipate that false positive singleton variants due to the post-mortem damage and lower coverage of the two ancient samples may be artificially elongating branch lengths in the `G-PhoCS` analysis. Therefore we devised a new method for estimating the HXH-European split time using the `G-PhoCS` results for only the modern samples as a baseline that would be robust to this signal (see below Supplementary Note 13). We also tried adding NGD to the G-PhoCS analysis, which was sequenced to 28X. The divergence time for European and NGD was ~20,000 years ago, and the European-Indian dog ~ 23,000 years ago, much older than the estimated European-Indian dog split inferred from using only modern samples. Although NGD has higher coverage and better genotype calls, false positive singleton variants due to the post-mortem damage are likely still affecting `G-PhoCS` results (Supplementary Figure 51). As seen in Supplementary Note 5, even with this high coverage using standard genotype callers will still substantially overestimate C>T and G>A mutations, while our aDNA genotype caller may still not fully capture all damage despite clearly improving the overall false positive rate.

We found that the effective population size of village dogs are 5 to 10 folds higher than that of boxer. The effective population size of Israeli wolf is the highest among all wolves and golden jackal (Supplementary Figure 52). We also inferred the total migration rate in our analysis, calculated by multiplying migration rate with the time that both population exists during the

migration period. Total migration rate can be viewed as the probability that a lineage in the target population will migrate into the source population (Supplementary Figure 53). We found that there was significant non-zero migration from Israeli wolf to boxer, European village dog and Indian village dog. We also found that the total migration rate from the Israeli wolf to Indian village dogs was around 0.47 when utilizing Croatian wolf as WolfY, much higher than estimated migration to other dogs. However, when utilizing the Indian wolf as WolfY, the total migration rate from the Israeli wolf to Indian village dogs was reduced to 0.02, while total migration rate from Indian wolves to Indian village dogs was 0.34, suggesting this was the more likely source of wolf admixture in Indian village dogs. We hypothesize that this signal is of similar origin to the high migration rate (0.12-0.24) observed in Freedman *et al.* [25] between the Israeli wolf to basenji. We also found significant non-zero migration between the Chinese wolf and village dogs from South China, the Israeli wolf to the golden jackal and a dog/wolf ancestors to the golden jackal, all of which again are concordant with the results from Freedman *et al.* [25].

We also performed a G-PhoCS analysis using the same phylogeny but without any migration band setting. We found that the divergence time among wolves and the divergence time of dog/wolf ancestral population were smaller when migrations between wolves and dogs are neglected. However, the divergence time amongst dogs were not affected with/without migration (Supplementary Figure 54).

In order to confirm that the divergence time between Asian and non-Asian dogs is not affected by the choice of outgroup and possible migration between golden jackal and Old World wolves, and between dogs and wolves, we performed another G-PhoCS analysis using the following tree structures, ((((Village_Europe1,Village_Europe2), (Village_ChinaS1, Village_ChinaS2)), (yellowstoneWolf1, yellowstoneWolf2)), outgroup), with outgroup being either golden jackal or Andean fox and no migration settings. We found that the divergence time between Asian and European dogs were ~17,000 years ago (95% CI: 11,700-21,000) when using golden jackal as outgroup, and ~14,200 years ago (95% CI: 10,700-16,600) when using Andean fox as an outgroup (Supplementary Figure 55).

Supplementary Note 13: Numerical estimation of the HXH/NGD-modern European divergence time

Summary of Numeric approach

As described above, estimating the divergence time of HXH and NGD using `G-PhoCS` could potentially lead to large biases due to false positive singleton variants observed in HXH that are caused by post-mortem damage and the somewhat lower coverage of our ancient samples. Therefore we devised a method that would be robust to these issues. In particular, we utilized demographic parameters estimated by `G-PhoCS` (τ and θ) that describe the relationship between European, Indian and East Asian village dogs, and then inferred the HXH/NGD divergence time by using coalescent theory to predict the ratio of shared derived sites between European village dogs and HXH/NGD versus European and Indian Village dogs. We first give the expectation considering a simple bifurcating tree for Europe, HXH/NGD and India, and then consider the expectation assuming East Asian admixture into HXH/NGD.

No Admixture

We assume the population tree shown in Supplementary Figure 24. Our main problem is false positives that appear as mutations on the branch leading to HXH/NGD after diverging from Europe. In order to limit this effect, we will condition on whether derived mutations (identified via an outgroup) present on a European chromosome but absent on an Indian chromosome are also present on one of the HXH/NGD chromosomes or not (110 sites). In addition, as HXH/NGD and Europe are sister clades versus India, there should be more such sites than those where mutations are shared between a European and Indian chromosome, but not a HXH/NGD chromosome (101 sites). This ratio should be approximately equal to the ratio of genealogies with these two topologies across the genome. To infer this we need to consider two types of parameters, the amount of population diversity, θ and the divergence time measured in expected numbers of mutations, □. With these we can use coalescent theory to predict the relative number of 110 versus 101 genealogies.

There are two scenarios that would lead to 110 sites. In the first there is a coalescent event, C, between a HXH/NGD and European lineage between □$_1$ and □$_2$, which will depend on □$_1$. Using standard coalescent theory [48], this will occur with the following probability:

$$P(C_{\tau_1<t<\tau_2}) = 1 - e^{-\frac{2}{\theta_1}(\tau_2-\tau_1)}$$

In addition, no coalescence could occur during the period after, which there is 1/3 chance that the next coalescent event will be between a HXH/NGD and European chromosomes (versus an European and Indian chromosome or a HXH/NGD and Indian chromosome). Thus the probability of observing a 110 compatible genealogy is:

$$P(110) = \left(1 - e^{-\frac{2}{\theta_1}(\tau_2-\tau_1)}\right) + \left(\frac{e^{-\frac{2}{\theta_1}(\tau_2-\tau_1)}}{3}\right)$$

Similarly, the probability of observing a 101 compatible genealogy is:

$$P(101) = \frac{e^{-\frac{2}{\theta_1}(\tau_2-\tau_1)}}{3}$$

The expected ratio is then simply *P*(110)/ *P*(101).

East Asian Admixture

We have found that HXH/NGD demonstrate evidence of small but statistically significant admixture with a population resembling modern Southeast Asian dogs, which may decrease the number of occasions where HXH/NGD and Europe chromosomes coalesce before an Indian chromosome compared to the expectation above. We denote this admixture fraction, □, and □ =1- □. The probability of observing a 110 now depends on whether the chromosome chosen in HXH/NGD traces its ancestry through East Asia or not. If it does not, then the probability of *P*(110) is simply the same as above multiplied by □. If it does, then the probability of a 110 compatible genealogy depends on whether the European and Indian chromosome coalesced between $□_2$ and $□_3$, which will depend on $□_2$ (i.e. essentially the reverse situation to before). Thus, the new total probability of a 110 genealogy is:

$$P(110) = \beta\left[\left(1 - e^{-\frac{2}{\theta_1}(\tau_2-\tau_1)}\right) + \left(\frac{e^{-\frac{2}{\theta_1}(\tau_2-\tau_1)}}{3}\right)\right] + \alpha\left[\frac{e^{-\frac{2}{\theta_2}(\tau_3-\tau_2)}}{3}\right]$$

Similarly, the new probability of a 101 genealogy is:

$$P(101) = \alpha\left[\frac{e^{-\frac{2}{\theta_1}(\tau_2-\tau_1)}}{3}\right] + \beta\left[\left(1 - e^{-\frac{2}{\theta_2}(\tau_3-\tau_2)}\right) + \left(\frac{e^{-\frac{2}{\theta_2}(\tau_3-\tau_2)}}{3}\right)\right]$$

Application and Advantages

We are interested in estimating the HXH/NGD-Europe split time, so $□_1$. Therefore we use estimates for $□_2$, $□_3$, $□_1$ (we assume the N_e of the boxer-European ancestral population is the same as that of the HXH/NGD-European ancestral population) and $□_2$ from the `G-PhoCS` analysis of modern samples and estimates □ from the *f4* ratio test. We then find the value of $□_1$ from our equations that is compatible with the observed 110/101 ratio. When comparing this expectation to real data it is is also assumed that sites (i.e. genealogies) are independent. To take into account dependence amongst linked sites we utilize a weighted block jackknife [49] to estimate confidence intervals.

Results

Using the whole genome SNP set, we computed the observed ratio of the number of SNPs where i) a European village dog and HXH have the derived allele and an Indian sample has the

ancestral allele versus ii) a European and Indian village dog have the derived allele and HXH has the ancestral allele. We took the allele with the highest probability from one sample of each population. We tried using either golden jackal or andean fox to determine the ancestral allele. However, we found estimates in the former to be somewhat higher than in the latter. The golden jackal is known to be admixed with wolf populations [25,50] while recurrent mutation on the longer andean fox lineage may cause underestimation. Therefore, we use the sites where the golden jackal and andean fox are concordant as the ancestral allele. In order to compute the confidence interval of this estimate, we took a weighted jackknife approach using windows of 10cM (Supplementary Table 17). The jackknife estimate of this ratio is 1.201485 for HXH, with standard error 0.016 (i.e. European dogs share more derived alleles with HXH a than Indian village dogs, as per expectations).

We sampled several parameters, namely N_e for European/boxer ancestral population (θ_1), N_e for European/Indian ancestral population (θ_2), time of divergence for Europe and boxer (τ_0), time of divergence for Europe/India (τ_2) and time of divergence for Europe-India/Asia (τ_3) based on the estimates from `G-PhoCS` analysis (Supplementary Table 23), and also the percentage of HXH that is made up of Asian admixture (α) from the *f4*-ratio analysis. We then sampled τ_1 from a uniform distribution of (τ_0,τ_2). We computed the ratio using the analytical formula explained above and kept 1000 τ_1 estimates if the ratio fell into the range: [1.185939,1.217031]. Based on this, the mean value of τ_1 was estimated as 1.3 x 10^{-5} and the 95% confidence interval as 8.6 x 10^{-6} to 1.7 x 10^{-} (Supplementary Figure 25A). If we assume that this divergence time is older than 7,000 years ago, then μ has an upper bound with mean value 5.6 x 10^{-9} per generation and 95% CI of 3.7 x 10^{-9} to 7.4 x 10^{-9} (Supplementary Figure 25C), which is consistent with the μ=4 x 10^{-9} per generation suggested by Skoglund *et al.* [51]. When using the μ=4 x 10^{-9} rate, the mean divergence time between HXH and European village dogs is 9,719 years ago, with a 95% CI of 6,483 to 12,910 years ago. We also tested our method by replacing HXH with Boxer and estimated the divergence time between boxer and European to be 8.84 x 10^{-6} (6,630 years ago) as the mean value and 4.1 x 10^{-6} to 1.41 x 10^{-5} as the 95% CI, while the mean `G-PhoCS` estimate of τ_0 was 8.5 x 10^{-6} (6,375 years ago) with a 95% CI of 5.76 x 10^{-6} - 1.24 x 10^{-5} (Supplementary Figure 25B, red as mean, blue as confidence intervals, dashed lines are the `G-PhoCS` estimates). We also estimated the divergence time between NGD and European village dogs, with mean divergence time 9,588 years ago with a 95% CI of 6,365 to 12,592 years ago, similar to the divergence time between HXH and European village dogs.

Supplementary Note 14: Haplotype and CNV analysis at domestication loci

Genotype matrices and NJ trees

Thirty-six putative domestication loci were previously identified by Axelsson *et al.* [52] as exhibiting significant differences in pooled heterozygosity (H_P) and F_{ST} in modern breed dogs compared to wolves [52]. Coordinates of the 30 significant autosomal F_{ST} windows were lifted over to the CanFam3.1 assembly coordinates, resulting in windows ranging from ~200kb to ~1.5Mb in length (Supplementary Table 18). We constructed visual genotype matrices for each locus to assess haplotype patterns at candidate selective loci. We extracted SNPs from within our whole genome SNP dataset (Call set 1, using the `PLINK` make-bed tool with no missing data filter) that were located in these windows, totalling 23,098 sites. For ease of viewing of each locus in matrix format, we further removed SNPs with minor allele frequencies less than 0.05 and greater than 0.49, resulting in 7,747 sites. `Eigenstrat` genotype file formats were generated per window using `convertf` from the `EIGENSOFT` package [53] and custom scripts were used to convert the genotype files into matrix formats for visualization using `matrix2png` [54].

Assignment of ancient dogs to either being more wild-like or dog-like relied upon putative domestication loci that confidently distinguished genotypes of dogs from wild canines (wolves, coyotes, jackals, etc.). To determine whether swept dog haplotypes could be clearly defined for each F_{ST} locus defined in Axelsson *et al.* [52], we first calculated the proportion of reference (or "dog") alleles versus non-reference (putatively wild) alleles per sample using the SNP set without minor allele frequency filtration. These estimates were obtained for each window. If 25% or more dogs exhibited an average reference allele count less than 95% (i.e. more wild-like) or if less than 75% of wolves and other outgroups had an average reference allele count greater than 95% (i.e. more dog-like), the locus failed to reach the minimum requirement. One exception was made for F_{ST} window 23 (chr16: 6828779-7342805) because a strong, clear sweep can be distinguished that maintains previous requirements within a subset of the window (chr16: 7108869-7342805). Finally, the three ancient dog samples were classified as being more dog or wild-like if the sample had an average reference allele count <95% in a F_{ST} window that passed previous filtration steps (above).

Finally, NJ trees were estimated for each domestication locus with the filtered SNP sets (without minor allele frequency filtration) using the same methods as the whole-genome tree estimation and rooting with the Andean fox (Supplementary Note 8), but with genomic window sizes of 0.01 cM. We note that the bootstrap support within the dog clade is low because of the high identity among the dog haplotypes at these loci, which have likely undergone strong selective sweeps. Additionally, due to rapid, recent expansion in dogs since breed formation, visualizing relationships within the dog clade is difficult due to the collapsing of the tree branches. For this reason, each NJ tree has been drawn with proportional branches to simplify viewing relationships among the samples. Only a subset of the loci are detailed in this Supplementary Methods section, but trees and underlying data for all loci are available on request.

Copy Number Estimation

Copy-number was estimated from mapped read depth using methods previously developed for the analysis of human copy-number variation [55,56]. To tabulate read depth, we divided reads into non-overlapping 36-bp segments and mapped them to a masked version of the CanFam3.1 reference using `mrsFAST` [57] with an edit-distance threshold of two. Since `mrsFAST` returns all possible placements of a read, we performed aggressive masking of the reference genome prior to mapping. All elements identified by `RepeatMasker` or `Tandem Repeat Finder` [58] were masked out. Since the repeat classification of the genome may be incomplete, we additionally masked out over-represented 50 mers, defined by searching a sliding window of 50-bp (with an overlapping step size of 5) against the genome and removing all sequences with at least 20 hits within an edit distance of two. Due to a shadow effect of introduced gaps by read mapping, we further padded these masked segments by 36 bp on each side, resulting in the ability to interrogate 48% of the genome. For GC-correction and the conversion of read depth to copy-number, we defined a set of autosomal control regions that excluded sites reported to be duplicated or copy-number variable [25,59–61]. A GC-correction curve was determined based on a loess fit of read depth and GC content within 400 bp of each position. Copy-number was estimated based on the mean depth in non-overlapping windows each consisting of 3kb of unmasked sequence. Amylase copy-numbers were estimated using the window chrUn_AAEX03020568:4873-8379, which overlaps the 5' end of the *AMY2B* gene model.

Results

<u>Candidate Domestication Regions</u>

We note that clear distinctions in haplotypes between the dogs and wolves was not observed in twelve of the thirty windows (F_{ST} windows 6, 7, 8, 14, 15, 16, 22, 24, 27, 29, 30, and 32 failed). This has likely occurred for two possible reasons. First, these previously calculated windows were identified by significant F_{ST} deviations through comparisons of breed dogs and wolves only, without the inclusion of genetically diverse village dogs that have historically undergone less artificial selection resulting from breed development [31,62]. Second, the original study [52] was completed on an older canine reference (CanFam2). Subsequent structural rearrangements in the newer dog assembly (CanFam3.1) may have either (i) eliminated the signal originally observed or (ii) added sequence that that may not have been under selection, which can be observed in the increased size of the windows relative to the original study (Supplementary Table 18). To address the second concern, we have focused on regions that have maintained clear dog versus wolf haplotypes while disregarding the "variable" regions when genotyping the ancient samples as either more like modern dogs or wild canids (Supplementary Table 19).

Two patterns become evident upon inspection of haplotype patterns at the eighteen loci that passed initial filtration. First, there is a clear distinction between breed (blue; 0/0) and wolf (orange; 1/1) haplotypes, with heterozygotes (white) typically enriched in village dogs in the middle of each matrix (see Figure 6a). This is expected given the methods used by Axelsson *et al.* [52] to identify these loci. The "wild" (non-reference) haplotypes are often found in New and Old World wolves, coyotes, and the golden jackal. The haplotypes of the three ancient dogs (top rows) appear to be more closely related to breeds and village dogs than to wolves (results for all loci are available on request). In loci that deviate from this pattern, the older ancient dog (HXH) carries the "wild-like" haplotype in only one window (F_{ST} window 13), whereas CTC more often

is heterozygous for the a "wild" haplotype ("white"; six windows total) (Supplementary Table 19). This is consistent with our additional population-level analyses that identified considerable CTC admixture with wolf. Additionally, the younger, Irish ancient dog (NGD) is heterozygous for the wild-like haplotypes in two windows. These results are mirrored in the estimated NJ trees, where most topologies place the ancient samples in the dog clade, with more examples of CTC grouping with wild canids than HXH and NGD.

From previous genome-wide selection scans between dogs and wolves, genomic windows under significant selection appear to be enriched for genes involved in starch metabolism and behavior [52,63]. Three loci that were highlighted in the analyses of Axelsson *et al.* [52] harbor genes critical for starch digestion in canines, and were putatively linked to the domestication of wolves as detected by selective sweeps (significant deviation in F_{ST}) between breed dogs and wolves. Two of these three regions (F_{ST} windows 12 and 23) were successfully lifted over from the previous dog reference assembly (CanFam2) to the updated CanFam3.1, whereas the F_{ST} region on chr26 failed (F_{ST} window 31) due to lack of SNP support.

The first stage of starch processing in canines involves the pancreatic alpha-amylase gene (*AMY2B*) which is harbored in F_{ST} window 12 on chromosome 6. This gene is usually considered the hallmark for selection from domestication, where increases in copy-number for *AMY2B* may have provided a selective advantage for enhanced abilities for starch digestion [25,52,64]. Reference allele frequencies highlight the differences between dogs and wolves in this window, with wild canids and wolves (excluding the Spanish wolf) carrying fewer dog alleles compared to dogs (Supplementary Figure 56). Our results indicate a clear dog haplotype in the genotype matrices for regions surrounding the *AMY2B* gene locus that are shared between both breeds and village dogs (Figure 6a). In contrast, wolves and other canids (jackal, fox, and coyotes) primarily have the "wild" haplotype in this region. Distinct dog exceptions include the standard poodle (osp), the Siberian husky, the dingo, and the Late Neolithic dog, CTC. Intriguingly, the older of the ancient dogs (HXH, early Neolithic) carries the "dog" haplotype. Exhibiting heterozygosity near the *AMY2B*, the NGD haplotype is similar to the mastiff, bulldog, toy poodle, and some village dogs, making it "wild-like". Consistent with earlier results that suggest recent admixture with dogs in the sample (Supplementary Note 10), the wild outlier here is the Spanish wolf (spw), which appears to be largely heterozygous in this window, reflecting the observed higher proportion of reference alleles in the window (Supplementary Figure 56).

From the NJ tree of the whole SNP set for the *AMY2B* window (Supplementary Figure 57), we observe a distinct wild clade (BS = 100) that contains wolves, coyotes, and the jackal. Amongst the Old World wolves, the dog outliers (dingo and standard poodle) are clustered, mirroring their haplotype patterns in the genotype matrix (Figure 6a). CTC is confidently placed as sister to all dogs (BS = 91), highlighting the "wild" component in the CTC haplotype at this locus (0.850 observed reference allele proportion; Supplementary Table 19). The Spanish wolf is also placed in a basal position, sister to dogs, but with low BS support (BS = 26), likely due to the level of detectable dog admixture in this wolf. NGD (0.851 observed average reference allele count) is placed in a clade with the Siberian husky and a North Chinese village dog (Dog04; BS = 54), at basal positions in the dog clade. Finally, HXH is positioned well within the dog (breeds and village dogs) clade, and sister to the Mexican hairless dog, the xoloitzcuintli (BS = 87). Interestingly, none of the ancient dogs carry the extreme copy-number expansion of *AMY2B*,

despite being either homozygous or heterozygous for the dog haplotype (Figure 6).

To further define the extent of haplotype sharing at this locus among modern and ancient samples, we performed an IBD analysis using the Refined IBD algorithm in `Beagle v 4.1` [65]. Analysis was limited to village dogs and ancient dog samples, and only sites with a minor allele frequency >= 0.05 were considered. We utilized the genetic map from Auton *et al.* [23] and ran Refined IBD with options: `chrom=6, window=50000, overlap=2000, ibdtrim=50`. Analysis was repeated ten times with different random seeds and results were combined using `ibdmerge.jar`. We identified 31 IBD segments that intersected with the *AMY2B* region (F_{ST} Window 12), two of which involved at least one of the ancient dogs (one between HXH and QA5, and another between HXH and NGD). The 29 IBD segments among the village dogs have a median length of 0.6796 cM (763.6 kbp), while the HXH-QA5 segment has a length of 0.6132 cM (582.1 kbp), and the HXH-NGD segment has a length of 0.3534 cM (457.2 kb). Although these approaches have a limited ability to detect short IBD segments, this analysis suggests that the shared haplotypes around *AMY2B* extend for longer distances among the contemporary samples, consistent with a more recent sweep at this locus driven by *AMY2B* copy number.

The second stage of starch digestion involves the activity of *MGAM* (maltase-glucoamylase), which facilitates the conversion of oligosaccharides to glucose as part of a larger metabolic pathway. According to the genotype matrix for this locus, the *MGAM* gene is located in the less variable region of F_{ST} Window 23 (Supplementary Figure 58), along with the bitter taste mediating receptor (*TAS2R38*) and a C-type lectin domain family 5 gene (*CLEC5A*). Again, this region highlights a locus that has likely undergone selective sweeps, as indicated by pronounced differentiation between the dog and wild haplotypes. For the assignment of dog and wild haplotypes, average reference allele counts were calculated separately in the less variable portion of the window (chr16: 7125960-7303209), where the selective sweep is apparently more pronounced (Supplementary Figure 59). Again, clear delineations of reference allele counts can be observed between the wolves and other canids, compared to both breed and village dogs. Exceptions of this pattern include the two Mexican wolves, and a handful of dogs. Unlike the previous locus, all three ancient samples carry the dog haplotypes with 0.994, 0.996, and 0.997 in HXH, CTC, and NGD, respectively. This result is also supported from the resulting NJ tree (from all SNPs in the window) that indicates both CTC and HXH firmly within the dog clade (Supplementary Figure 60). The tree also strongly supports monophyletic wolf/coyote (excluding the Mexican wolves also observed to deviate in Supplementary Figure 59) and dog clades with a bootstrap score of 93, which emphasizes that the time of this significant sequence divergence between dogs and wild canines pre-dates, at least, 7,000 years before present, since HXH carries the dog haplotype.

AMY2B Copy Number Variation

In contrast to HXH and CTC, and the results of Frantz *et al.* [22], our analysis estimates that NGD carried three copies of the *AMY2B* gene. Several differences in the methods for copy number estimations exist between this study and Frantz *et al.*[22], including correction for local GC content and the exclusion of known CNVs from regions used to normalize read depth.

Closer examination of the estimated copy number profiles revealed the presence of a larger, ~2

megabase duplication that overlaps the *AMY2B* locus on chromosome 6 and extends proximally (Supplementary Figure 26). This duplication is present in eleven of the samples we analyzed. NGD is heterozygous for this duplication, but does not carry the extreme *AMY2B* copy number increase that is presumed to arise from tandem expansion of the gene region [52].

Supplementary Note 15: Comparison with Newgrange dog analysis in Frantz *et al.*

We incorporated the recently published NGD [22] into our major analyses. However, we identified multiple discrepancies between our results and those of Frantz *et al.* [22]. Below we describe the major differences in analysis and conclusions.

PCA

Frantz *et al.* [22] performed a PCA on a genome-wide data set, with and without transitions (Figure S8 and Figure S9 in Frantz *et al.* [22]). They find that NGD is located outside of the modern dogs clusters in the PC space and thus suggested that "*the Newgrange dog may retain some degree of ancestry from an ancient (extinct) European population*". This result is in contrast to our PCA of NGD, CTC, and HXH. We found that the observation of NGD being an outlier is a technical artifact that occurred when the PCA was performed on both the uncalibrated and calibrated ancient genome sample in the same analysis. This results in extremely high covariance between the duplicate sample call sets, which caused them to dominate that second PC. We replicated the PCA using the same 269,512 transversions ascertained solely in the genome-wide data-set from Frantz *et al.* [22], with and without doubling the NGD dog (Supplementary Figure 11). We found that NGD moves back into the general dog cluster in the Frantz *et al.* [22] sample set, next to European village dogs and modern breeds, when only one NGD call set was used, a result similar to what we observed previously (Supplementary Note 9).

However, even with such corrections, this PCA still differs from our previous PCA as the two Russian wolves appear to cluster amongst other dogs, and the population structure between breeds and village dogs is largely absent. To address this discrepancy, we performed a PCA using the exact SNP sites as Frantz *et al.* [22], but extracted the calls from our sample set. Only then could we obtain a similar structure that corresponded with our other PCAs (Supplementary Figure 12). Therefore, in addition to the technical error regarding sample duplicates, the overall ascertainment of samples also contributes to the differences in PCA plots and our failure to replicate the PCA. Since our dataset contains a substantially more diverse collection of samples, which includes various breeds and village dogs from all over the world, we assert that our PCA results are more reflective of total dog diversity.

Divergence time between dogs

Frantz *et al.* [22] used an MSMC approach to estimate the split time between Asian and non-Asian dogs. However, MSMC requires phased haplotypes from all samples. Statistical phasing errors in human haplotypes result in a more recent split time estimates [66]. The performance of statistical phasing on dog genomes is not quantified but the size of the dog reference panel is less than 10% of the size available for human data. Our study and Freedman *et al.* and Wang *et al.* obtained Asian vs non-Asian dog divergence time estimates over 20,000 years ago when using the same mutation rate (4×10^{-9}/generation) and generation time (3 years) (Supplementary Table 16). Since the earliest dog fossil remains are dated to be 15,000 years old in Western Eurasia (Europe and the Near East), the divergence time between Asian and non-Asian dogs occurring 20,000 years ago does not support two domestication processes

In addition, potential genotyping errors due to post-mortem damage may also cause biases when utilizing the NGD to estimate a divergence time with Asian dogs, which they find has a slightly older divergence compared to using modern European dogs and suggest is evidence of ancestry from the remnant European Paleolithic dog population. Fig S26 in Frantz *et al.* [22] notably demonstrates that the Tv/Ti heterozygote ratio for NGD is lower than all but one of their contemporary canid genomes, suggesting even with base quality recalibration, false positive transitions (C>T and G>A) due to post-mortem damage may still be prominent in the inferred sequence for the NGD, which would likely lead to increased divergence times by artificially elongating the branch lengths for NGD.

Comparison to Frantz *et al.* 2016 ADMIXTUREGRAPH analysis

Frantz *et al.* [22] recently fit an admixture model that did not explicitly model Southeast Asian gene flow into NGD, but instead modelled a) wolf and ancestral East Asian dog admixture (Fig. S14 of their paper) and b) an additional admixture event from East Asian dogs into modern European dogs (Fig S24 of their paper). However, we failed to replicate the fit of either of these models for NGD, using various European and Chinese wolves to represent the "Wolf" population in Frantz *et al.* [22]. We obtained 28-31 outliers with *Z* scores as high as ~46. Frantz *et al.* [22] utilized Russian wolves, which based on Fan *et al.* [27] have a common ancestry with Asian wolves, such as our Chinese wolf. Since our results are consistent with various other analyses (`Admixture`, `NGSadmix`, `MixMapper`, `PCA`), we speculate that the result of Frantz *et al.* may be due to their unusual SNP ascertainment.

Supplementary References

1. Boulestin, B. & Coupey, A.-S. *Cannibalism in the Linear Pottery Culture: The Human Remains form Herxheim*. (Archaeopress Publishing LTD, 2015).
2. Arbogast, R.-M. *et al.* Du loup au „chien des tourbières ". Les restes de canidés sur les sites lacustres entre Alpes et Jura. *Revue de Paléobiologie* **10,** 171–183 (2005).
3. Seregély, T., Burgdorf, P., Gresik, G., Müller, M. S. & Wilk, A. „Tote Menschen und Tiere in finsteren Felsschächten …" -neue Dokumentationsmethodik und erste Untersuchungsergebnisse zur Kirschbaumhöhle in Oberfranken. *Praehistorische Zeitschrift* **90,**
4. Studer, T. Die prähistorischen Hunde in ihrer Beziehung zu den gegenwärtig lebenden Rassen. (1901).
5. Schibler, J. *Ökonomie und Ökologie neolithischer und bronzezeitlicher Ufersiedlungen am Zürichsee: Ergebnisse der Ausgrabungen Mozartstrasse, Kanalisationssanierung Seefeld, AKAD/Pressehaus und Mythenschloss in Zürich*. **20,** (Direktion der Öffentlichen Bauten des Kantons Zürich, Hochbauamt, Abt. Kantonsarchäologie, 1997).
6. Yang, D. Y., Eng, B., Waye, J. S., Dudar, J. C. & Saunders, S. R. Technical note: improved DNA extraction from ancient bones using silica-based spin columns. *Am. J. Phys. Anthropol.* **105,** 539–543 (1998).
7. MacHugh, D. E., Edwards, C. J., Bailey, J. F., Bancroft, D. R. & Bradley, D. G. The extraction and analysis of ancient DNA from bone and teeth: a survey of current methodologies. *Anc. Biomol.* **3,** 81–102 (2000).
8. Meyer, M. & Kircher, M. Illumina sequencing library preparation for highly multiplexed target capture and sequencing. *Cold Spring Harb. Protoc.* **2010,** db.prot5448 (2010).
9. Gamba, C. *et al.* Genome flux and stasis in a five millennium transect of European prehistory. *Nat. Commun.* **5,** 5257 (2014).

10. Martin, M. Cutadapt removes adapter sequences from high-throughput sequencing reads. *EMBnet.journal* **17,** 10–12 (2011).
11. Langmead, B. & Salzberg, S. L. Fast gapped-read alignment with Bowtie 2. *Nat. Methods* **9,** 357–359 (2012).
12. Li, H. & Durbin, R. Fast and accurate short read alignment with Burrows-Wheeler transform. *Bioinformatics* **25,** 1754–1760 (2009).
13. Bramanti, B. *et al.* Genetic discontinuity between local hunter-gatherers and central Europe's first farmers. *Science* **326,** 137–140 (2009).
14. Scheu, A. *et al.* The genetic prehistory of domesticated cattle from their origin to the spread across Europe. *BMC Genet.* **16,** 54 (2015).
15. Hofmanová, Z. *et al.* Early farmers from across Europe directly descended from Neolithic Aegeans. *Proc. Natl. Acad. Sci. U. S. A.* (2016). doi:10.1073/pnas.1523951113
16. Kircher, M., Sawyer, S. & Meyer, M. Double indexing overcomes inaccuracies in multiplex sequencing on the Illumina platform. *Nucleic Acids Res.* **40,** e3 (2012).
17. Kircher, M. Analysis of high-throughput ancient DNA sequencing data. *Methods Mol. Biol.* **840,** 197–228 (2012).
18. Lassmann, T., Hayashizaki, Y. & Daub, C. O. TagDust—a program to eliminate artifacts from next generation sequencing data. *Bioinformatics* **25,** 2839–2840 (2009).
19. McKenna, A. *et al.* The Genome Analysis Toolkit: a MapReduce framework for analyzing next-generation DNA sequencing data. *Genome Res.* **20,** 1297–1303 (2010).
20. Ginolhac, A., Rasmussen, M., Gilbert, M. T. P., Willerslev, E. & Orlando, L. mapDamage: testing for damage patterns in ancient DNA sequences. *Bioinformatics* **27,** 2153–2155 (2011).
21. Briggs, A. W. *et al.* Patterns of damage in genomic DNA sequences from a Neandertal. *Proc. Natl. Acad. Sci. U. S. A.* **104,** 14616–14621 (2007).
22. Frantz, L. A. F. *et al.* Genomic and archaeological evidence suggest a dual origin of

domestic dogs. *Science* **352,** 1228–1231 (2016).

23. Auton, A. *et al.* Genetic recombination is targeted towards gene promoter regions in dogs. *PLoS Genet.* **9,** e1003984 (2013).
24. De Decker, S. *et al.* Tethered cord syndrome associated with a thickened filum terminale in a dog. *J. Vet. Intern. Med.* **29,** 405–409 (2015).
25. Freedman, A. H. *et al.* Genome sequencing highlights the dynamic early history of dogs. *PLoS Genet.* **10,** e1004016 (2014).
26. Schoenebeck, J. J. *et al.* Variation of BMP3 contributes to dog breed skull diversity. *PLoS Genet.* **8,** e1002849 (2012).
27. Fan, Z. *et al.* Worldwide patterns of genomic variation and admixture in gray wolves. *Genome Res.* **26,** 163–173 (2016).
28. Decker, B. *et al.* Comparison against 186 canid whole-genome sequences reveals survival strategies of an ancient clonally transmissible canine tumor. *Genome Res.* **25,** 1646–1655 (2015).
29. Li, G. *et al.* Comparative analysis of mammalian Y chromosomes illuminates ancestral structure and lineage-specific evolution. *Genome Res.* **23,** 1486–1495 (2013).
30. Anderson, T. M. *et al.* Molecular and evolutionary history of melanism in North American gray wolves. *Science* **323,** 1339–1343 (2009).
31. Shannon, L. M. *et al.* Genetic structure in village dogs reveals a Central Asian domestication origin. *Proc. Natl. Acad. Sci. U. S. A.* **112,** 13639–13644 (2015).
32. Pilot, M. *et al.* On the origin of mongrels: evolutionary history of free-breeding dogs in Eurasia. *Proc. Biol. Sci.* **282,** 20152189 (2015).
33. Thalmann, O. *et al.* Complete mitochondrial genomes of ancient canids suggest a European origin of domestic dogs. *Science* **342,** 871–874 (2013).
34. Tamura, K., Stecher, G., Peterson, D., Filipski, A. & Kumar, S. MEGA6: Molecular Evolutionary Genetics Analysis version 6.0. *Mol. Biol. Evol.* **30,** 2725–2729 (2013).

35. Duleba, A., Skonieczna, K., Bogdanowicz, W., Malyarchuk, B. & Grzybowski, T. Complete mitochondrial genome database and standardized classification system for Canis lupus familiaris. *Forensic Sci. Int. Genet.* **19,** 123–129 (2015).
36. Gronau, I., Hubisz, M. J., Gulko, B., Danko, C. G. & Siepel, A. Bayesian inference of ancient human demography from individual genome sequences. *Nat. Genet.* **43,** 1031–1034 (2011).
37. Junier, T. & Zdobnov, E. M. The Newick utilities: high-throughput phylogenetic tree processing in the UNIX shell. *Bioinformatics* **26,** 1669–1670 (2010).
38. Wang, G.-D. *et al.* Out of southern East Asia: the natural history of domestic dogs across the world. *Cell Res.* **26,** 21–33 (2016).
39. Patterson, N., Price, A. L. & Reich, D. Population structure and eigenanalysis. *PLoS Genet.* **2,** e190 (2006).
40. Purcell, S. *et al.* PLINK: a tool set for whole-genome association and population-based linkage analyses. *Am. J. Hum. Genet.* **81,** 559–575 (2007).
41. Bradburd, G., Ralph, P. L. & Coop, G. *A Spatial Framework for Understanding Population Structure and Admixture*. (2015). doi:10.1101/013474
42. Alexander, D. H., Novembre, J. & Lange, K. Fast model-based estimation of ancestry in unrelated individuals. *Genome Res.* **19,** 1655–1664 (2009).
43. Skotte, L., Korneliussen, T. S. & Albrechtsen, A. Estimating individual admixture proportions from next generation sequencing data. *Genetics* **195,** 693–702 (2013).
44. Patterson, N. *et al.* Ancient admixture in human history. *Genetics* **192,** 1065–1093 (2012).
45. Lipson, M. *et al.* Efficient moment-based inference of admixture parameters and sources of gene flow. *Mol. Biol. Evol.* **30,** 1788–1802 (2013).
46. Fu, Q. *et al.* The genetic history of Ice Age Europe. *Nature* **534,** 200–205 (2016).
47. Rambaut, A., Suchard, M. A., Xie, D. & Drummond, A. J. Tracer v1. 6. (2014).
48. Hudson, R. R. & Others. Gene genealogies and the coalescent process. *Oxford surveys in*

evolutionary biology **7,** 44 (1990).

49. Busing, F. M. T. A., Meijer, E. & Van Der Leeden, R. Delete-m Jackknife for Unequal m. *Stat. Comput.* **9,** 3–8 (1999).
50. Koepfli, K.-P. *et al.* Genome-wide Evidence Reveals that African and Eurasian Golden Jackals Are Distinct Species. *Curr. Biol.* **25,** 2158–2165 (2015).
51. Skoglund, P., Ersmark, E., Palkopoulou, E. & Dalén, L. Ancient wolf genome reveals an early divergence of domestic dog ancestors and admixture into high-latitude breeds. *Curr. Biol.* **25,** 1515–1519 (2015).
52. Axelsson, E. *et al.* The genomic signature of dog domestication reveals adaptation to a starch-rich diet. *Nature* **495,** 360–364 (2013).
53. Price, A. L. *et al.* Principal components analysis corrects for stratification in genome-wide association studies. *Nat. Genet.* **38,** 904–909 (2006).
54. Pavlidis, P. & Noble, W. S. Matrix2png: a utility for visualizing matrix data. *Bioinformatics* **19,** 295–296 (2003).
55. Alkan, C. *et al.* Personalized copy number and segmental duplication maps using next-generation sequencing. *Nat. Genet.* **41,** 1061–1067 (2009).
56. Sudmant, P. H. *et al.* Diversity of human copy number variation and multicopy genes. *Science* **330,** 641–646 (2010).
57. Hach, F. *et al.* mrsFAST: a cache-oblivious algorithm for short-read mapping. *Nat. Methods* **7,** 576–577 (2010).
58. Benson, G. Tandem repeats finder: a program to analyze DNA sequences. *Nucleic Acids Res.* **27,** 573–580 (1999).
59. Nicholas, T. J. *et al.* The genomic architecture of segmental duplications and associated copy number variants in dogs. *Genome Res.* **19,** 491–499 (2009).
60. Nicholas, T. J., Baker, C., Eichler, E. E. & Akey, J. M. A high-resolution integrated map of copy number polymorphisms within and between breeds of the modern domesticated dog.

BMC Genomics **12,** 414 (2011).

61. Chen, W.-K., Swartz, J. D., Rush, L. J. & Alvarez, C. E. Mapping DNA structural variation in dogs. *Genome Res.* **19,** 500–509 (2009).
62. Boyko, A. R. *et al.* Complex population structure in African village dogs and its implications for inferring dog domestication history. *Proc. Natl. Acad. Sci. U. S. A.* **106,** 13903–13908 (2009).
63. Cagan, A. & Blass, T. Identification of genomic variants putatively targeted by selection during dog domestication. *BMC Evol. Biol.* **16,** 10 (2016).
64. Arendt, M., Fall, T., Lindblad-Toh, K. & Axelsson, E. Amylase activity is associated with AMY2B copy numbers in dog: implications for dog domestication, diet and diabetes. *Anim. Genet.* **45,** 716–722 (2014).
65. Browning, B. L. & Browning, S. R. Improving the accuracy and efficiency of identity-by-descent detection in population data. *Genetics* **194,** 459–471 (2013).
66. Song, S., Sliwerska, E., Emery, S. & Kidd, J. M. Modeling human population separation history using physically phased genomes. *Genetics* **205,** 385–395 (2017).
